# Photochemical Uncaging of Aldehydes and Ketones via Photocyclization/Fragmentation Cascades of Enyne Alcohols: An Unusual Application for a Cycloaromatization Process

**DOI:** 10.3390/molecules28155704

**Published:** 2023-07-28

**Authors:** Adam Campbell, Nikolas R. Dos Santos, Igor Alabugin

**Affiliations:** Department of Chemistry and Biochemistry, Florida State University, Tallahassee, FL 32306, USA; atcampbell@fsu.edu (A.C.); nd18e@fsu.edu (N.R.D.S.)

**Keywords:** photoremovable protecting groups, photouncaging, cycloaromatization, aldehyde synthesis, ketone synthesis

## Abstract

We utilized a cycloaromatization reaction driven by relief of excited state antiaromaticity to photouncage aldehydes and ketones. We developed several synthetic routes towards the synthesis of photocaged carbonyls as allylically substituted 3-(2-(arylethynyl)phenyl)prop-2-en-1-ols. A library of photocaged aryl aldehydes and ketones containing donors and acceptors, as well as several photocaged fragrance aldehydes and the steroid 5α-cholestan- 3 -one, were synthesized and demonstrated photouncaging in good to excellent yields.

## 1. Introduction

**Figure 1:**
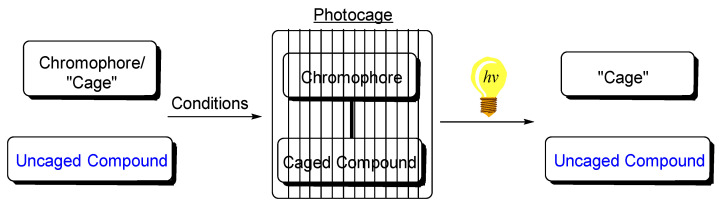
General description of how photocages work.

One of the greatest challenges of chemistry is being able to control chemical transformations in complex biological environments. Photocages, molecules that cage compounds with photoremovable protecting groups, represent a useful strategy to introduce molecules to a system “on demand” with precise temporal and spatial control. The advantage of photocaging is that it protects the functional group from conditions where it is not stable until its arrival at the destination, where it can be uncaged upon irradiation (Figure 1).

**Scheme 1:**
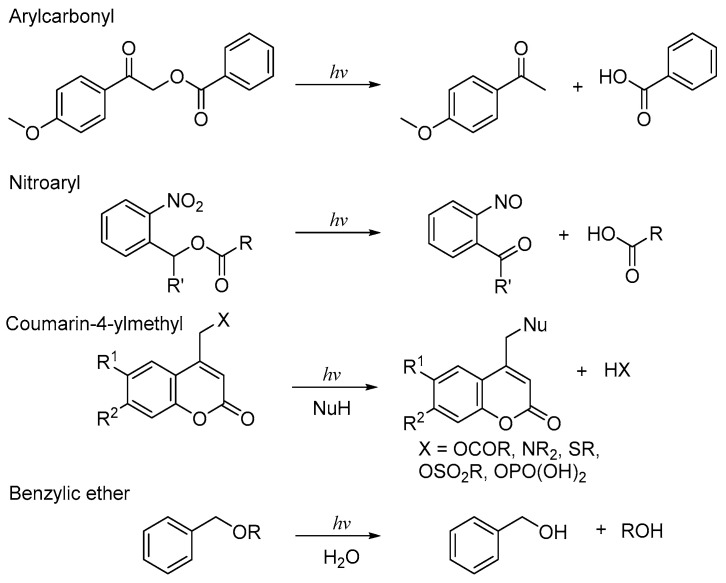
Selected Photocages.

A variety of photoremovable protective groups can release different types of functionalities upon irradiation. Some of the most common photocages include arylcarbonyl [1,2], nitroaryl [3,4], coumarin-4-ylmethyl [5,6], and benzylic ethers [7,8] (Scheme 1).

Herein, we report the utility of allylically substituted 3-(2-(arylethynyl)phenyl)prop-2-en-1-ols (subsequently referred to here as enynols) as caged ketones and aldehydes, as well as their 1-step synthesis from a common precursor. Furthermore, photouncaging was promoted by antiaromaticity relief [9,10,11,12] as an unusual driving force. It is also notable that ketones and aldehydes (especially the aryl varieties) are good chromophores themselves (often functioning as photosensitizers or photoremovable protecting groups) and are still released cleanly under the photochemical conditions reported herein. We believe this method helps to address the relative scarcity of release protocols for carbonyl compounds, which typically employ acetals [13], ketals [14,15], or dithianes [16].

The enynols were previously used in our group in a quest to discover the C1-C5 cycloaromatization reaction that converts aromatic enynes into benzofulvenes [17]. The reaction is driven by the relief of excited state antiaromaticity [18,19] and is terminated by the release of formaldehyde. Such a reaction follows a unique mechanistic path of beginning with a closed shell starting material and transiently crossing to a diradical path where the final intermediate self-terminates with the clean formation of a carbonyl group.

**Scheme 2:**
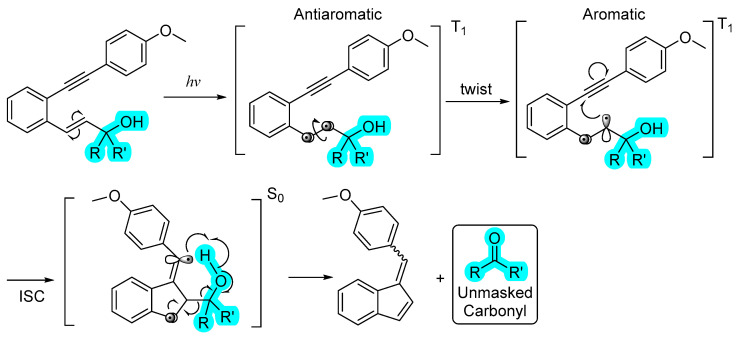
Photouncaging Mechanism Initiated by C1–C5 Cycloaromatization.

We wondered if the scope of this process could be expanded to release carbonyl compounds other than formaldehyde, thus functioning as a general source of photochemically uncaged carbonyl equivalents. In this paper, our focus is on the photorelease of aldehydes and ketones. The identity of such a caged carbonyl would be determined by the substitution at the allylic position (R, R′, Scheme 2) of the enynol. If one of these substituents is hydrogen, an aldehyde would be released. When both R and R’ are carbon chains or rings, the uncaging sequence would generate a ketone.

## 2. Results and Discussion

### 2.1. Synthesis

**Scheme 3:**
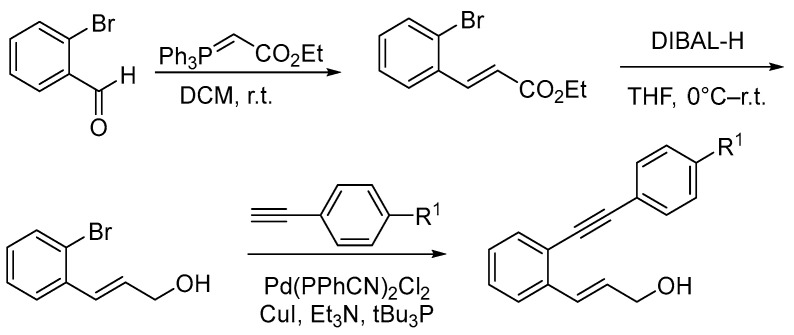
Previous Sonogashira Route to Enynols.

While the previous formaldehyde-releasing enynols could be synthesized via a simple Wittig-Sonogashira route (Scheme 3), we had to redesign the synthetic route to access more substituted enols. The photochemical uncaging of formaldehyde proceeded from primary alcohol prepared by reduction of the ester moiety. To release more substituted carbonyls in this same manner, the alcohol can no longer be the primary. Since the identity of the carbonyl to be released is defined by substitution at the allylic carbon, we needed enols, which cannot be synthesized through simple reduction of an ester and require a modified synthetic approach.

**Scheme 4:**
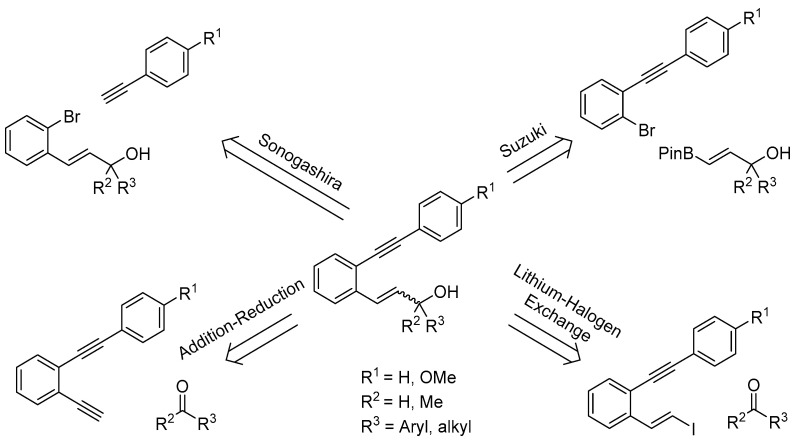
General Retrosynthetic Routes to Enynols.

Below, we describe three additional routes towards the synthesis of the enynols (Scheme 4). As previously described, the Sonogashira pathway (Scheme 4, top left) is a 3-step route to caged carbonyls. This pathway is not modular, as it requires the synthesis of a different ortho-bromo-enol precursor for each new carbonyl group to be uncaged. Considering this, we explored three different routes that include the late addition of a diverse library of aldehydes and ketones to a suitable nucleophilic precursor.

**Scheme 5:**
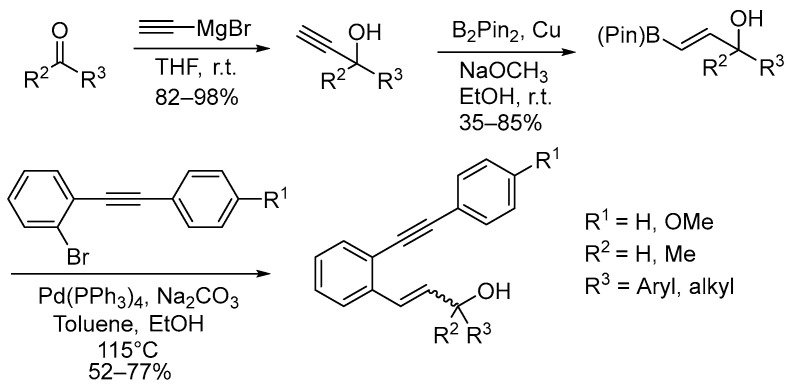
Suzuki Route Towards Substituted Enynols.

As an alternative, we assembled the enynols via the Suzuki reaction of a 2-bromo diphenylacetylene with borylated allylic alcohols. The latter can be obtained by borylation of propargylic alcohols, readily available from a Grignard reaction of ethynyl magnesium bromide with the carbonyl to be caged (Scheme 5).

Several photocaged carbonyls were synthesized through this route and gratifyingly showed the desired photouncaging upon irradiation (vide infra). However, this route suffered from requiring derivatization at the first step and from the varying yields and conditions required in the subsequent borylation step. In addition, it required four steps overall to access each photocaged compound. Thus, it was desirable to devise a more streamlined route.

**Scheme 6:**
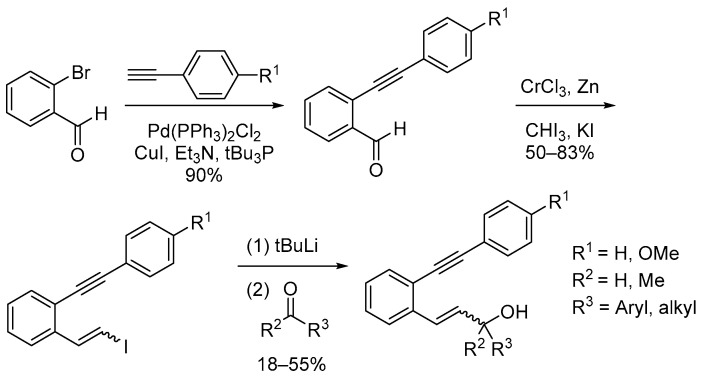
Vinyl Lithium Route Towards Substituted Enynols.

To that end, we developed a route beginning with the Sonogashira reaction of 2-bromobenzaldehyde to a phenylacetylene (see the Supplementary Materials). A Takai reaction [20] of the resulting aldehyde gave a vinyl iodide precursor (Scheme 6). Lithiation of the vinyl iodide with t-BuLi [21] provides the nucleophile for the carbonyl to be caged. While yields of the final step are moderate to low, this route represents an overall improvement to the previous route, which required only two steps to prepare a common precursor. Once this precursor is available, it can be used to access a variety of photocaged aldehydes and ketones in a single step. 

**Scheme 7:**
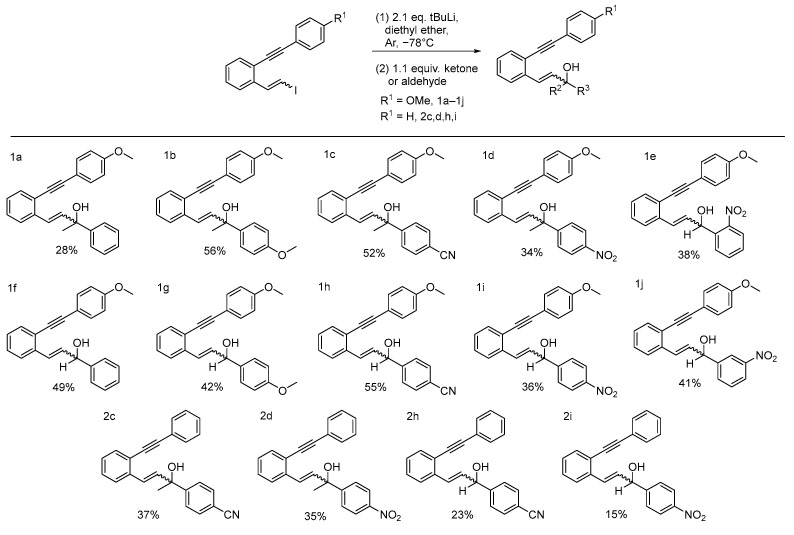
Scope of The Vinyl Lithium-Mediated Approach to Photocaged Carbonyls.

Unfortunately, yields to the final step are moderate. The addition of OMePh-substituted ortho-ethynyl vinyl lithium (R^1^ = OMe) to benzophenones (R^2^ = Me, R^3^ = Ar) and aryl aldehydes (R^2^ = H, R^3^ = Ar) proceeded in 28–56% and 36–56%, respectively (Scheme 7). Yields for the Ph-substituted analog (R^1^ = H) were similar, ranging from 15 to 37%.

Considering that the moderate yield may originate from intermolecular (5-exo-dig and 6-endo-dig) cyclization of vinyl lithium at the adjacent alkyne, we have also explored the reaction of readily accessible acetylide anions with carbonyls, where such side reactions are unlikely. We initially avoided such an approach due to the uncertainty about the chemoselectivity of alkyne reduction in a system with two alkyne moieties.

**Scheme 8:**
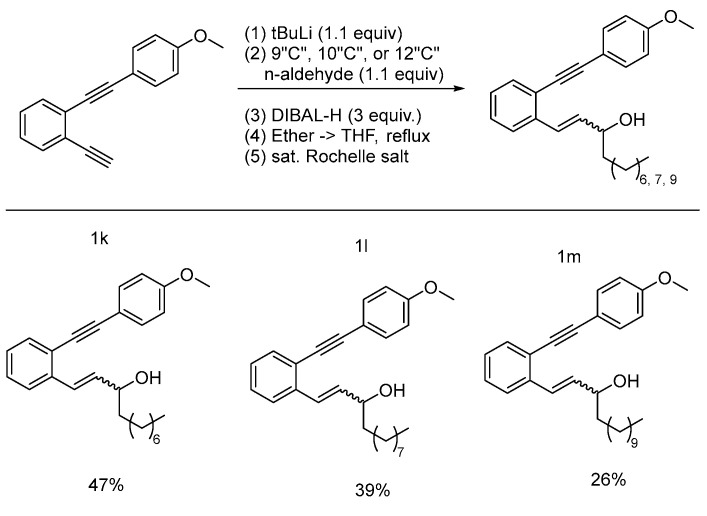
DIBAL-H Route Towards Substituted Enynols.

However, these concerns were, fortunately, not warranted, and this approach works well for the protection of alkyl aldehydes. The acetylide anion [22] addition to the aldehyde can be followed by a one-pot DIBAL-H reduction. The last step is selective and proceeds exclusively at the propargylic alcohol without reduction of the diaryl alkyne moiety, presumably due to the directing effect of the allylic oxygen. This method opens quick access to caged carbonyls with known practical utility (Scheme 8). Specifically, the straight-chain aldehydes nonanal, decanal, and dodecanal are used as fragrance compounds that give the scent of roses, orange rind, and lilac, respectively. The photocaged fragrance aldehydes can be envisioned as photo-controlled release fragrance sources, providing an interesting alternative to the thermal release of excess fragrance compounds typically used in wall outlet units.

### 2.2. Photochemistry

With a streamlined synthetic route established, we synthesized a variety of photocaged aryl ketones and aryl aldehydes to probe the scope of the photouncaging reaction. Although the enynols have a peak absorbance at 300 nm +/− 2 nm, irradiation centered at 300 nm resulted in a complex mixture. However, irradiation at 365 nm (the shoulder of the products’ absorbance) resulted in clean fragmentation of the carbonyl in good to excellent yields.

Guided by these results, we have started a systematic exploration of the photouncaging of an acetophenone family of ketones (Table 1). Compounds were dissolved in benzene and purged with argon for 1 h in a dry round bottom flask, placed inside a Luzchem photobox, and irradiated at 365 nm for 4 h with stirring. When R^1^ = H, the photouncaging proceeds with moderate yields. With the introduction of a methoxy donor, yields increased dramatically, presumably due to lone pair stabilization of the developing radical character in the transition state for the cyclization (Scheme 2, bottom).

We have also explored the photouncaging of aromatic aldehydes (Table 2). In their case, there is an overall moderate decrease in yield due to the relative instability of aldehydes in comparison to their analogous ketones. Again, the yield can be increased, sometimes through methoxy substitution at the arylethynyl terminus. Due to benzaldehyde’s volatility, it was difficult to separate this product from the benzene solvent, though the fulvene byproduct detected suggests photouncaging efficiency in line with the other aldehydes.

**Figure 2:**
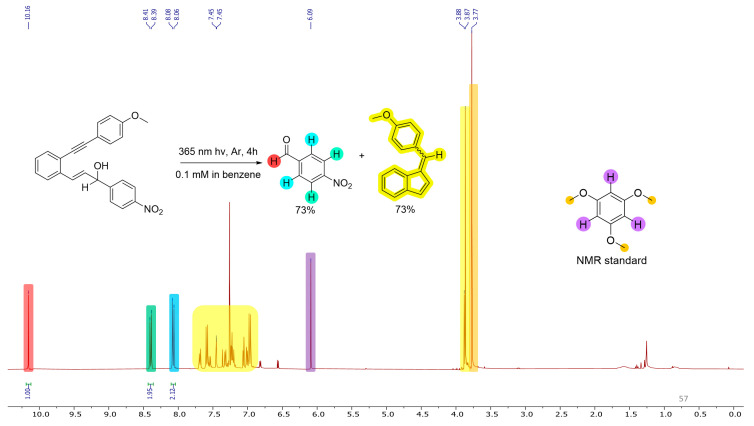
^1^H NMR of the reaction mixture for the photochemical uncaging of p-nitrobenzaldehyde.

While photouncaging of m-nitrobenzaldehyde was accomplished, albeit with a moderate yield, uncaging of o-nitrobenzaldehyde resulted in only a complex mixture of products. We believe that this complexity is derived from byproducts formed following the known ability of aryl nitro groups to abstract ortho benzylic C-Hs in the excited state [23]. The polarity of the excited states and reactive intermediates (e.g., diradical vs. zwitter-ionic [24]) is unlikely to be important in this case as the uncaging of p-nitrobenzaldehyde proceeds very cleanly (Figure 2).

The following examples also demonstrate the ability of the enynols to cage aliphatic aldehydes, in addition to the previously discussed aromatic aldehydes. Photouncaging worked well, providing the fragrance aldehydes in a 51–71% yield (Table 3).

As was the case with benzaldehyde, nonanal’s volatility complicates separation from the benzene solvent. However, the detected fulvene (75%) suggests that the efficiency of photouncaging is similar to that of the other aldehydes.

**Scheme 9:**
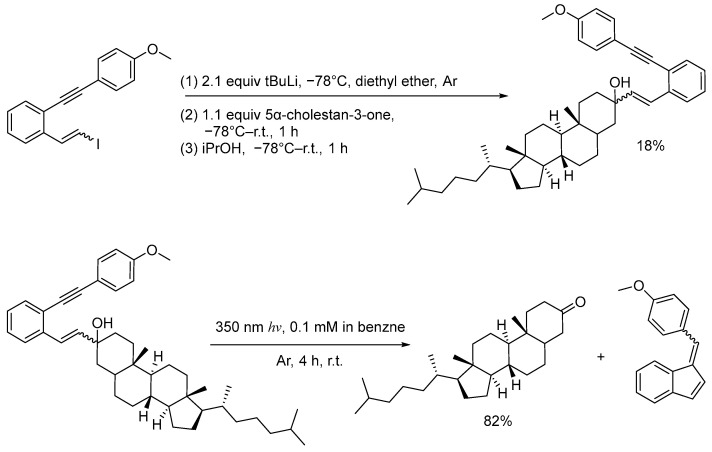
Caging and photouncaging of 5-α-cholesten-3-one.

Finally, to demonstrate the ability of the enynol system to photocage larger and more biologically active compounds, we have prepared the caged steroid 5-α- cholesten-3-one (Scheme 9). Gratifyingly, the photocyclization and fragmentation in this system proceeds cleanly and efficiently, providing 82% of the uncaged ketone.

## 3. Materials and Methods

### 3.1. 2-((4-Methoxyphenyl)ethynyl)benzaldehyde

A suspension of 2-bromobenzaldehyde (16.2 mmol, 3.00 g), PdCl_2_(PPh_3_)_2_ (5 mol%, 0.811 mmol, 569 mg), and CuI (5 mol%, 0.811 mmol, 154 mg) was dissolved in 100 mL of triethylamine and sparged with argon for 30 min in a 250 mL round bottom flask. The 1-ethynyl-4-methoxybenzene (1.1 equiv., 17.8 mmol, 2.36 g) was dissolved in a minimum amount of argon-sparged triethylamine and slowly added dropwise to the suspension, which quickly resulted in the formation of a dark brown precipitant. The solution was allowed to stir for 8 h at room temperature. The reaction mixture was filtered through celite, concentrated on a rotary evaporator under reduced pressure, and purified via flash chromatography on silica gel using ethyl acetate and hexanes as an eluent to afford the aldehyde product in a 91% yield.

### 3.2. 2-(Phenylethynyl)benzaldehyde

A suspension of 2-bromobenzaldehyde (16.2 mmol, 3.00 g), PdCl_2_(PPh_3_)_2_ (5 mol%, 0.811 mmol, 569 mg), and CuI (5 mol%, 0.811 mmol, 154 mg) was dissolved in 100 mL of triethylamine and sparged with argon for 30 min in a 250 mL round bottom flask. The ethynylbenzene (1.1 equiv., 17.8 mmol, 1.82 g) was slowly added dropwise to the suspension, which quickly resulted in the formation of a dark brown precipitant. The solution was allowed to stir for 8 h at room temperature. The reaction mixture was filtered through celite, concentrated on a rotary evaporator under reduced pressure, and purified via flash chromatography on silica gel using ethyl acetate and hexanes as an eluent to afford the aldehyde product in a 95% yield.

### 3.3. Vinyl Iodides

Based on Augé’s modified Takai protocol, a suspension of CrCl_3_ (6 equiv., 86.2 mmol, 13.7 g), Zn powder (3 equiv., 43.1 mmol, 2.82 g), and sodium iodide (5 equiv., 71.8 mmol, 10.8 g) was made by adding them to a dry 500 mL round bottom flask, followed by 144 mL of dry tetrahydrofuran. In a separate dry round bottom flask, the aldehyde (14.4 mmol, 3.39 g) and iodoform (1.5 equiv., 21.6 mmol, 8.48 g) were dissolved in 72 mL of dry tetrahydrofuran. This solution was slowly transferred to the suspension via cannula, which resulted in a steady darkening of the solution to a dark brown. The reaction mixture was stirred for 6 h at room temperature. The reaction mixture was concentrated on silica gel and filtered through a silica gel plug to remove the inorganics. The crude reaction mixture was then purified via flash chromatography using ethyl acetate and hexanes as an eluent, and the vinyl iodide was isolated in 50–54% yield as an E/Z mixture.

### 3.4. Lithium-Halogen Exchange Addition Reactions for Synthesis of Caged Compounds

A 200 mg portion of vinyl iodide was dissolved in 10 mL of dry ether over 4 Å molecular sieves under argon. 1.1 equivalents of ketone or aldehyde were dissolved in 10 mL of dry ether over 4 Å molecular sieves under argon. A 25 mL round-bottom flask with a stir bar was flame-dried and backfilled with argon. The dried vinyl iodide solution was then transferred to the round-bottom flask and cooled to −78 °C. 2.1 Equivalents of tertiary butyllithium in pentane solution were added dropwise, resulting in brief bursts of dark teal coloration with each drop until the solution became fully dark teal at the end of the addition. The aldehyde or ketone solution is then immediately added via fast dropwise addition. After the addition of ketone or aldehyde, the solution steadily changes to a light brown or orange color. The solution was stirred at −78 °C for 1 h and then allowed to warm to room temperature for over 1 h. The solution is then cooled again to −78 °C and quenched by the dropwise addition of isopropanol, which lightens the solution to a transparent yellow. The crude reaction mixture is extracted with a saturated ammonium chloride solution, then washed with deionized water before purification via silica gel flash chromatography using 15–20% ethyl acetate in hexanes as an eluent.

### 3.5. Photouncaging of Enynols

A 16 μmol portion of the enynol is dissolved in 160 mL of benzene and sparged with argon for 1 h in a 250 mL round-bottom flask with a stir bar. The flask is then placed in a Luzchem LZC-4X photoreactor equipped with 14 Hitachi FL8BL-B UVA bulbs centered at 350 nm and stirred under irradiation for 4 h. The solution starts with a very faint yellow color, which changes to a much brighter yellow, indicative of benzofulvene formation. 1,3,5-trimethoxybenzene was added to the reaction mixture as an NMR standard to determine yields, and the solvent was evaporated under reduced pressure.

(**1a**) 4-(2-((4-methoxyphenyl)ethynyl)phenyl)-2-phenylbut-3-en-2-ol

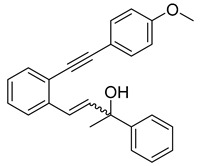

Yellow oil ^1^H NMR (400 MHz, CDCl_3_): δ 7.59–7.53 (m, 3H), 7.50 (dd, *J* = 7.4, 1.5 Hz, 1H), 7.42–7.33 (m, 4H), 7.31–7.18 (m, 4H), 6.88 (d, *J* = 8.8 Hz, 2H), 6.63 (d, *J* = 16.1 Hz, 1H), 3.84 (s, 3H), 1.82 (s, 3H). ^13^ C NMR (101 MHz, CDCl_3_) δ 159.71, 146.49, 138.08, 138.00, 132.99, 132.27, 128.33, 128.11, 127.30, 127.05, 126.46, 125.42, 125.20, 122.49, 115.41, 114.04, 86.57, 74.94, 55.34, 29.85. LCMS (ESI) *m*/*z*: [M + Na] + calcd. for C_25_H_22_O_2_, 377.1512, found: 377.1523.

(**1b**) 2-(4-methoxyphenyl)-4-(2-((4-methoxyphenyl)ethynyl)phenyl)but-3-en-2-ol

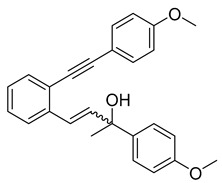

Yellow oil ^1^H NMR (400 MHz, CDCl_3_) δ 7.55 (dd, *J* = 7.8, 1.4 Hz, 1H), 7.52–7.44 (m, 3H), 7.40–7.34 (m, 2H), 7.30–7.23 (m, 1H), 7.22 (d, *J* = 1.5 Hz, 1H), 7.17 (d, *J* = 16.0 Hz, 1H), 6.93–6.83 (m, 4H), 6.62 (d, *J* = 16.1 Hz, 1H), 3.83 (s, 3H), 3.79 (s, 3H), 1.79 (s, 3H). ^13^C NMR (101 MHz, CDCl_3_) δ 159.96, 158.90, 138.90, 138.66, 138.34, 133.25, 132.53, 128.39, 127.52, 127.05, 126.52, 125.44, 122.73, 115.67, 114.33, 113.88, 94.61, 86.91, 74.92, 55.57, 55.51, 30.09. LCMS (ESI) *m*/*z*: [M + Na] + calcd. for C_26_H_24_O_3_, 407.1618, found: 407.1619.

(**1c**) (2-hydroxy-4-(2-((4-methoxyphenyl)ethynyl)phenyl)but-3-en-2-yl)benzonitrile

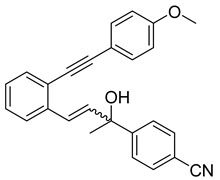

Yellow oil ^1^H NMR (400 MHz, CDCl_3_) δ 7.69–7.62 (m, 2H), 7.59 (d, *J* = 8.7 Hz, 2H), 7.54–7.46 (m, 2H), 7.33 (d, *J* = 8.8 Hz, 2H), 7.30–7.17 (m, 2H), 7.13 (d, *J* = 16.2 Hz, 1H), 6.90 (d, *J* = 8.9 Hz, 2H), 6.55 (d, *J* = 16.1 Hz, 1H), 3.84 (s, 3H), 1.79 (s, 3H). ^13^C NMR (151 MHz, CDCl_3_) δ 160.19, 152.10, 137.71, 137.04, 133.15, 132.67, 132.40, 128.64, 128.49, 128.10, 128.01, 125.50, 122.95, 119.19, 115.39, 114.49, 111.09, 94.84, 86.61, 75.11, 55.66, 30.20. LCMS (ESI) *m*/*z*: [M + Na]+ calcd. for C_26_H_21_NO_2_, 402.1465, found: 402.1483.

(**1d**) 4-(2-((4-methoxyphenyl)ethynyl)phenyl)-2-(4-nitrophenyl)but-3-en-2-ol

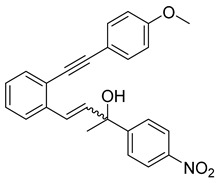

Brown oil ^1^H NMR (400 MHz, CDCl_3_) δ 8.16 (d, *J* = 8.8 Hz, 2H), 7.71 (d, *J* = 8.8 Hz, 2H), 7.50 (ddd, *J* = 9.4, 7.4, 1.6 Hz, 2H), 7.40 (dd, *J* = 10.0, 7.9 Hz, 1H), 7.34–7.19 (m, 4H), 7.14 (d, *J* = 16.2 Hz, 1H), 6.86 (d, *J* = 8.7 Hz, 2H), 6.58 (d, *J* = 16.2 Hz, 1H), 3.84 (s, 3H), 1.82 (s, 3H). LCMS (ESI) *m*/*z*: [M + Na] + calcd. for C_25_H_21_NO_4_, 422.1363, found: 422.1384.

(**1e**) 3-(2-((4-methoxyphenyl)ethynyl)phenyl)-1-(2-nitrophenyl)prop-2-en-1-ol

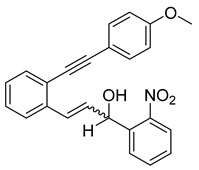

Brown oil ^1^H NMR (400 MHz, CDCl_3_) δ 7.93 (dd, *J* = 8.2, 1.3 Hz, 1H), 7.87 (dd, *J* = 7.9, 1.4 Hz, 1H), 7.63 (td, *J* = 7.6, 1.4 Hz, 1H), 7.54–7.40 (m, 6H), 7.32 (dd, *J* = 16.1, 1.4 Hz, 1H), 7.28–7.18 (m, 3H), 6.91–6.86 (m, 2H), 6.55 (dd, *J* = 16.0, 5.8 Hz, 1H), 6.08–6.02 (m, 1H), 3.84 (s, 3H). LCMS (ESI) *m*/*z*: [M + Na] + calcd. for C_24_H_19_NO_4_, 408.1206, found: 408.1225.

(**1f**) 3-(2-((4-methoxyphenyl)ethynyl)phenyl)-1-phenylprop-2-en-1-ol

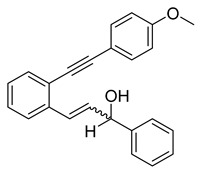

Yellow oil ^1^H NMR (400 MHz, CDCl_3_) δ 7.63–7.17 (m, 20H), 7.00 (d, *J* = 11.4 Hz, 1H), 6.89 (dd, *J* = 8.9, 2.3 Hz, 3H), 6.53 (dd, *J* = 15.9, 6.4 Hz, 1H), 5.44 (d, *J* = 6.5 Hz, 1H), 3.83 (s, 3H), 3.82 (s, 1H). ^13^C NMR (101 MHz, CDCl_3_) δ 159.99, 143.26, 142.97, 138.49, 138.05, 134.44, 133.70, 133.35, 133.30, 132.67, 132.44, 130.26, 129.23, 129.17, 128.89, 128.87, 128.40, 128.12, 128.02, 127.91, 127.70, 126.76, 126.56, 125.63, 122.70, 115.66, 114.36, 114.33, 94.59, 94.56, 87.20, 86.86, 75.51, 70.43, 55.60. LCMS (ESI) *m*/*z*: [M + Na] + calcd. for C_24_H_20_O_2_, 363.1356, found: 363.1363.

(**1g**) 1-(4-methoxyphenyl)-3-(2-((4-methoxyphenyl)ethynyl)phenyl)prop-2-en-1-ol

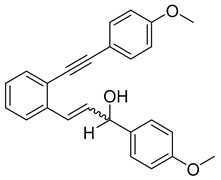

Yellow oil ^1^H NMR (400 MHz, CDCl_3_) δ 7.54 (dd, *J* = 7.7, 1.4 Hz, 1H), 7.52–7.48 (m, 1H), 7.43–7.36 (m, 4H), 7.30–7.25 (m, 2H), 7.25–7.16 (m, 3H), 6.93–6.86 (m, 5H), 6.53 (dd, *J* = 15.9, 6.2 Hz, 1H), 5.40 (d, *J* = 6.2 Hz, 1H), 3.83 (s, 3H), 3.80 (s, 3H), 2.26 (s, 1H). LCMS (ESI) *m*/*z*: [M+Na] + calcd. for C_25_H_22_O_3_, 393.1461, found: 393.1475.

(**1h**) 4-(1-hydroxy-3-(2-((4-methoxyphenyl)ethynyl)phenyl)allyl)benzonitrile

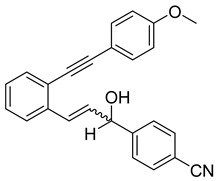

Yellow oil ^1^H NMR (400 MHz, CDCl_3_) δ 7.66–7.62 (m, 2H), 7.60–7.56 (m, 2H), 7.51 (dt, *J* = 7.5, 1.9 Hz, 2H), 7.41–7.36 (m, 2H), 7.30–7.22 (m, 3H), 7.19 (dd, *J* = 16.0, 1.2 Hz, 1H), 6.93–6.88 (m, 2H), 6.43 (dd, *J* = 15.9, 6.9 Hz, 1H), 5.50 (d, *J* = 6.9 Hz, 1H), 3.85 (s, 3H), 2.25 (s, 1H). ^13^C NMR (101 MHz, CDCl_3_) δ 160.21, 148.01, 137.40, 133.25, 132.87, 132.71, 132.34, 132.13, 130.76, 128.55, 128.24, 127.38, 125.67, 122.95, 115.42, 114.47, 111.73, 94.80, 86.56, 74.96, 55.71. LCMS (ESI) *m*/*z*: [M + Na] + calcd. for C_25_H_19_NO_2_, 388.1308, found: 388.1321.

(**1i**) 3-(2-((4-methoxyphenyl)ethynyl)phenyl)-1-(4-nitrophenyl)prop-2-en-1-ol

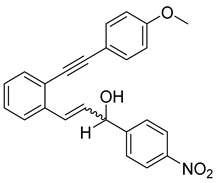

Brown oil ^1^H NMR (400 MHz, CDCl_3_) δ 8.20 (d, *J* = 8.7 Hz, 2H), 7.63 (d, *J* = 8.7 Hz, 2H), 7.51 (d, *J* = 7.0 Hz, 2H), 7.41–7.35 (m, 2H), 7.26 (qd, *J* = 7.0, 1.7 Hz, 3H), 7.23–7.16 (m, 1H), 6.91–6.85 (m, 2H), 6.45 (dd, *J* = 15.9, 6.9 Hz, 1H), 5.55 (d, *J* = 6.9 Hz, 1H), 3.85 (s, 3H). ^13^C NMR (101 MHz, CDCl_3_) δ 160.05, 149.82, 137.20, 133.20, 133.05, 132.71, 132.09, 130.76, 128.51, 128.39, 128.11, 127.31, 127.07, 125.54, 123.94, 123.85, 122.80, 115.21, 114.34, 114.27, 94.66, 86.38, 74.63, 55.53. LCMS (ESI) *m*/*z*: [M + Na] + calcd. for C_24_H_19_NO_4_, 408.1206, found: 408.1227.

(**1j**) 3-(2-((4-methoxyphenyl)ethynyl)phenyl)-1-(3-nitrophenyl)prop-2-en-1-ol

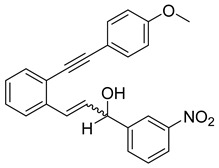

Brown oil ^1^H NMR (400 MHz, CDCl_3_) δ 8.36 (t, *J* = 2.1 Hz, 1H), 8.17–8.10 (m, 1H), 7.79 (dt, *J* = 7.8, 1.0 Hz, 1H), 7.56–7.48 (m, 3H), 7.45–7.39 (m, 2H), 7.31–7.21 (m, 4H), 6.92–6.86 (m, 2H), 6.47 (dd, *J* = 15.8, 7.0 Hz, 1H), 5.56 (d, *J* = 6.1 Hz, 1H), 3.85 (s, 3H), 2.29 (s, 1H). ^13^C NMR (101 MHz, CDCl_3_) δ 160.15, 148.73, 145.07, 137.39, 133.34, 133.24, 132.83, 132.81, 132.41, 130.72, 129.75, 128.52, 128.19, 125.73, 122.91, 122.89, 121.60, 115.43, 114.45, 114.42, 94.83, 86.59, 74.66, 55.66. LCMS (ESI) *m*/*z*: [M + Na] + calcd. for C_24_H_19_NO_4_, 408.1206, found: 408.1228.

(**2c**) 4-(2-hydroxy-4-(2-(phenylethynyl)phenyl)but-3-en-2-yl)benzonitrile

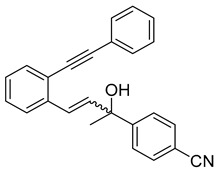

Yellow oil ^1^H NMR (400 MHz, CDCl_3_) δ 7.70–7.64 (m, 2H), 7.64–7.58 (m, 2H), 7.57–7.49 (m, 2H), 7.43–7.35 (m, 5H), 7.34–7.21 (m, 3H), 7.16 (d, *J* = 16.1 Hz, 1H), 6.57 (d, *J* = 16.1 Hz, 1H), 2.14 (s, 1H), 1.81 (s, 3H). ^13^C NMR (101 MHz, CDCl_3_) δ 152.03, 137.92, 137.20, 132.84, 132.39, 131.67, 128.87, 128.83, 128.79, 128.63, 128.01, 127.84, 126.63, 125.51, 123.29, 122.54, 119.16, 111.04, 94.72, 87.87, 75.07, 30.13.

(**2d**) 2-(4-nitrophenyl)-4-(2-(phenylethynyl)phenyl)but-3-en-2-ol

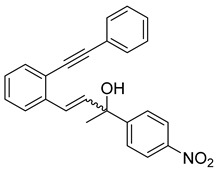

Brown oil ^1^H NMR (400 MHz, CDCl_3_) δ 8.23–8.12 (m, 2H), 7.78–7.67 (m, 2H), 7.52 (ddd, *J* = 7.0, 4.8, 1.4 Hz, 2H), 7.42–7.37 (m, 2H), 7.37–7.31 (m, 3H), 7.30–7.21 (m, 2H), 7.16 (d, *J* = 16.3 Hz, 1H), 6.58 (d, *J* = 16.1 Hz, 1H), 2.29 (s, 1H), 1.83 (s, 3H). ^13^C NMR (101 MHz, CDCl_3_) δ 154.02, 147.23, 137.89, 137.12, 132.87, 131.64, 128.89, 128.86, 128.76, 128.08, 126.82, 125.55, 123.77, 123.28, 122.61, 94.77, 87.86, 75.15, 30.26.

(**2h**) 4-(1-hydroxy-3-(2-(phenylethynyl)phenyl)allyl)benzonitrile

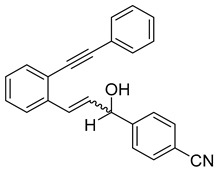

Yellow oil ^1^H NMR (400 MHz, CDCl_3_) δ 7.66–7.61 (m, 2H), 7.61–7.55 (m, 2H), 7.55–7.50 (m, 2H), 7.50–7.43 (m, 3H), 7.40–7.36 (m, 3H), 7.32–7.24 (m, 3H), 7.24–7.17 (m, 1H), 6.44 (dd, *J* = 15.8, 6.9 Hz, 1H), 5.50 (d, *J* = 7.0 Hz, 1H).

(**2i**) 1-(4-nitrophenyl)-3-(2-(phenylethynyl)phenyl)prop-2-en-1-ol

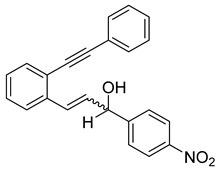

Brown oil ^1^H NMR (400 MHz, CDCl_3_) δ 8.27–8.18 (m, 2H), 7.68–7.63 (m, 2H), 7.56–7.51 (m, 2H), 7.48–7.44 (m, 2H), 7.38–7.34 (m, 3H), 7.33–7.27 (m, 3H), 7.26–7.19 (m, 2H), 6.46 (dd, *J* = 15.9, 7.0 Hz, 1H), 5.57 (d, *J* = 7.0 Hz, 1H), 2.22 (s, 1H).

(**1n**) (8R,9S,10S,13R,14S,17R)-3-(2-((4-methoxyphenyl)ethynyl)styryl)-10,13-dimethyl-17-((R)-6-methylheptan-2-yl)hexadecahydro-1H-cyclopenta[a]phenanthren-3-ol

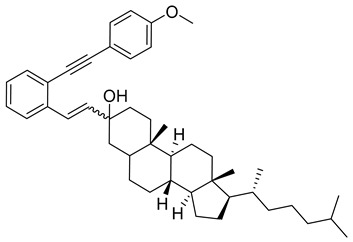

Yellow oil ^1^H NMR (600 MHz, Chloroform-*d*) δ 7.57–7.53 (m, 1H), 7.51 (dd, *J* = 7.6, 1.4 Hz, 1H), 7.49–7.45 (m, 2H), 7.28 (td, *J* = 7.7, 1.4 Hz, 1H), 7.24 (d, *J* = 16.1 Hz, 1H), 7.22–7.19 (m, 1H), 6.91–6.84 (m, 2H), 6.49 (d, *J* = 16.3 Hz, 1H), 3.81 (s, 3H), 1.92 (dd, *J* = 12.0, 3.5 Hz, 2H), 1.82 (dd, *J* = 13.4, 3.7 Hz, 2H), 1.72–1.65 (m, 2H), 1.60–1.55 (m, 2H), 1.54–1.49 (m, 2H), 1.40 (d, *J* = 5.0 Hz, 1H), 1.37–1.32 (m, 4H), 1.31–1.29 (m, 1H), 1.27 (q, *J* = 2.1 Hz, 1H), 1.21 (ddd, *J* = 19.0, 10.9, 3.8 Hz, 5H), 1.17–1.09 (m, 4H), 1.05–0.97 (m, 4H), 0.91–0.89 (m, 4H), 0.88 (d, *J* = 2.8 Hz, 3H), 0.87 (d, *J* = 2.7 Hz, 3H), 0.86 (s, 3H), 0.80–0.76 (m, 1H), 0.64 (s, 3H), 0.59 (d, *J* = 2.5 Hz, 1H). ^13^C NMR (151 MHz, CDCl_3_) δ 160.02, 138.78, 137.18, 133.44, 133.40, 133.23, 132.73, 128.46, 127.76, 127.47, 125.68, 122.61, 115.75, 114.36, 94.63, 86.95, 73.11, 56.66, 56.63, 55.54, 54.54, 43.84, 42.89, 42.14, 40.31, 39.83, 36.97, 36.66, 36.50, 36.14, 36.11, 35.78, 35.75, 35.65, 34.99, 34.79, 32.14, 31.90, 29.03, 28.55, 28.31, 26.75, 25.60, 25.01, 24.49, 24.22, 24.19, 23.72, 23.13, 22.97, 22.88, 21.53, 21.02, 18.97, 14.43, 12.46, 12.37, 12.34, 11.74. LCMS (ESI) *m*/*z*: [M + Na] + calcd. for C_44_H_60_O_2_, 643.4485, found: 643.4482.

## 4. Conclusions

In conclusion, we have shown that photochemical C1–C5 cycloaromatization of enynols can be used to uncage ketones and aldehydes. We have also developed two synthetic routes to access ortho enynols with one-step derivatization of the allylic position from a common precursor. From them, a range of aromatic and aliphatic ketones and aldehydes can be caged and photouncaged in good to excellent yields using direct UVA irradiation. The photouncaging process is tunable, as illustrated by an increase in photouncaging yields upon donor substitution at the alkyne terminus. Our continuing work involves tuning the enynol system to shift its absorbance to longer wavelengths for even milder activation.

## Figures and Tables

**Table 1:**
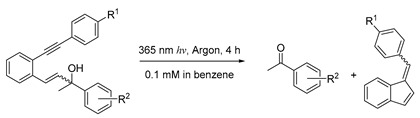
Photouncaging of Aromatic Ketones.

R^1^	R^2^	Ketone	Fulvene
H	p-CN	54%	62%
H	p-NO_2_	66%	62%
OCH_3_	p-OCH_3_	84%	87%
OCH_3_	H	76%	79%
OCH_3_	p-CN	95%	85%
OCH_3_	p-NO_2_	92%	41%

**Table 2:**
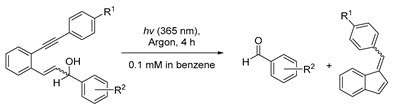
Photouncaging of Aromatic Aldehydes.

R^1^	R^2^	Aldehyde	Fulvene
H	p-CN	76%	98%
H	p-NO_2_	44%	48%
OCH_3_	p-OCH_3_	82%	85%
OCH_3_	H	7%	70%
OCH_3_	p-CN	75%	75%
OCH_3_	p-NO_2_	73%	73%
OCH_3_	m-NO_2_	57%	72%
OCH_3_	o-NO_2_	0%	0%

**Table 3:**
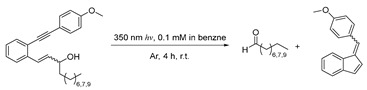
Photouncaging of Fragrance Aldehydes.

Compound	Aldehyde	Fulvene
Nonanal	51%	75%
Decanal	71%	84%
Dodecanal	68%	68%

## Data Availability

All data related to this research is reported in the previous section, Supplementary Materials.

## Supplementary Materials

The following supporting information can be downloaded at: https://www.mdpi.com/article/10.3390/molecules28155704/s1, I: Materials and Methods; II: General Procedures; III: Spectral Data for the new compounds.
