# Supplementary material for: Photochemical Uncaging of Aldehydes and Ketones via Photocyclization/Fragmentation Cascades of Enyne Alcohols: An Unusual Application for a Cycloaromatization Process
Source: Molecules. 2023 Jul 28;28(15):5704. doi: 10.3390/molecules28155704 (PMC10420836; doi:10.3390/molecules28155704)
Supplement: Supplementary file 1 [file molecules-28-05704-s001.zip › molecules-2499205-supplementary.pdf]

Supporting Information

Photochemical Uncaging of Aldehydes and Ketones via Photocyclization/Fragmentation Cascades of Enyne Alcohols: An Unusual Application for a Cycloaromatization Process

Adam T. Campbell,[a] Nikolas R. Dos Santos[a] and Igor Alabugin*[a]

[a] Department of Chemistry and Biochemistry, Florida State University, Tallahassee, FL 32306, USA, e-mail: alabugin@chem.fsu.edu

I. Materials and Methods S1

II. General Procedures S1

III. Spectral Data for The New Compounds.. S4

IV. References...S51

I. Materials and Methods

Tetrahydrofuran (THF) was purchased from Sigma Aldrich and collected through a Glass contour solvent purification system by SG Water USA, LLC. All other chemicals were purchased through Sigma Aldrich and used as received. The NMR spectra were measured on a Bruker spectrometer at 400/600 MHz for ^{1}H and 101/151 MHz for ^{13}C and referenced to the resonances of $CDCl_3$. All irradiation was performed in a Luzchem LZC-4X photoreactor equipped with 14 Hitachi FL8BL-B UVA bulbs centered at 350 nm. The MS full scan spectra with accurate mass determination were obtained by Agilent 6230 time-of-flight TOF mass spectrometer. Ionization condition conditions: drying gas (N2) flow rate, 10.0 L/min; dry temperature, 350 °C; Nebulizer, 40 psi; Fragmentor, 175 V; Capillary voltage, 2000 V; Skimmer potential, 75 V; Hexapole RF, 250 V. All the operations, acquisition and analysis were controlled by Mass Hunter Workstation. UV-VIS spectroscopy was recorded on an Agilent Cary 60 UV-VIS at concentrations of 50 μM.

II. General Procedures:

Sonogashira cross coupling to prepare benzaldehydes using p-OMephenylacetylene.

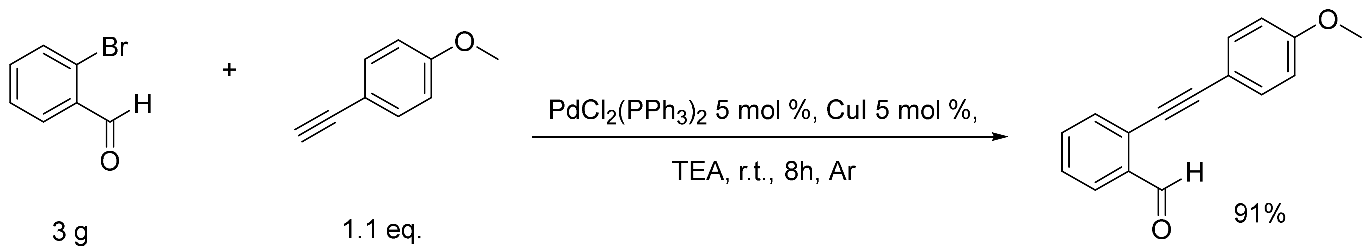

A suspension of 2-bromobenzaldehyde (16.2 mmol, 3.00 g), $PdCl_2(PPh_3)_2$ (5 mol %, 0.811 mmol, 569 mg), CuI (5 mol %, 0.811 mmol, 154 mg), was dissolved in 100 mL of triethylamine and sparged with argon for 30 minutes in a 250 mL round bottom flask. The 1-ethynyl-4-methoxybenzene (1.1 equiv., 17.8 mmol, 2.36 g) was dissolved in a minimum amount of argon-sparged triethylamine, added slowly dropwise to the suspension, which quickly resulted in the formation of a dark brown precipitant. The solution was allowed to stir for 8 hours at room temperature. The reaction mixture was filtered through celite, concentrated on a rotary evaporator under reduced pressure, and purified via flash chromatography on silica gel using ethyl acetate and hexanes as an eluent to afford the aldehyde product in a 91% yield.

Sonogashira cross coupling to prepare benzaldehydes using phenylacetylene.

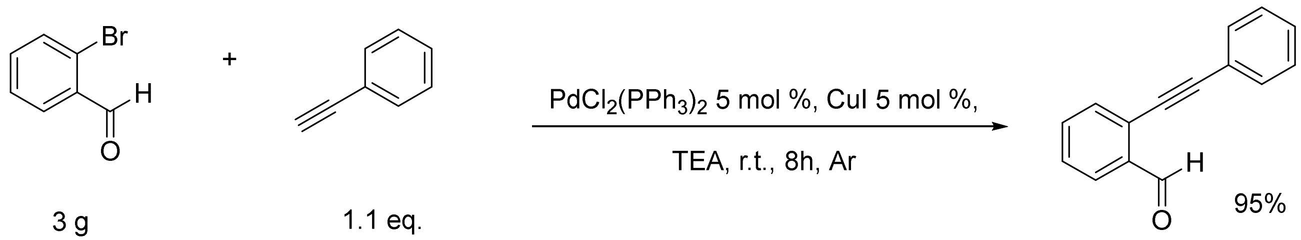

A suspension of 2-bromobenzaldehyde (16.2 mmol, 3.00 g), PdCl2(PPh3)2 (5 mol %, 0.811 mmol, 569 mg), CuI (5 mol %, 0.811 mmol, 154 mg), was dissolved in 100 mL of triethylamine and sparged with argon for 30 minutes in a 250 mL round bottom flask. The ethynylbenzene (1.1 equiv., 17.8 mmol, 1.82 g) was added slowly dropwise to the suspension which quickly resulted in the formation of a dark brown precipitant. The solution was allowed to stir for 8 hours at room temperature. The reaction mixture was filtered through celite, concentrated on a rotary evaporator under reduced pressure, and purified via flash chromatography on silica gel using ethyl acetate and hexanes as an eluent to afford the aldehyde product in a 95% yield.

Takai reaction to prepare vinyl iodides.

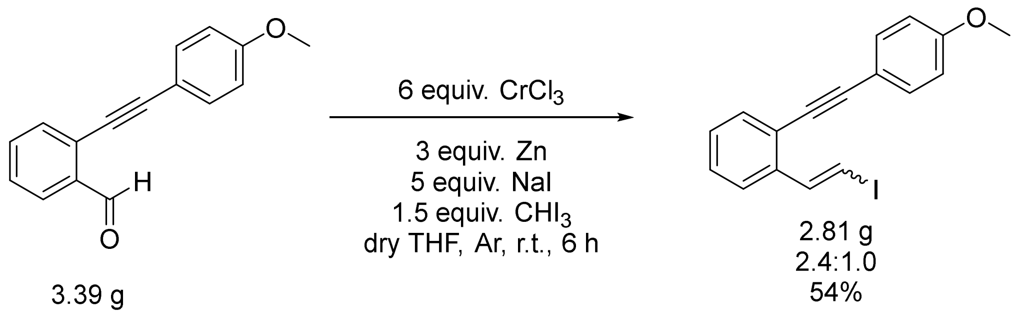

Based on Augé's[1] modified Takai protocol, a suspension of $CrCl_3$ (6 equiv, 86.2 mmol, 13.7 g), Zn powder (3 equiv., 43.1 mmol, 2.82 g), and sodium iodide (5 equiv., 71.8 mmol, 10.8 g) was made by adding them to a dry 500 mL round bottom flask followed by 144 mL of dry tetrahydrofuran. In a separate dry round bottom flask, the aldehyde (14.4 mmol, 3.39 g) and iodoform (1.5 equiv., 21.6 mmol, 8.48 g) were dissolved in 72 mL of dry tetrahydrofuran. This solution was transferred to the suspension slowly via cannula which resulted in a steady darkening of the solution to a dark brown. The reaction mixture was stirred for 6 hours at room temperature. The reaction mixture was concentrated onto silica gel, and filtered through a silica gel plug to remove the inorganics. The crude reaction mixture was then purified via flash chromatography using ethyl acetate and hexanes as an eluent and the vinyl iodide was isolated in 50-54% yield as an E/Z mixture.

Lithium-halogen exchange addition reactions for synthesis of caged compounds.

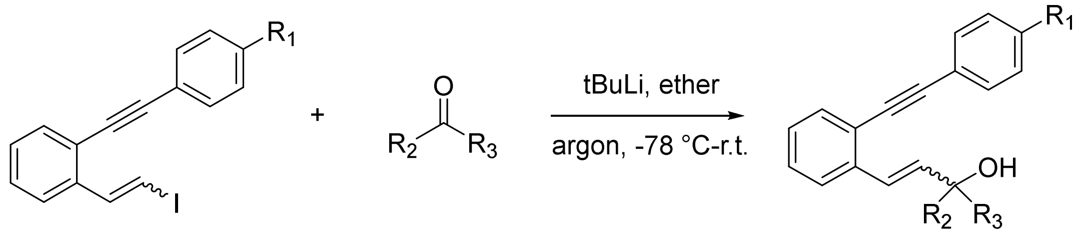

A 200 mg portion of vinyl iodide was dissolved in 10 mL of dry ether over 4 Å molecular sieves under argon. 1.1 equivalents of ketone or aldehyde was dissolved in 10 mL of dry ether over 4 Å molecular sieves under argon. A 25 mL round bottom flask with stir bar was flame-dried and backfilled with argon. The dried vinyl iodide solution was then transferred to the round bottom flask, and cooled to -78 °C. 2.1 Equivalents of tertiary butyllithium in pentane solution was added dropwise, resulting in brief bursts of dark teal coloration with each drop, until the solution became fully dark teal at the end of the addition. The aldehyde or ketone solution is then immediately added via fast dropwise addition. After the addition of ketone or aldehyde, the solution steadily changes to a light brown or orange color. The solution was stirred at -78 °C for 1 hour and then allowed to warm to room temperature over 1 hour. The solution is then cooled again to -78 °C and quenched by dropwise addition of isopropanol, which lightens the solution to a transparent yellow. The crude reaction mixture is extracted with saturated ammonium chloride solution, then washed with deionized water before purification via silica gel flash chromatography using 15-20% ethyl acetate in hexanes as an eluent.

Photouncaging of enynols.

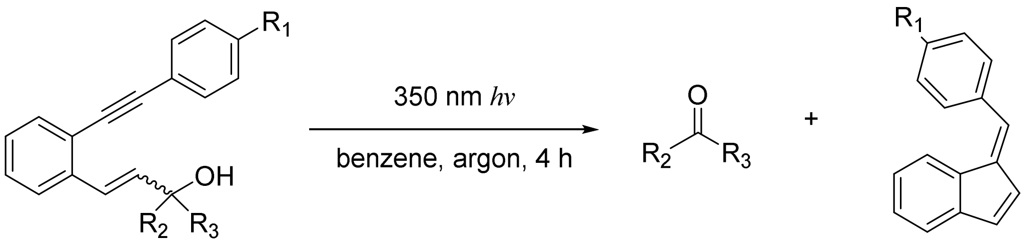

A 16 µmol portion of the enynol is dissolved in 160 mL of benzene and sparged with argon for 1 h in a 250 mL round bottom flask with stir bar. The flask is then placed in a Luzchem LZC-4X photoreactor equipped with 14 Hitachi FL8BL-B UVA bulbs centered at 350 nm and stirred under irradiation for 4 hours. The solution starts with a very faint yellow color, which changes to a much brighter yellow indicative of benzofulvene formation. 1,3,5-trimethoxybenzene was added to the reaction mixture an NMR standard to determine yields and the solvent was evaporated under reduced pressure.

III. Spectral Data for the new compounds:

Combination of singlet at 2.05ppm, quartet at 4.12 ppm, and triplet at 1.26 ppm in various ^{1}H NMR comes from EtOAc in sample.

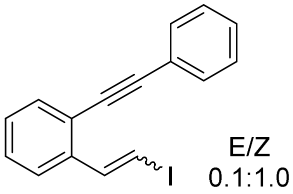

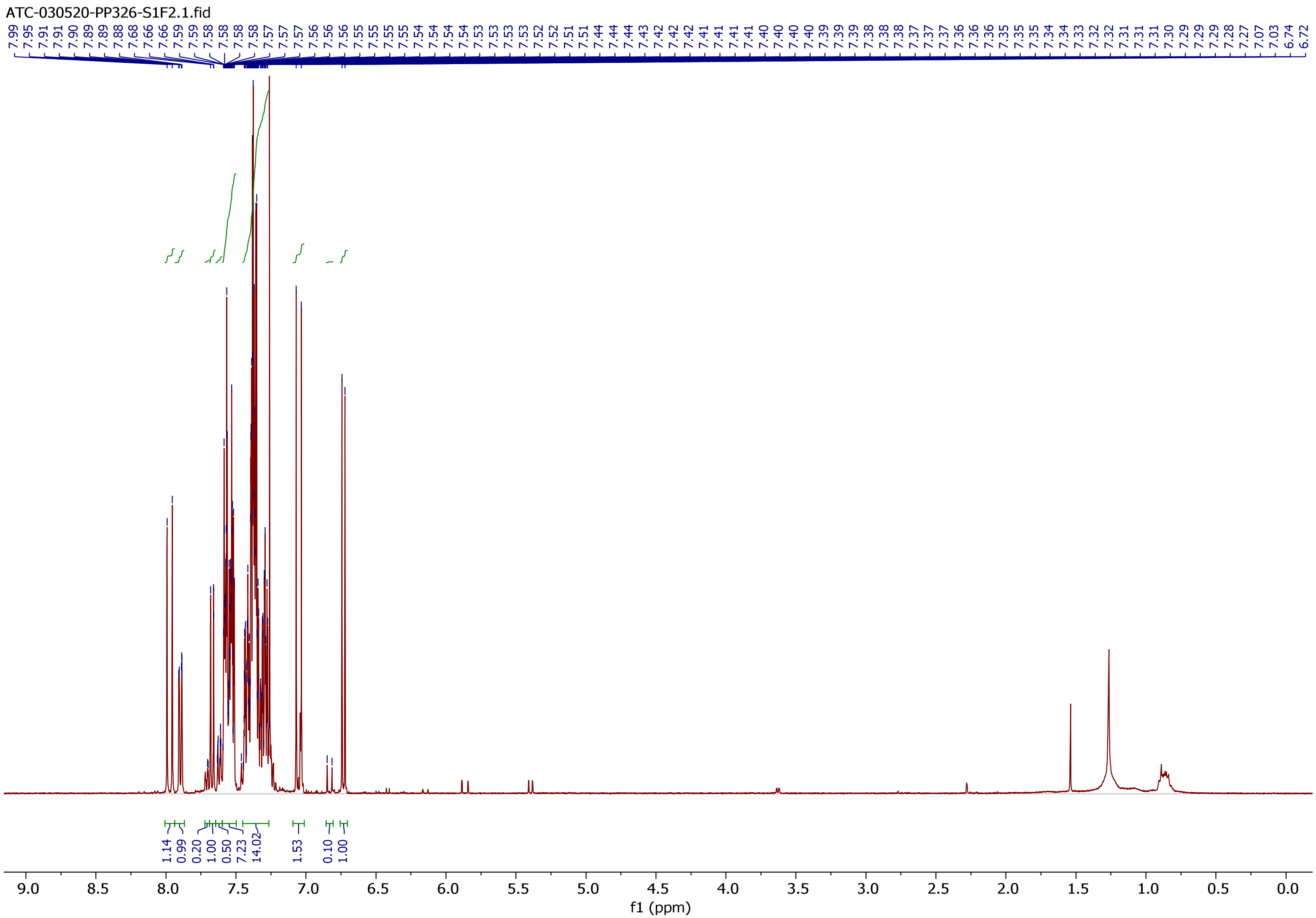
ATC-030520-PP326-S1F2.1.fid
7.99 7.95 7.91 7.91 7.90 7.89 7.89 7.88 7.68 7.66 7.59 7.59 7.58 7.58 7.58 7.58 7.57 7.57 7.56 7.56 7.56 7.55 7.55 7.55 7.54 7.54 7.54 7.53 7.53 7.53 7.52 7.51 7.51 7.44 7.44 7.43 7.42 7.42 7.41 7.41 7.41 7.40 7.40 7.40 7.39 7.39 7.38 7.38 7.37 7.37 7.36 7.36 7.35 7.35 7.34 7.34 7.33 7.32 7.32 7.31 7.31 7.30 7.29 7.29 7.28 7.27 7.07 7.03 6.74 6.72
1.14 0.99 0.20 1.00 0.50 7.23 14.02 1.53 0.10 1.00
9.0 8.5 8.0 7.5 7.0 6.5 6.0 5.5 5.0 4.5 4.0 3.5 3.0 2.5 2.0 1.5 1.0 0.5 0.0
f1 (ppm)

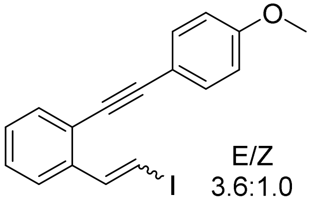

E/Z
3.6:1.0

ATC-011719-238-Yellow.1.fid

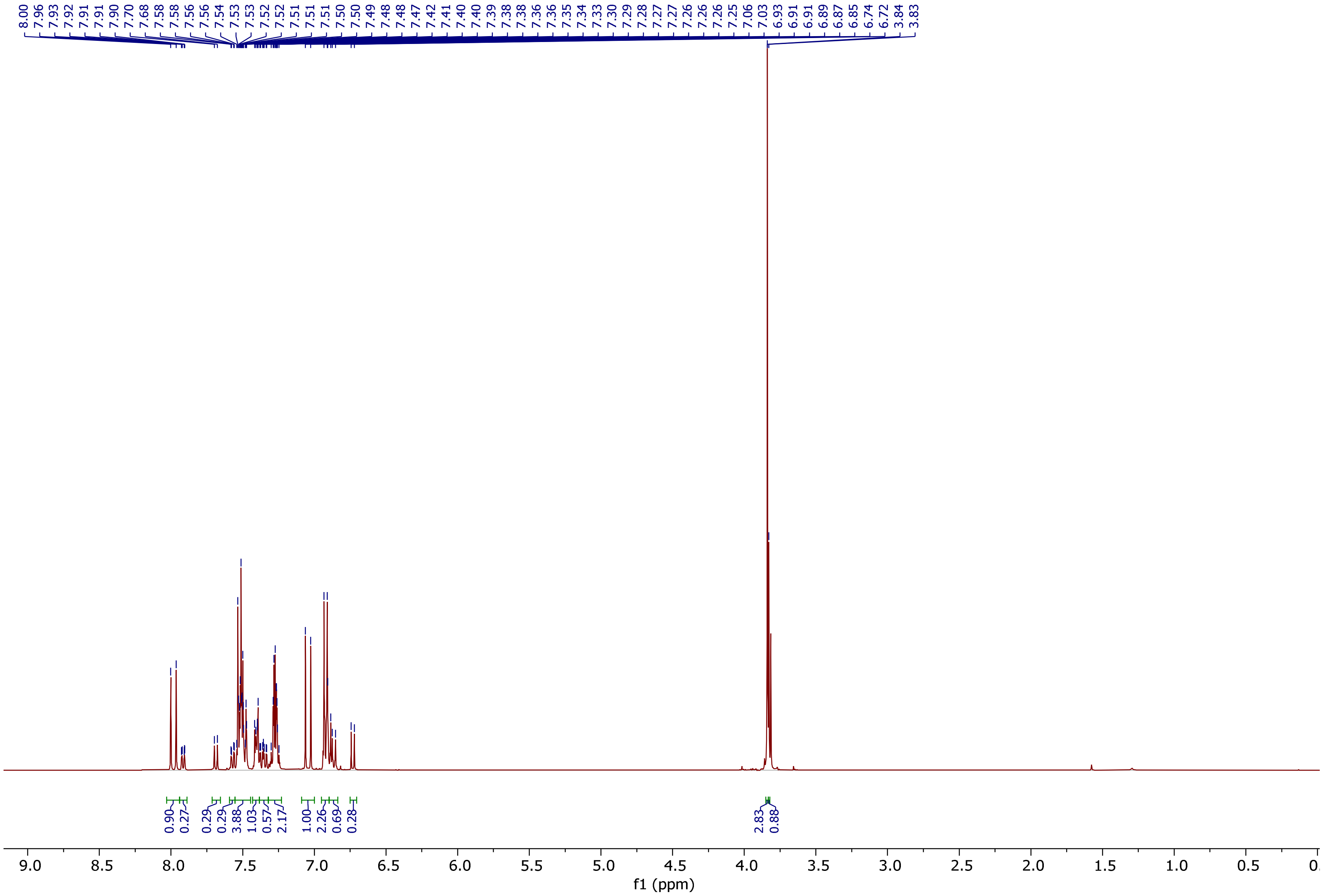

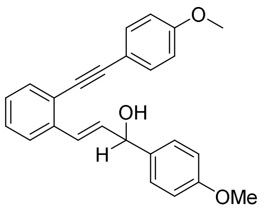

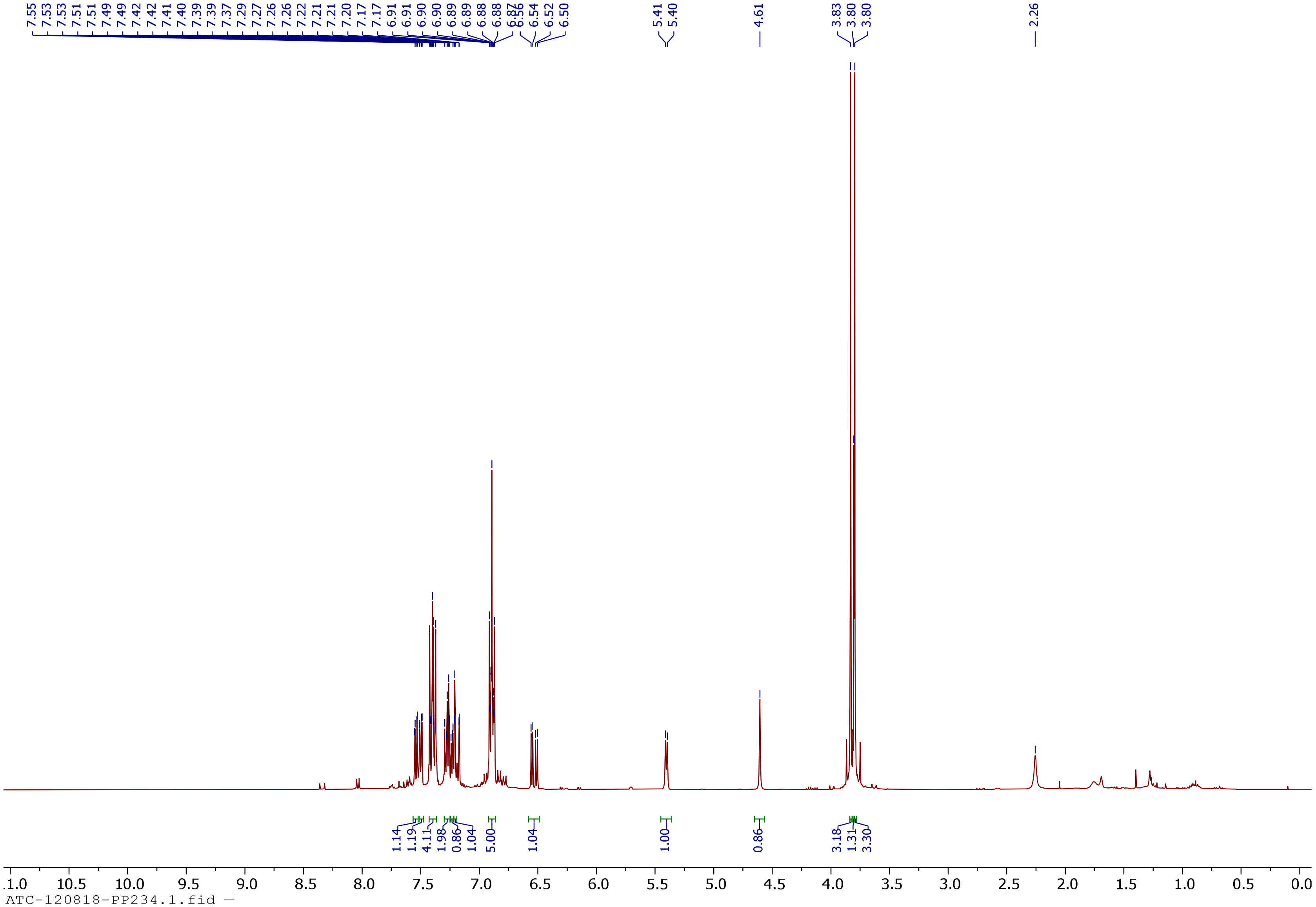

ATC-120818-PP234.1.fid —

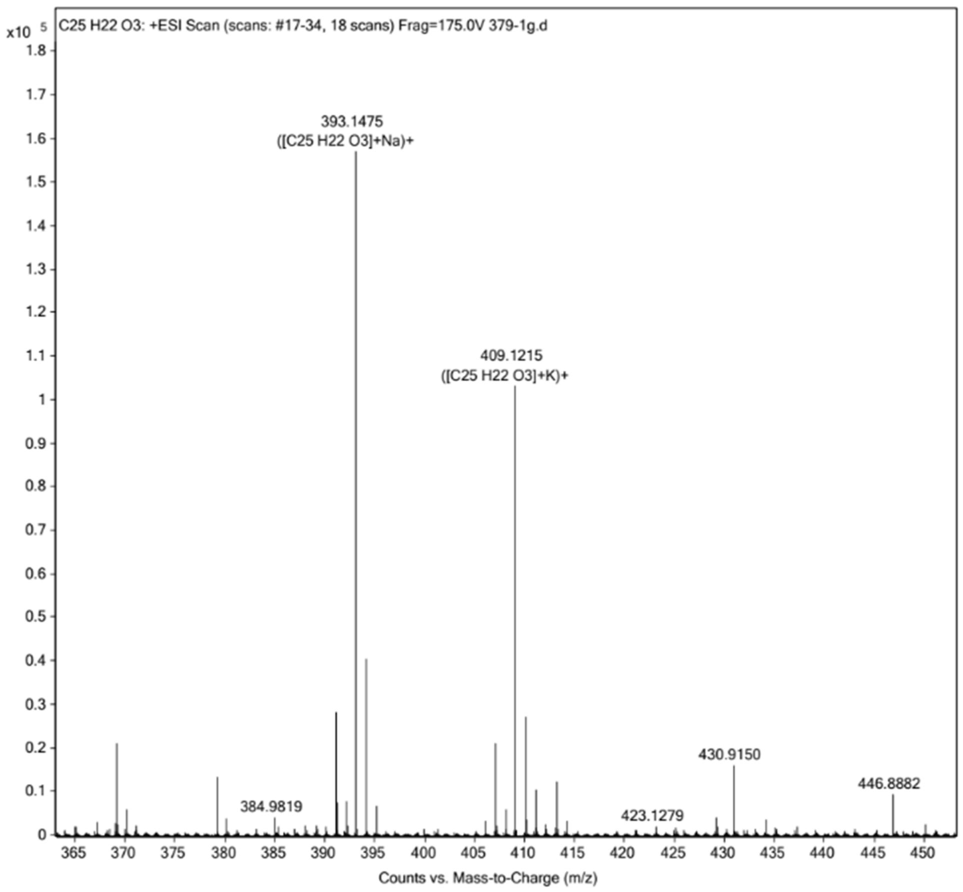

C25 H22 O3: +ESI Scan (scans: #17-34, 18 scans) Frag=175.0V 379-1g.d
x10 5
393.1475
([C25 H22 O3]+Na)+
409.1215
([C25 H22 O3]+K)+
384.9819
423.1279
430.9150
446.8882
Counts vs. Mass-to-Charge (m/z)

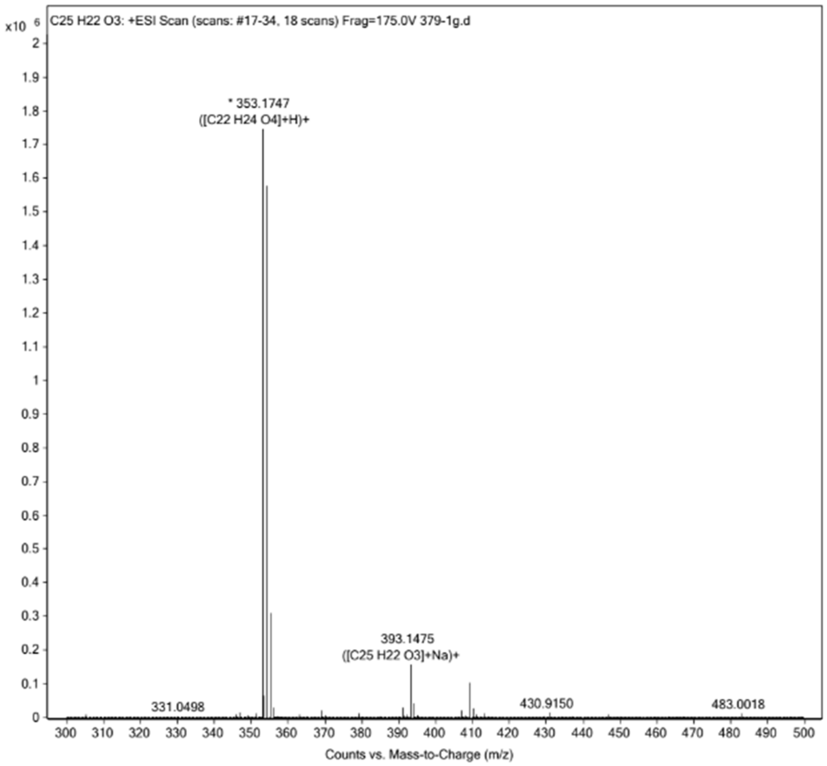
C25 H22 O3: +ESI Scan (scans: #17-34, 18 scans) Frag=175.0V 379-1g.d
x10 6
* 353.1747
([C22 H24 O4]+H)+
393.1475
([C25 H22 O3]+Na)+
331.0498
430.9150
483.0018
Counts vs. Mass-to-Charge (m/z)

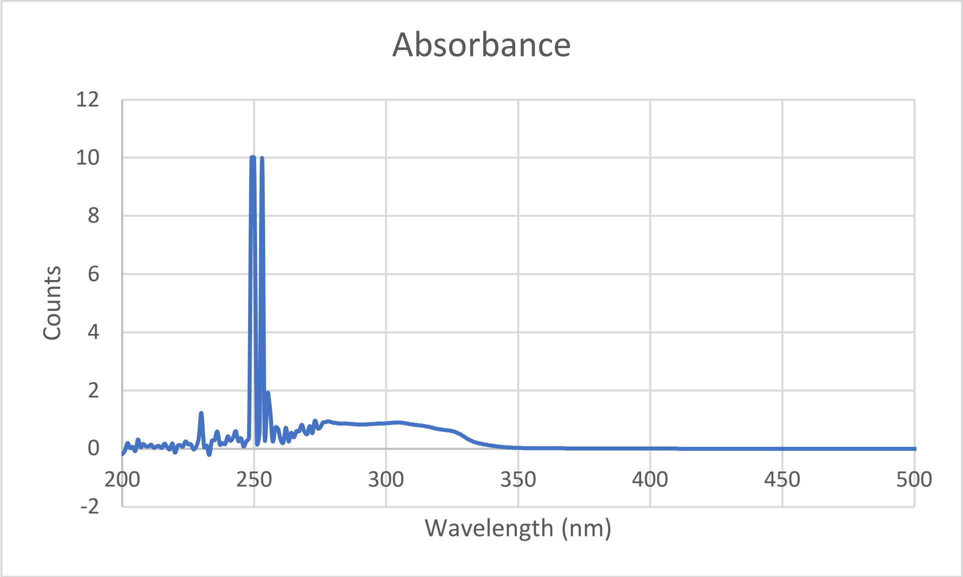
Absorbance
Counts
Wavelength (nm)

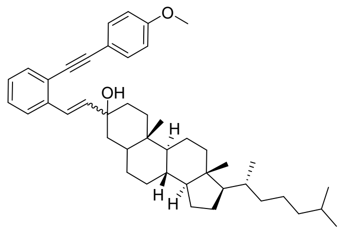

ATC-050522-472-S3steroid.1.fid

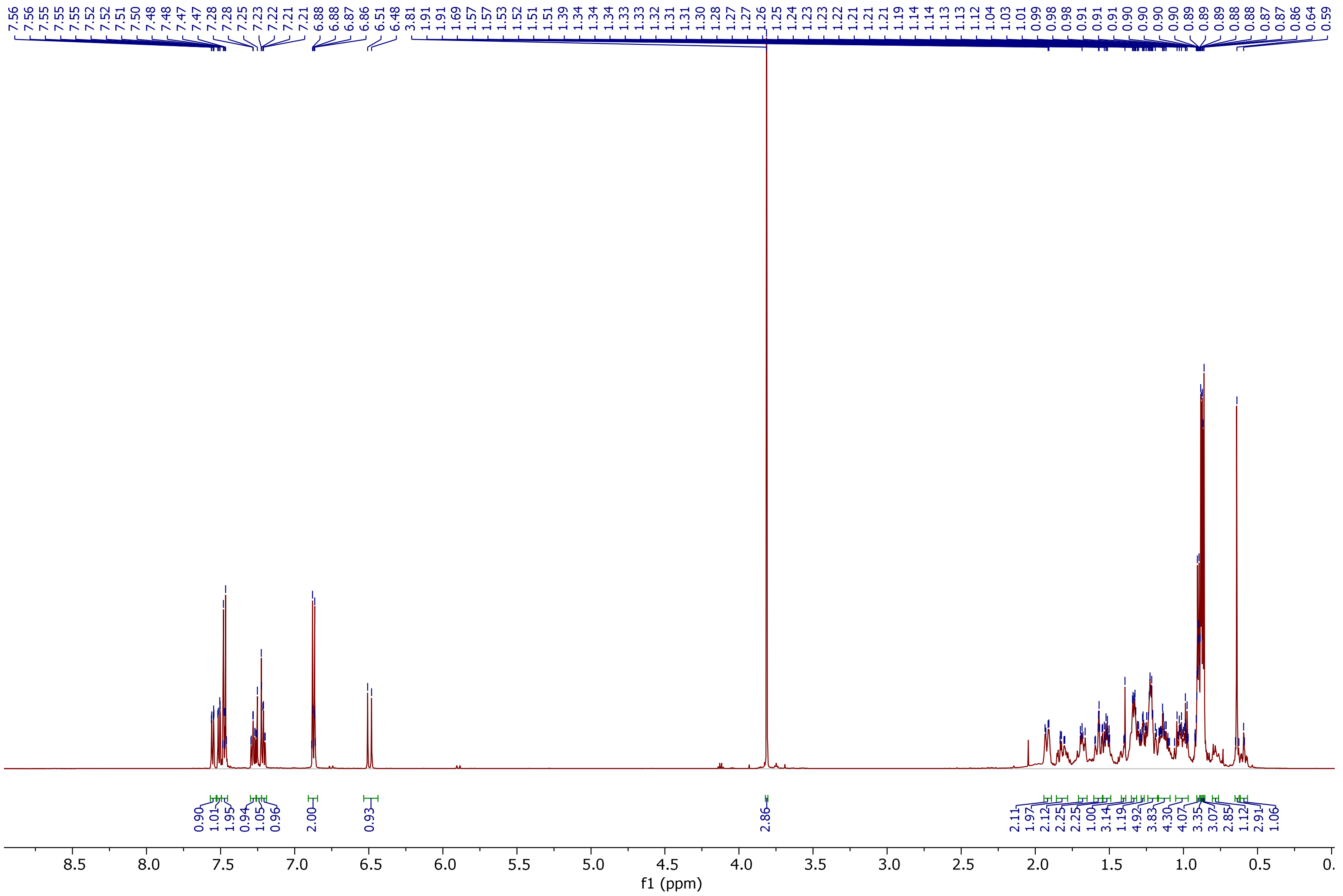

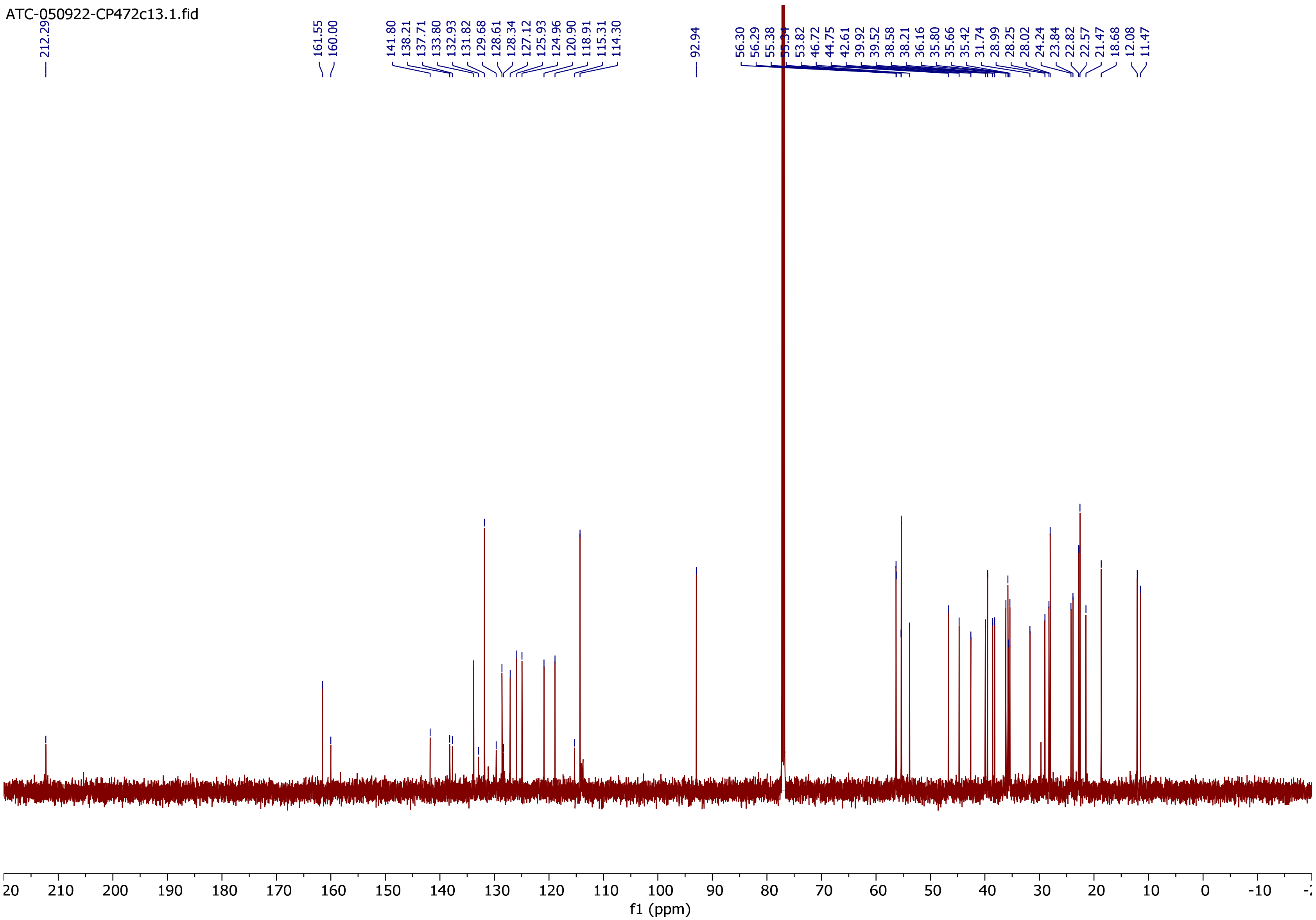
ATC-050922-CP472c13.1.fid
212.29
161.55
160.00
141.80
138.21
137.71
133.80
132.93
131.82
129.68
128.61
128.34
127.12
125.93
124.96
120.90
118.91
115.31
114.30
92.94
56.30
56.29
55.38
53.82
46.72
44.75
42.61
39.92
39.52
38.58
38.21
36.16
35.80
35.66
35.42
31.74
28.99
28.25
28.02
24.24
23.84
22.82
22.57
21.47
18.68
12.08
11.47
f1 (ppm)

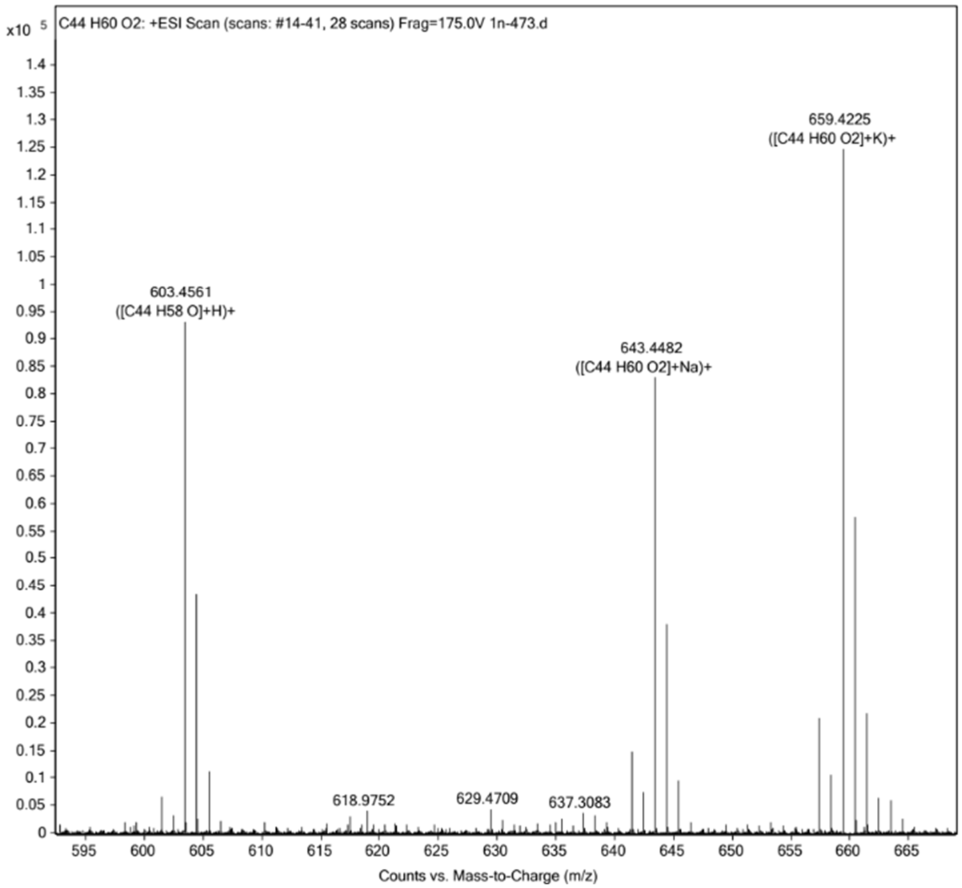

C44 H60 O2: +ESI Scan (scans: #14-41, 28 scans) Frag=175.0V 1n-473.d
x10 5
603.4561
([C44 H58 O]+H)+
643.4482
([C44 H60 O2]+Na)+
659.4225
([C44 H60 O2]+K)+
618.9752
629.4709
637.3083
Counts vs. Mass-to-Charge (m/z)

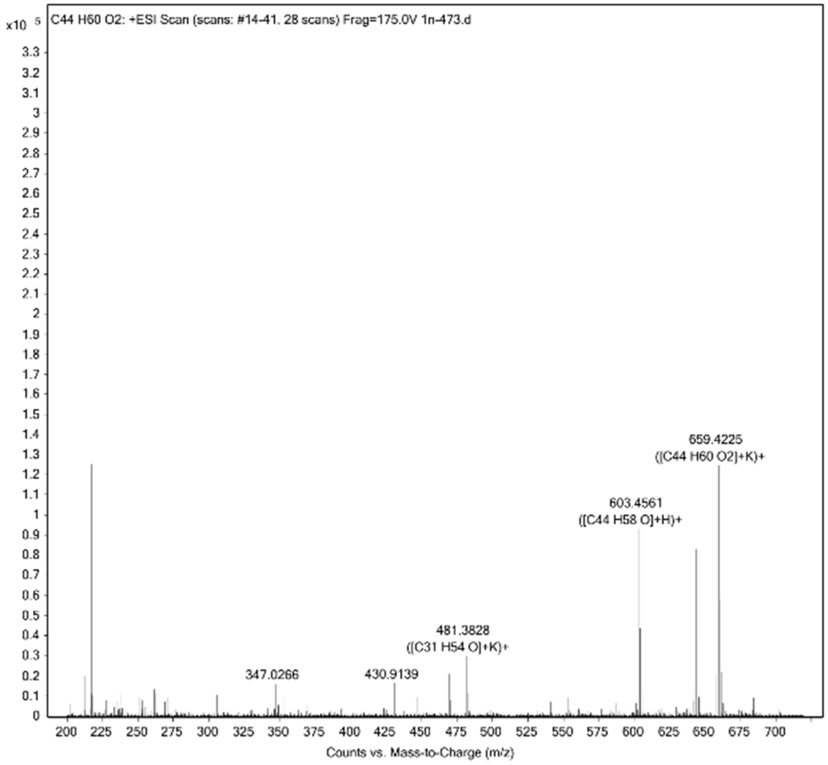

C44 H60 O2: +ESI Scan (scans: #14-41, 28 scans) Frag=175.0V 1n-473.d
x10 5
659.4225
([C44 H60 O2]+K)+
603.4561
([C44 H58 O]+H)+
481.3828
([C31 H54 O]+K)+
347.0266
430.9139
Counts vs. Mass-to-Charge (m/z)

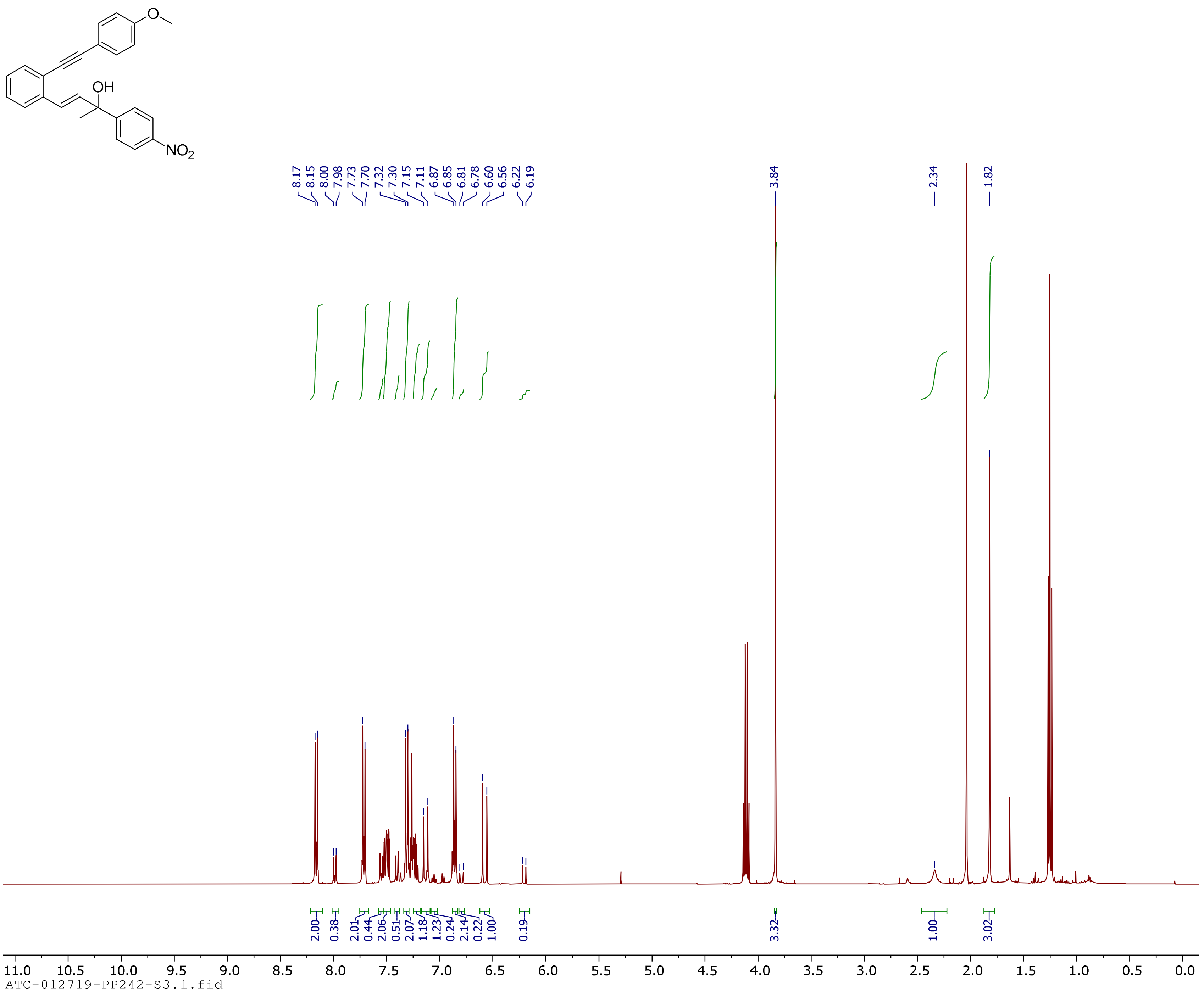

ATC-012719-PP242-S3.1.fid —

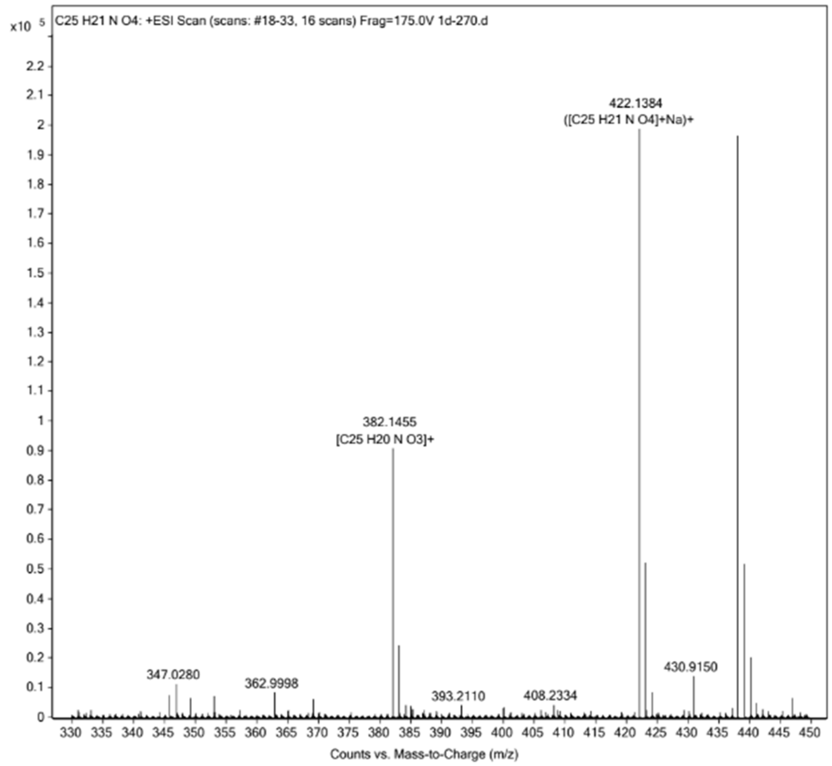
C25 H21 N O4: +ESI Scan (scans: #18-33, 16 scans) Frag=175.0V 1d-270.d
x10 5
422.1384
([C25 H21 N O4]+Na)+
382.1455
[C25 H20 N O3]+
347.0280
362.9998
393.2110
408.2334
430.9150
Counts vs. Mass-to-Charge (m/z)

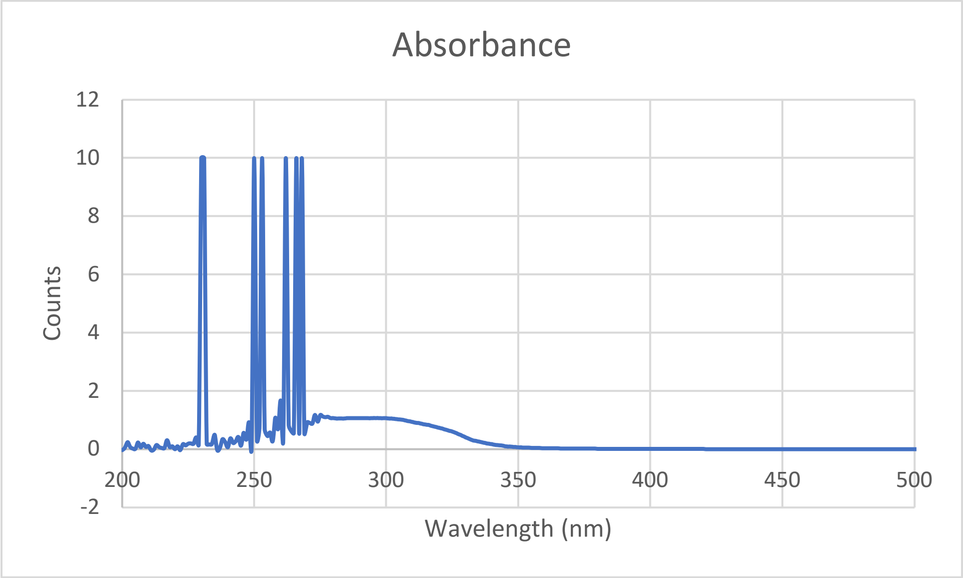
Absorbance
Counts
Wavelength (nm)

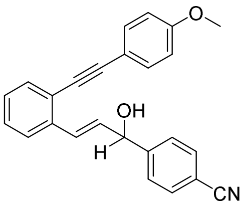
O
OH
H
CN

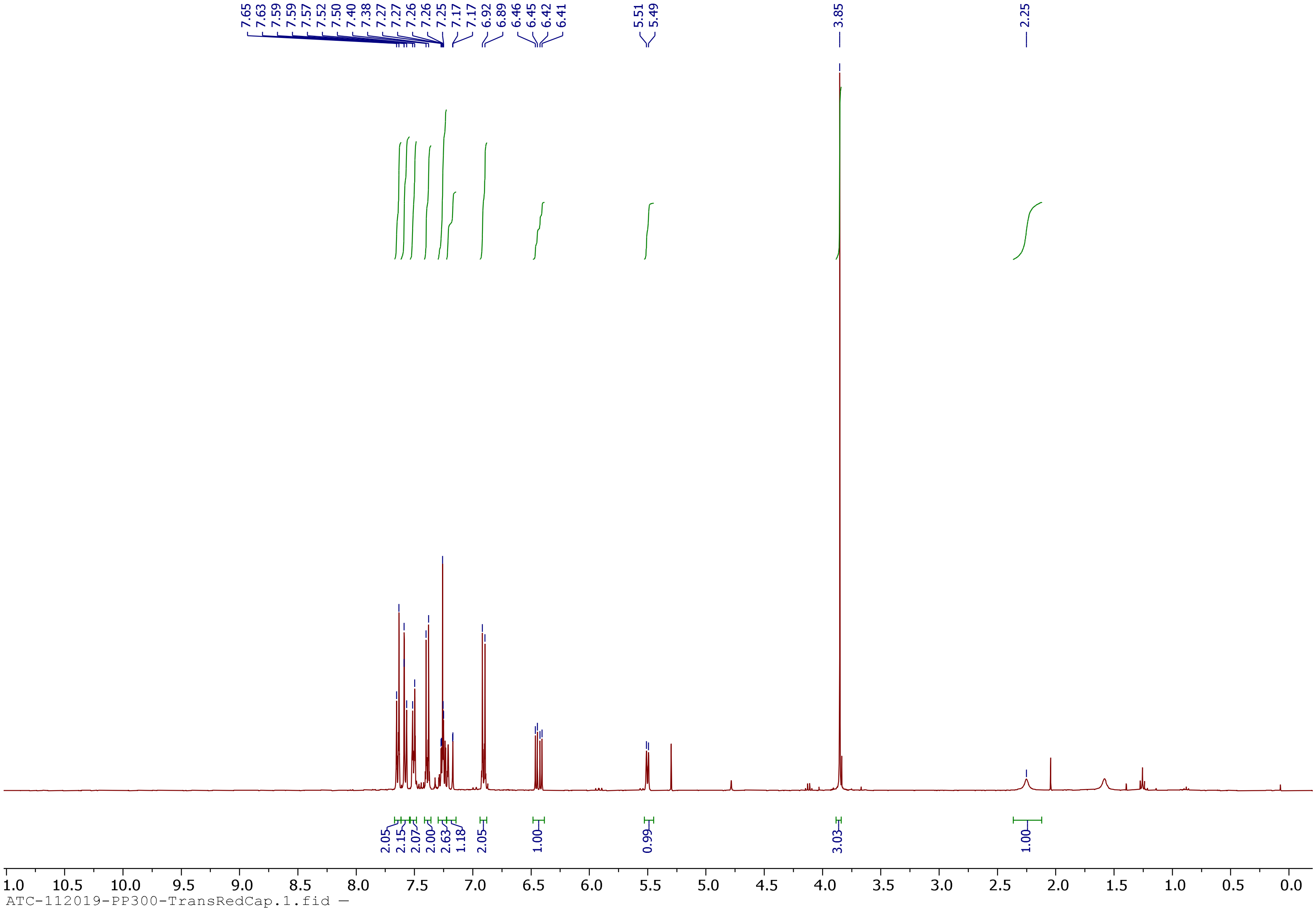
7.65
7.63
7.59
7.59
7.57
7.52
7.50
7.40
7.38
7.27
7.26
7.26
7.25
7.17
7.17
6.92
6.89
6.46
6.45
6.42
6.41
5.51
5.49
3.85
2.25
2.05
2.15
2.07
2.00
2.63
1.18
2.05
1.00
0.99
3.03
1.00
1.0 10.5 10.0 9.5 9.0 8.5 8.0 7.5 7.0 6.5 6.0 5.5 5.0 4.5 4.0 3.5 3.0 2.5 2.0 1.5 1.0 0.5 0.0
ATC-112019-PP300-TransRedCap.1.fid —

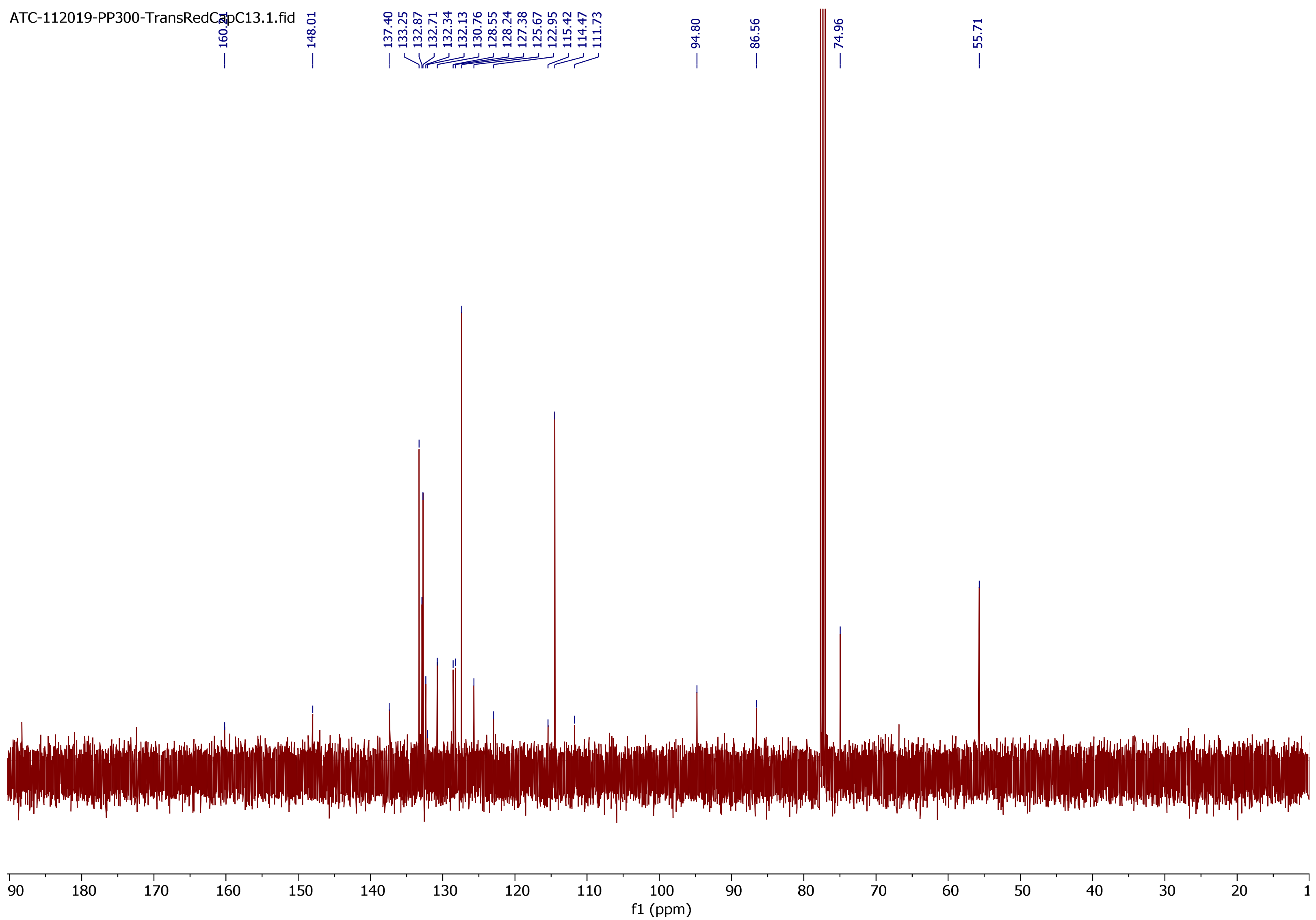
ATC-112019-PP300-TransRedCapC13.1.fid
160.
148.01
137.40
133.25
132.87
132.71
132.34
132.13
130.76
128.55
128.24
127.38
125.67
122.95
115.42
114.47
111.73
94.80
86.56
74.96
55.71
f1 (ppm)

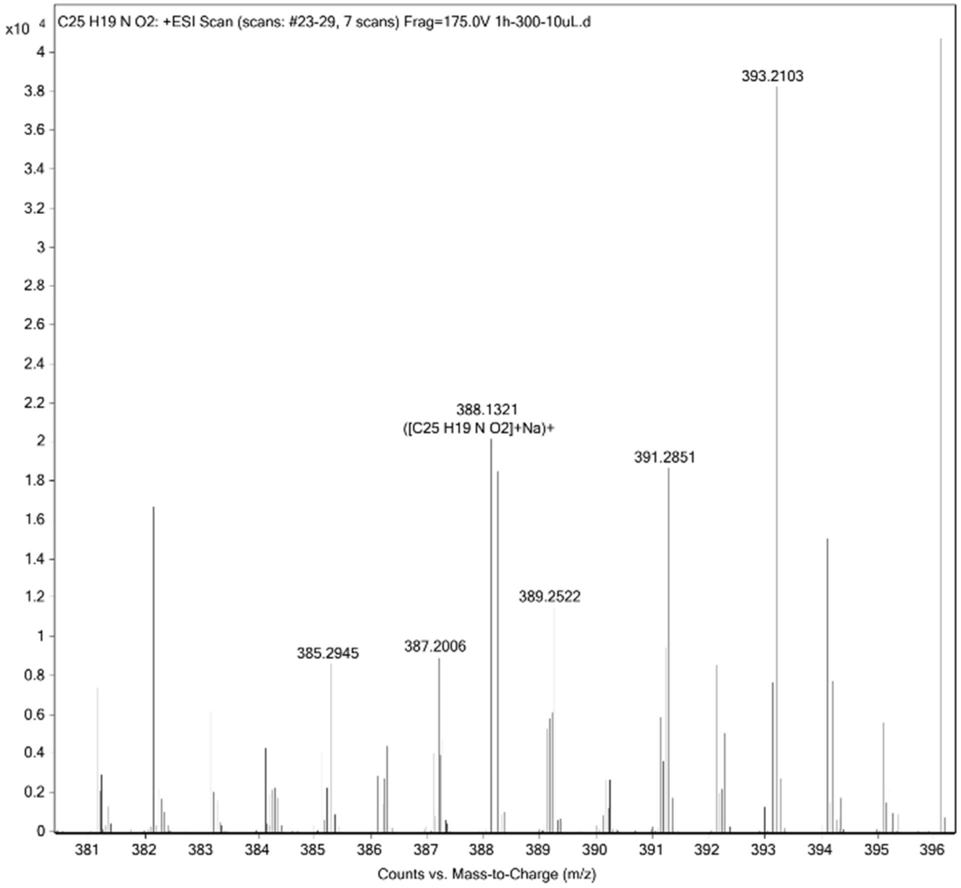

C25 H19 N O2: +ESI Scan (scans: #23-29, 7 scans) Frag=175.0V 1h-300-10uL.d
x10 4
393.2103
388.1321
([C25 H19 N O2]+Na)+
391.2851
389.2522
385.2945
387.2006
Counts vs. Mass-to-Charge (m/z)

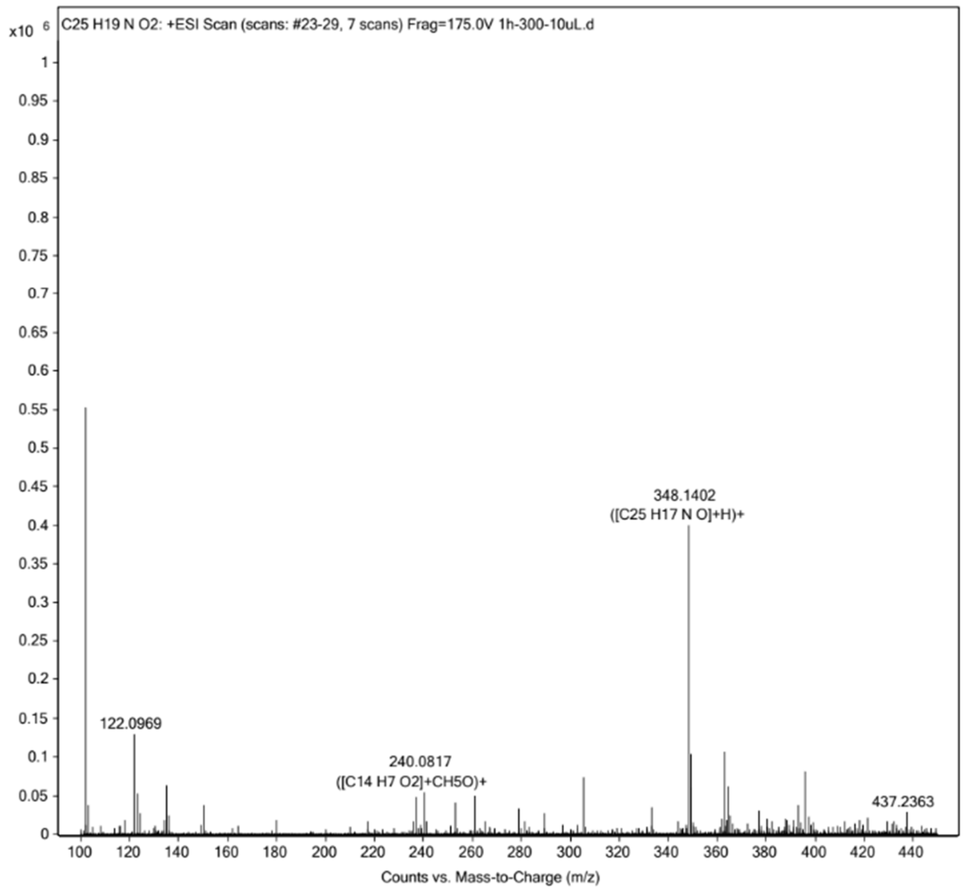

x10 6
C25 H19 N O2: +ESI Scan (scans: #23-29, 7 scans) Frag=175.0V 1h-300-10uL.d
122.0969
240.0817
([C14 H7 O2]+CH5O)+
348.1402
([C25 H17 N O]+H)+
437.2363
Counts vs. Mass-to-Charge (m/z)

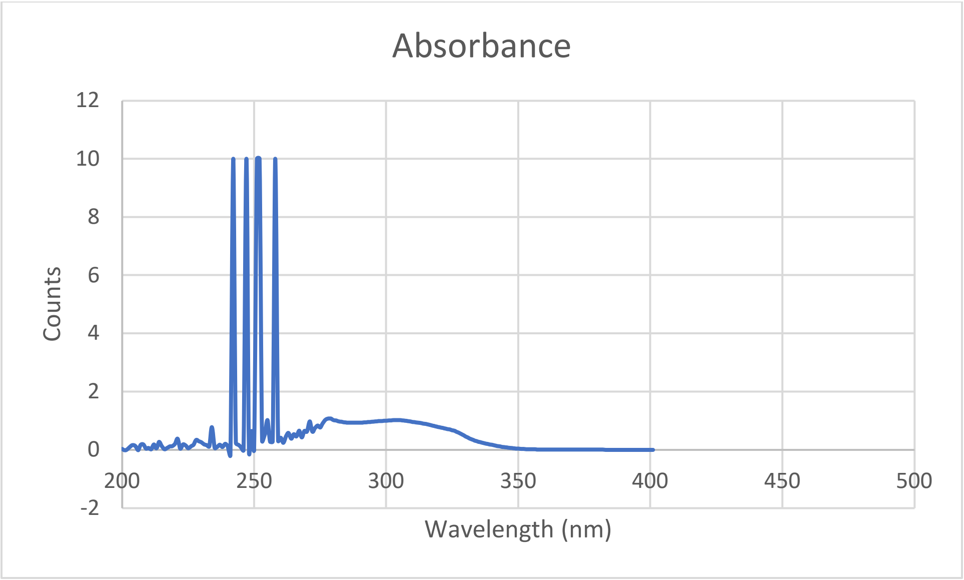
Absorbance
Counts
12
10
8
6
4
2
0
-2
200
250
300
350
400
450
500
Wavelength (nm)

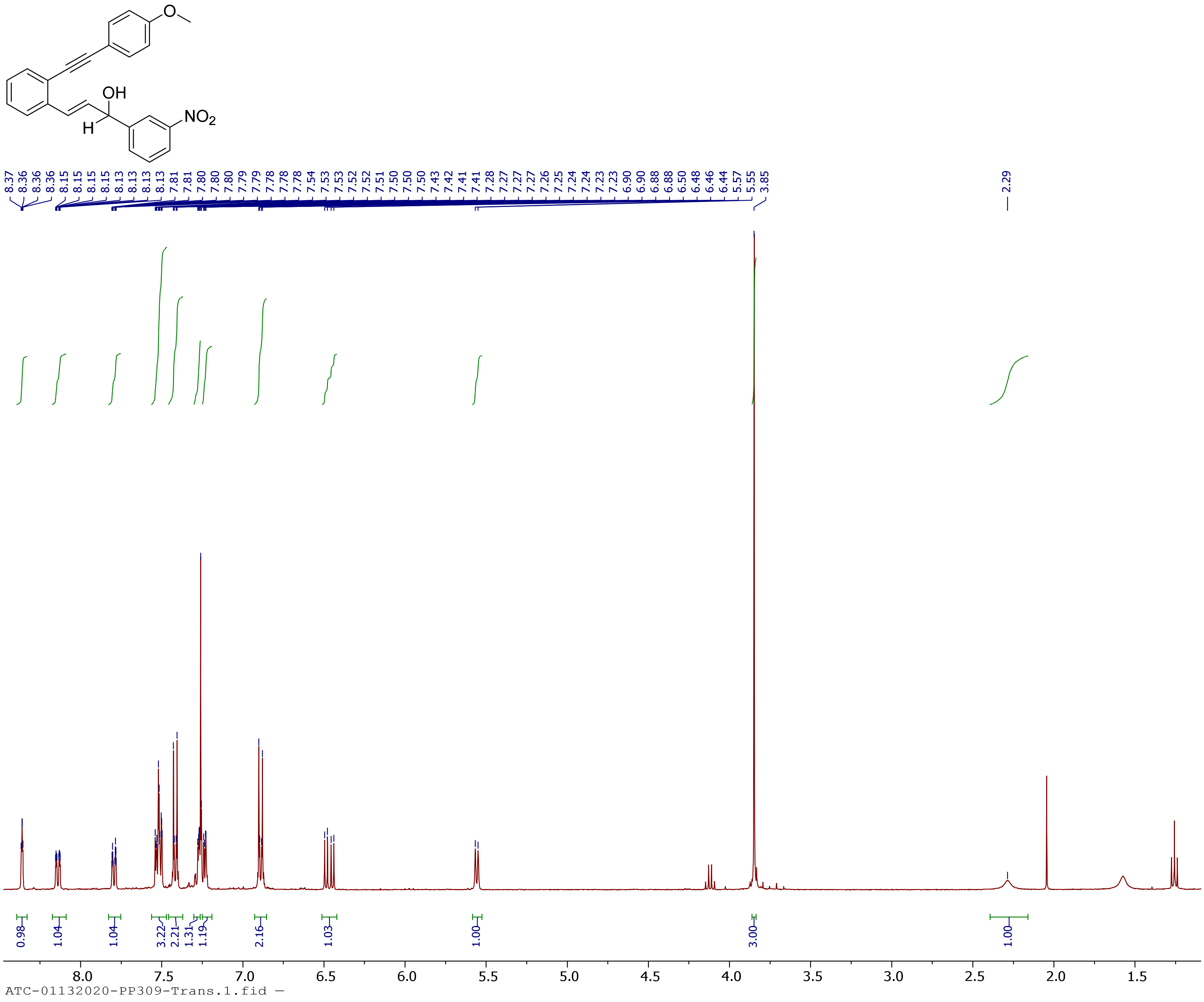
OH
H
NO2
O
8.37 8.36 8.36 8.36 8.15 8.15 8.15 8.15 8.13 8.13 8.13 8.13 7.81 7.81 7.80 7.80 7.80 7.79 7.78 7.78 7.78 7.54 7.53 7.53 7.52 7.52 7.51 7.50 7.50 7.50 7.43 7.42 7.41 7.41 7.28 7.27 7.27 7.27 7.26 7.25 7.24 7.24 7.23 7.23 6.90 6.90 6.88 6.88 6.50 6.48 6.46 6.44 5.57 5.55 3.85
2.29
0.98 1.04 1.04 3.22 2.21 1.31 1.19 2.16 1.03 1.00 3.00 1.00
8.0 7.5 7.0 6.5 6.0 5.5 5.0 4.5 4.0 3.5 3.0 2.5 2.0 1.5
ATC-01132020-PP309-Trans.1.fid —

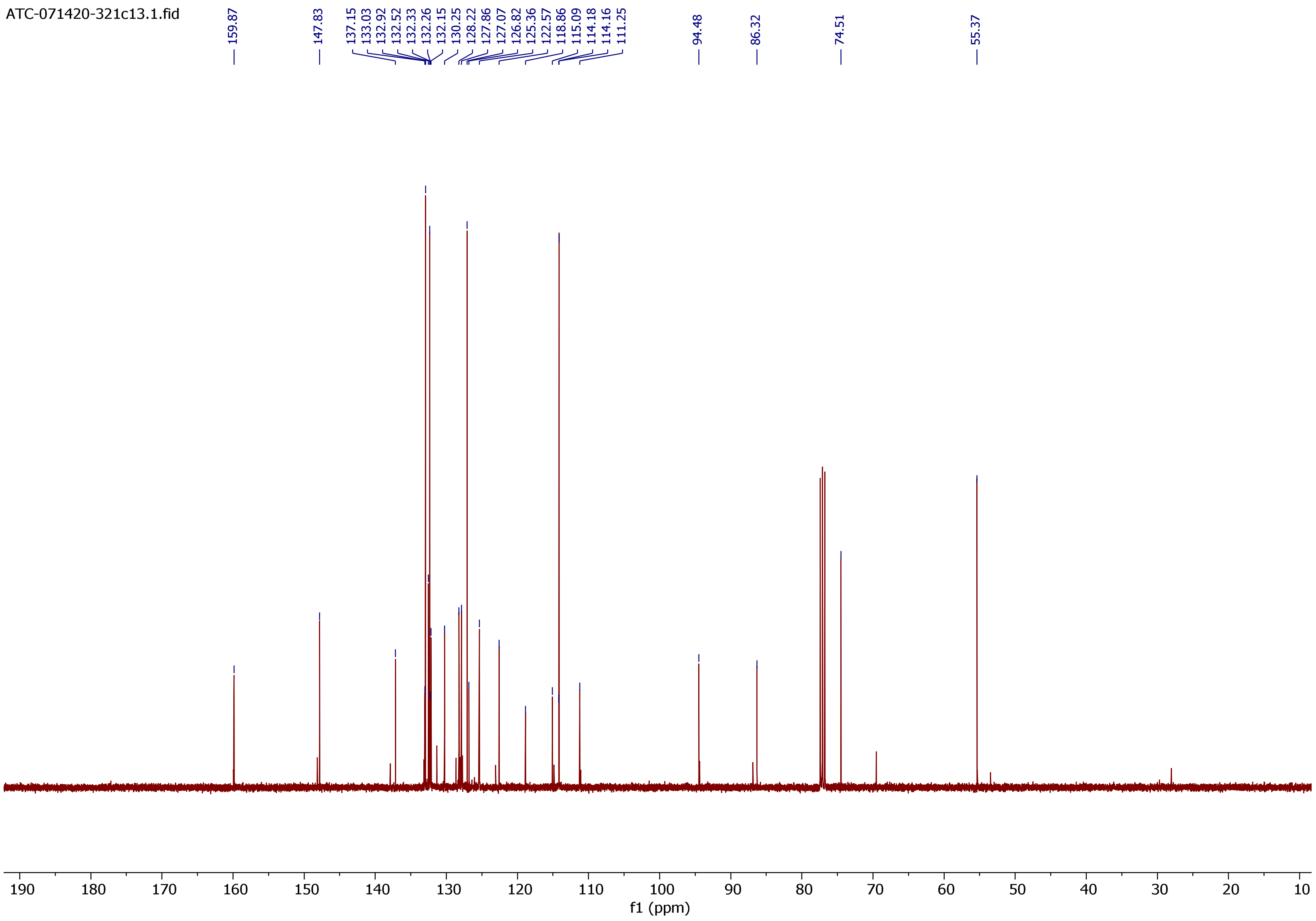
ATC-071420-321c13.1.fid
159.87
147.83
137.15
133.03
132.92
132.52
132.33
132.26
132.15
130.25
128.22
127.86
127.07
126.82
125.36
122.57
118.86
115.09
114.18
114.16
111.25
94.48
86.32
74.51
55.37
190
180
170
160
150
140
130
120
110
100
90
80
70
60
50
40
30
20
10
f1 (ppm)

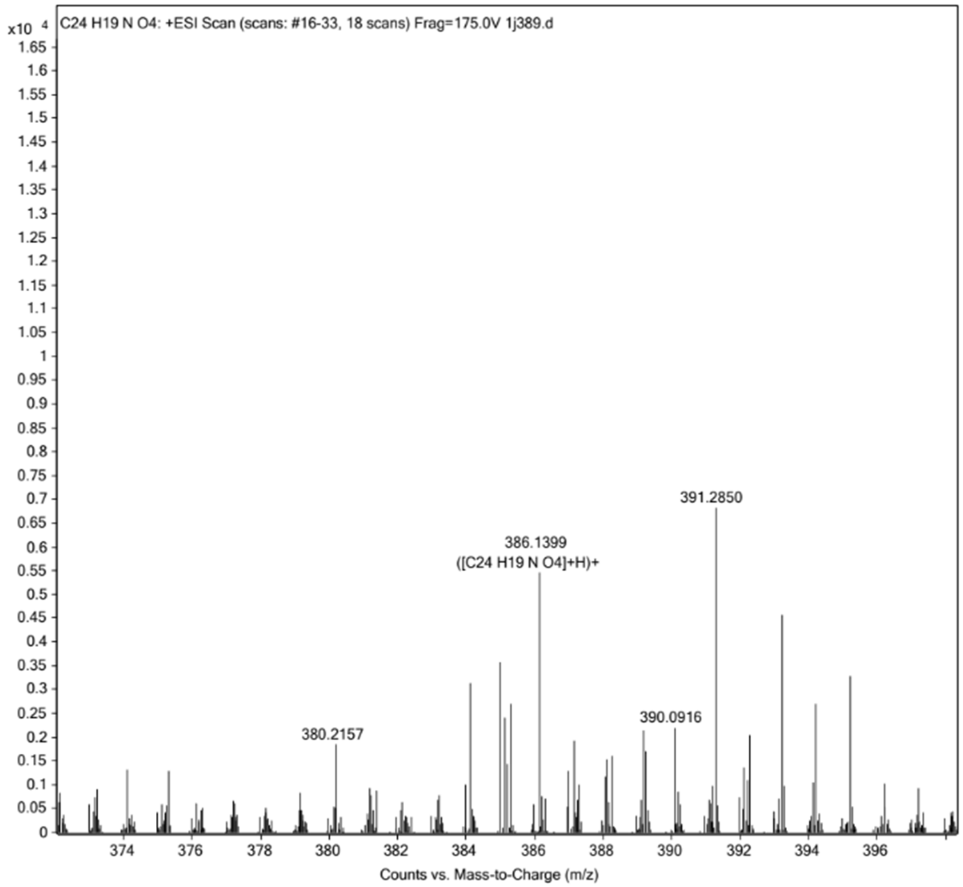
C24 H19 N O4: +ESI Scan (scans: #16-33, 18 scans) Frag=175.0V 1j389.d
x10 4
391.2850
386.1399
([C24 H19 N O4]+H)+
390.0916
380.2157
Counts vs. Mass-to-Charge (m/z)

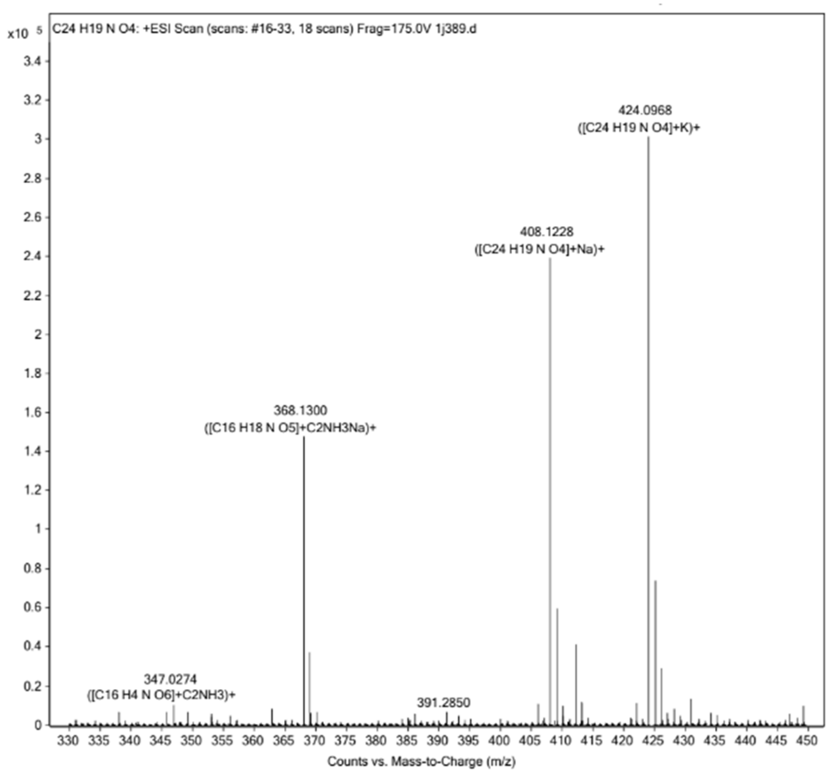
x10 5
C24 H19 N O4: +ESI Scan (scans: #16-33, 18 scans) Frag=175.0V 1j389.d
424.0968
([C24 H19 N O4]+K)+
408.1228
([C24 H19 N O4]+Na)+
368.1300
([C16 H18 N O5]+C2NH3Na)+
347.0274
([C16 H4 N O6]+C2NH3)+
391.2850
Counts vs. Mass-to-Charge (m/z)

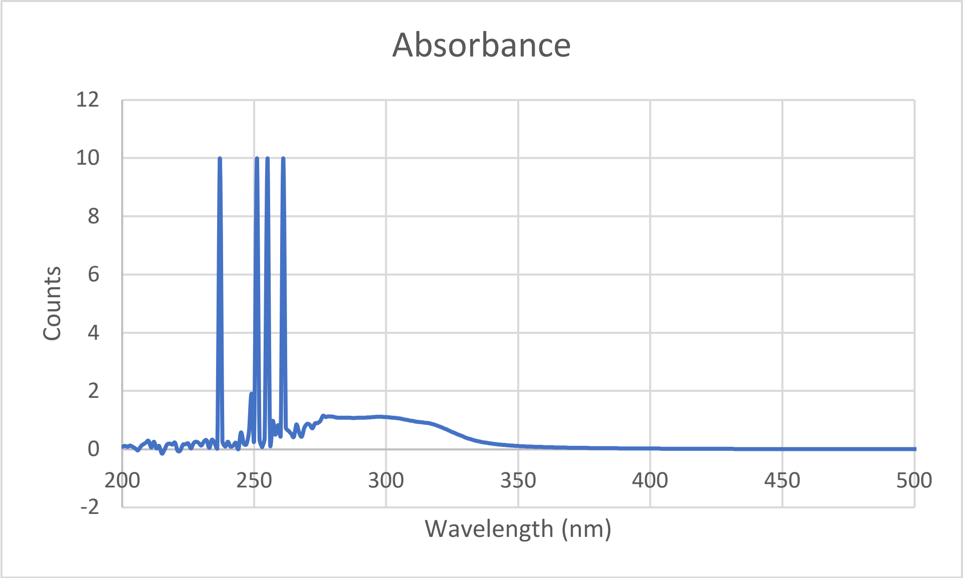
Absorbance
Counts
Wavelength (nm)

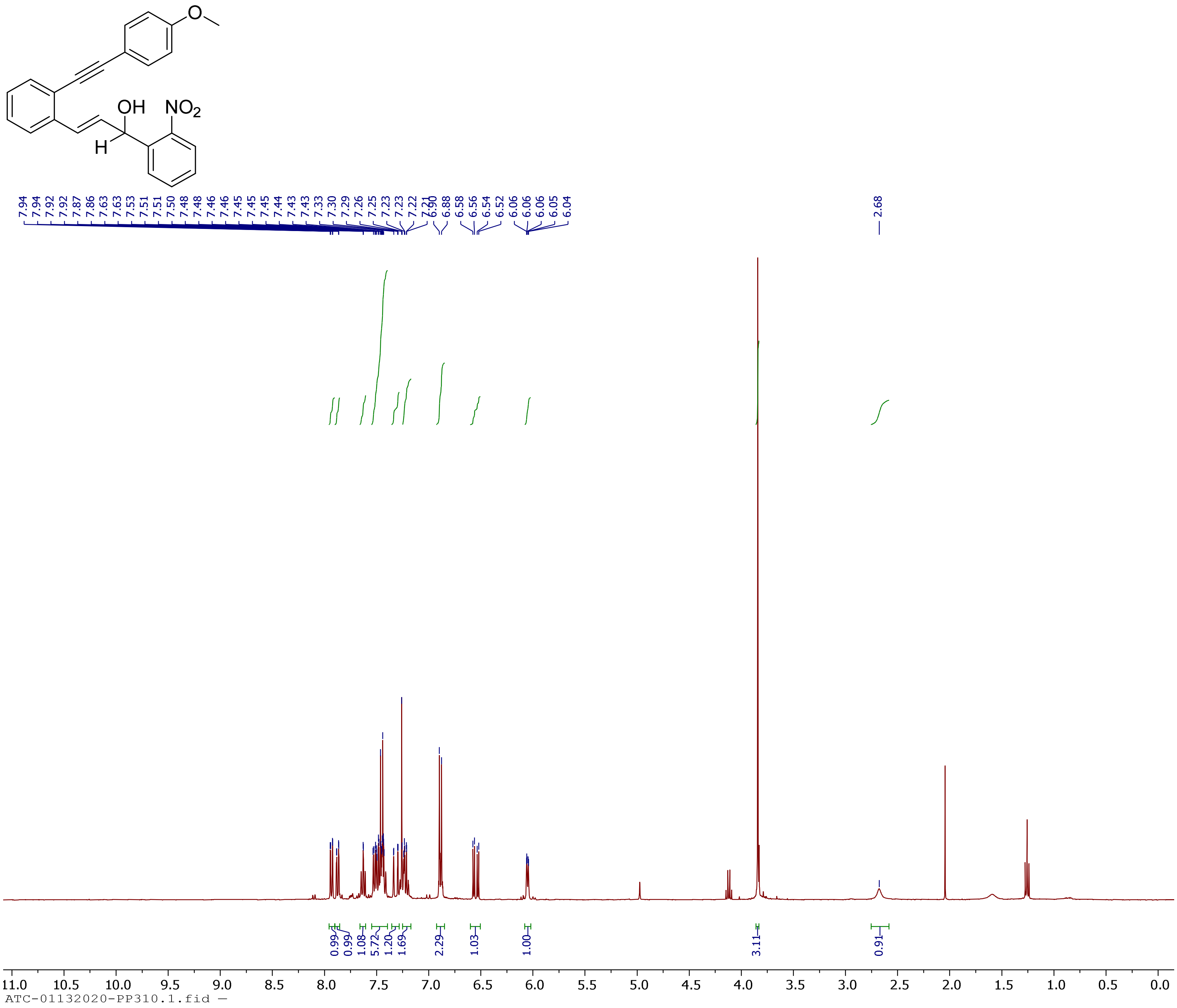

OH
NO2
H
O
7.94
7.94
7.92
7.92
7.87
7.86
7.63
7.63
7.53
7.51
7.51
7.50
7.48
7.48
7.46
7.46
7.45
7.45
7.45
7.44
7.43
7.43
7.33
7.30
7.29
7.26
7.25
7.23
7.23
7.22
7.21
6.90
6.88
6.58
6.56
6.54
6.52
6.06
6.06
6.06
6.05
6.04
2.68
0.99
0.99
1.08
5.72
1.20
1.69
2.29
1.03
1.00
3.11
0.91
11.0
10.5
10.0
9.5
9.0
8.5
8.0
7.5
7.0
6.5
6.0
5.5
5.0
4.5
4.0
3.5
3.0
2.5
2.0
1.5
1.0
0.5
0.0
ATC-01132020-PP310.1.fid —

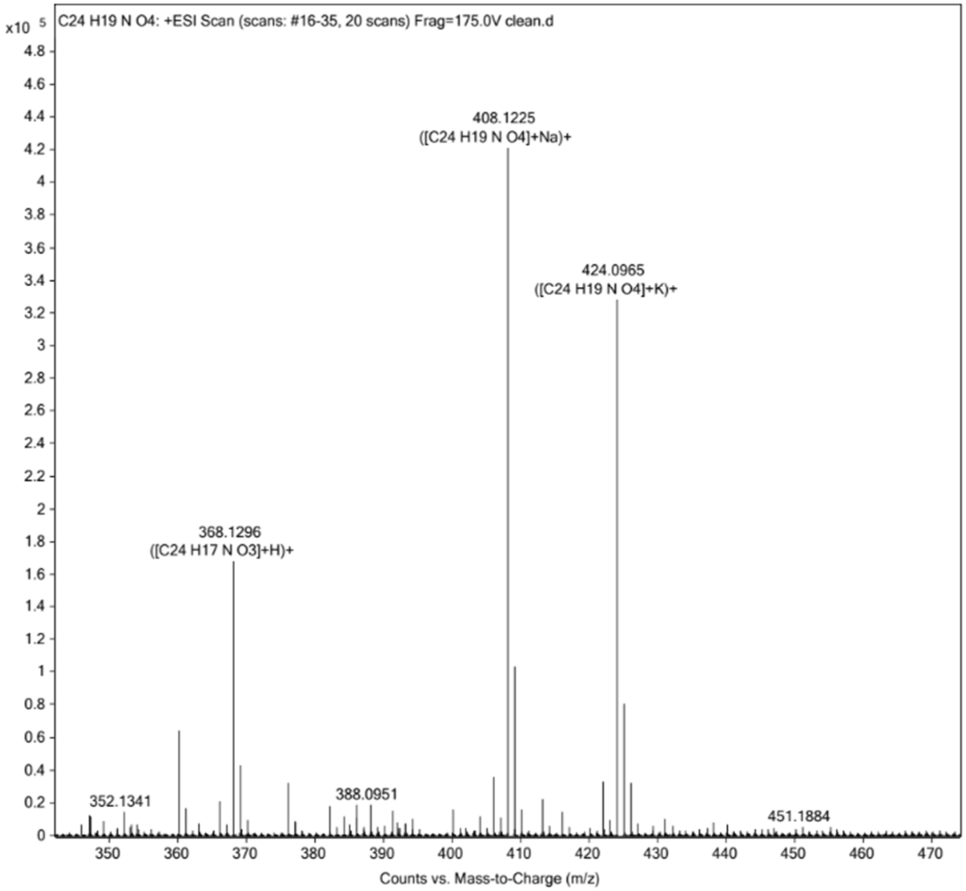

C24 H19 N O4: +ESI Scan (scans: #16-35, 20 scans) Frag=175.0V clean.d
x10 5
408.1225
([C24 H19 N O4]+Na)+
424.0965
([C24 H19 N O4]+K)+
368.1296
([C24 H17 N O3]+H)+
352.1341
388.0951
451.1884
Counts vs. Mass-to-Charge (m/z)

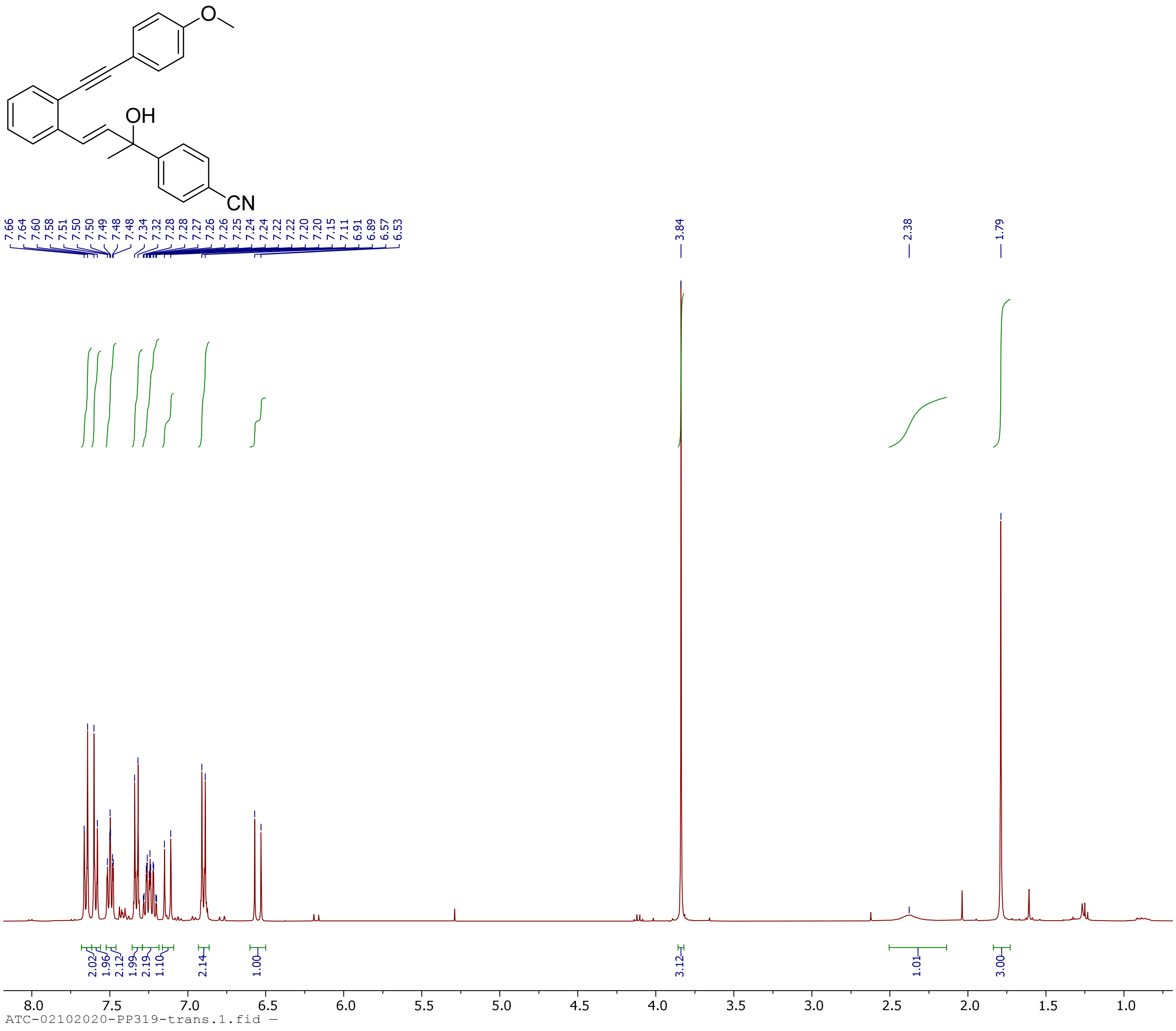
O
OH
CN
7.66
7.64
7.60
7.58
7.51
7.50
7.50
7.49
7.48
7.48
7.34
7.32
7.28
7.28
7.27
7.26
7.26
7.25
7.24
7.24
7.22
7.22
7.20
7.20
7.15
7.11
6.91
6.89
6.57
6.53
3.84
2.38
1.79
2.02
1.96
2.12
1.99
2.19
1.10
2.14
1.00
3.12
1.01
3.00
8.0
7.5
7.0
6.5
6.0
5.5
5.0
4.5
4.0
3.5
3.0
2.5
2.0
1.5
1.0
ATC-02102020-PP319-trans.1.fid

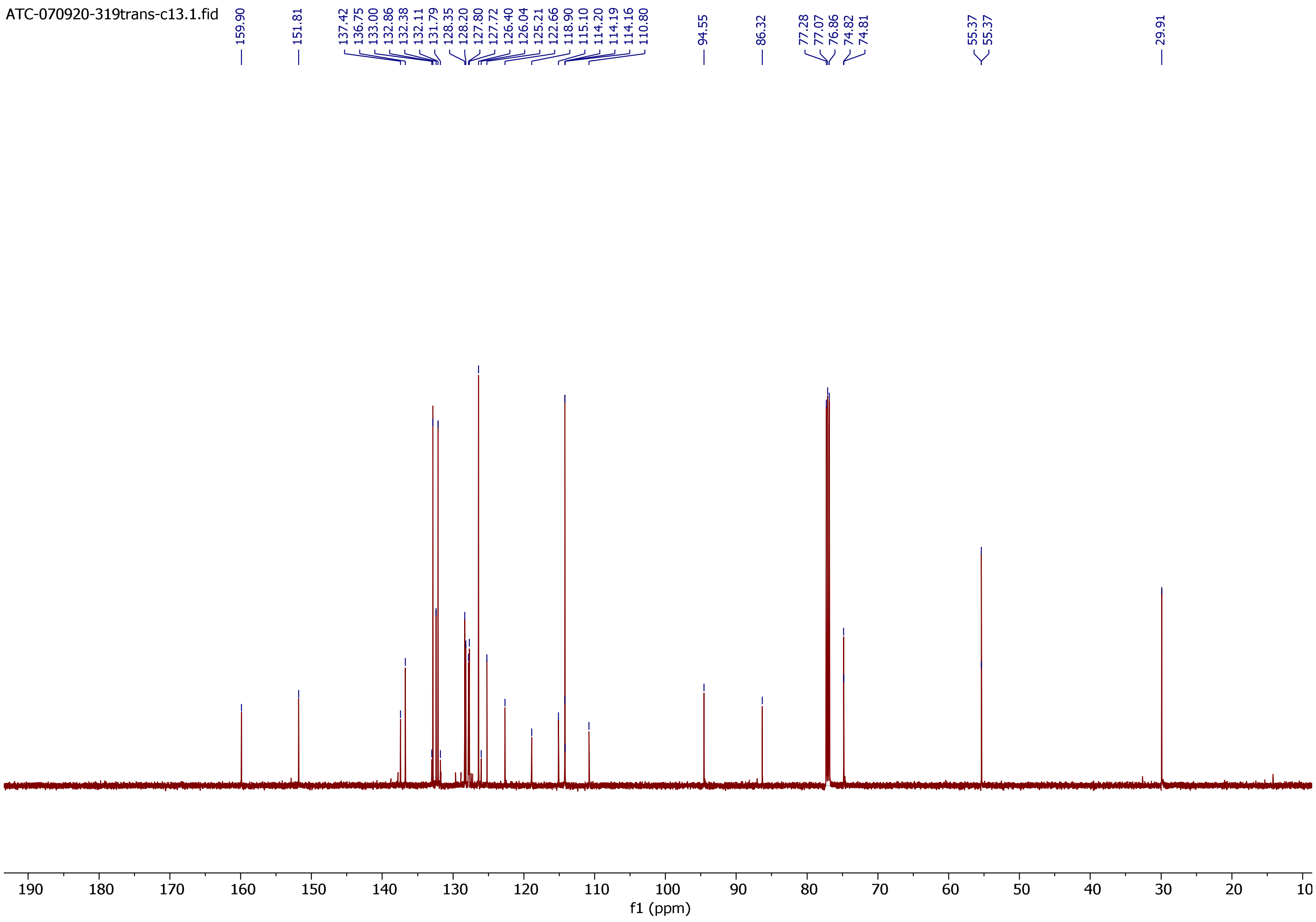
ATC-070920-319trans-c13.1.fid
159.90
151.81
137.42
136.75
133.00
132.86
132.38
132.11
131.79
128.35
128.20
127.80
127.72
126.40
126.04
125.21
122.66
118.90
115.10
114.20
114.19
114.16
110.80
94.55
86.32
77.28
77.07
76.86
74.82
74.81
55.37
55.37
29.91
190
180
170
160
150
140
130
120
110
100
90
80
70
60
50
40
30
20
10
f1 (ppm)

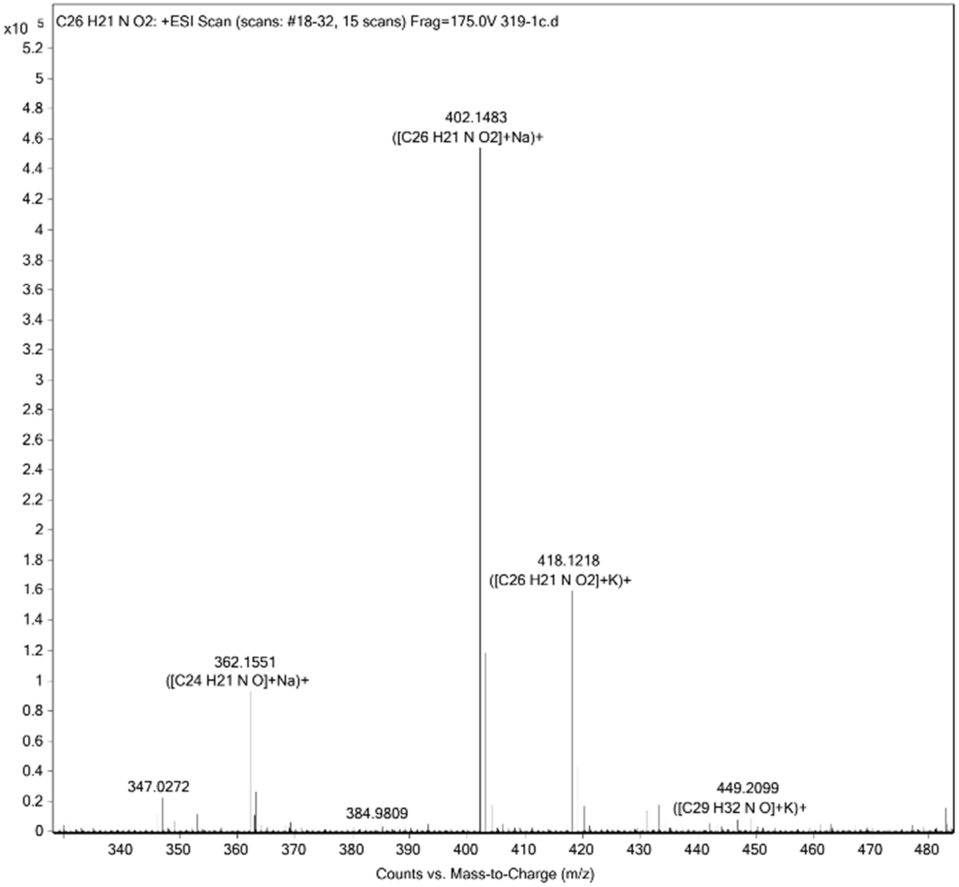

C26 H21 N O2: +ESI Scan (scans: #18-32, 15 scans) Frag=175.0V 319-1c.d
x10 5
402.1483
([C26 H21 N O2]+Na)+
418.1218
([C26 H21 N O2]+K)+
362.1551
([C24 H21 N O]+Na)+
347.0272
384.9809
449.2099
([C29 H32 N O]+K)+
Counts vs. Mass-to-Charge (m/z)

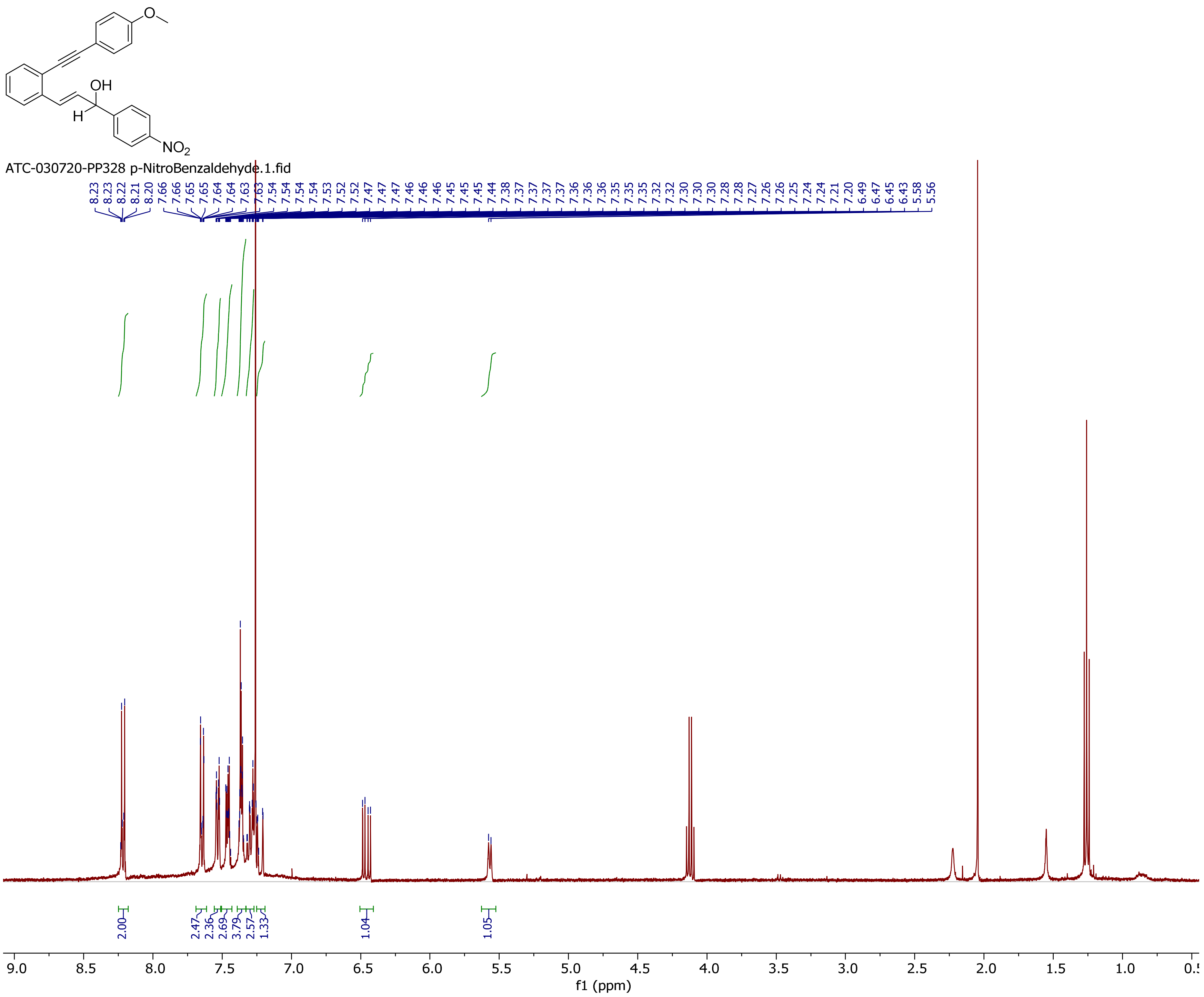

ATC-030720-PP328 p-NitroBenzaldehyde.1.fid
8.23 8.23 8.22 8.21 8.20 7.66 7.65 7.65 7.64 7.64 7.63 7.63 7.54 7.54 7.54 7.54 7.53 7.52 7.52 7.47 7.47 7.46 7.46 7.45 7.45 7.45 7.44 7.38 7.37 7.37 7.37 7.36 7.36 7.35 7.35 7.32 7.32 7.30 7.30 7.28 7.28 7.27 7.26 7.26 7.25 7.24 7.24 7.21 7.20 6.49 6.47 6.45 6.43 5.58 5.56
2.00 2.47 2.36 2.69 3.79 2.57 1.33 1.04 1.05
9.0 8.5 8.0 7.5 7.0 6.5 6.0 5.5 5.0 4.5 4.0 3.5 3.0 2.5 2.0 1.5 1.0 0.5
f1 (ppm)

[1]H Peak at 2.25 = 1H from OH. 1.56= Water

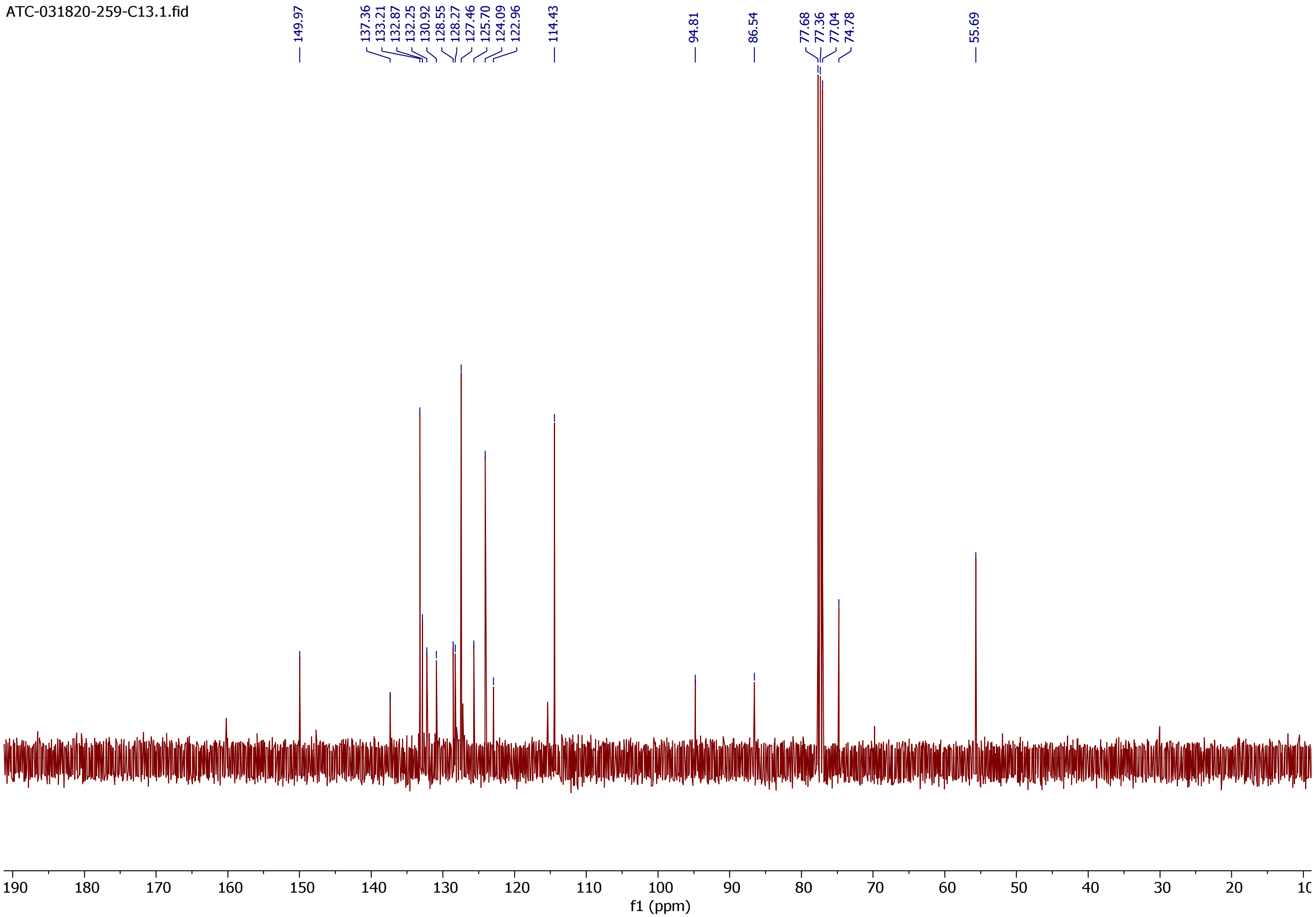

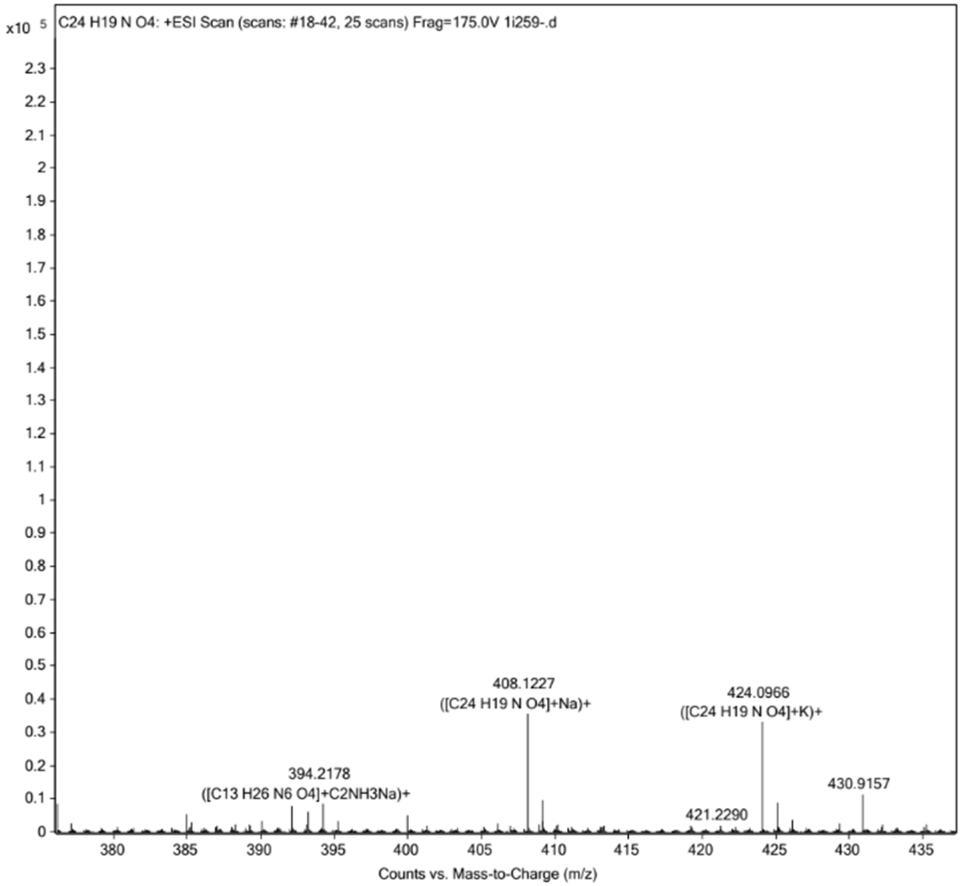

C24 H19 N O4: +ESI Scan (scans: #18-42, 25 scans) Frag=175.0V 1i259-.d
x10 5
408.1227
([C24 H19 N O4]+Na)+
424.0966
([C24 H19 N O4]+K)+
394.2178
([C13 H26 N6 O4]+C2NH3Na)+
430.9157
421.2290
Counts vs. Mass-to-Charge (m/z)

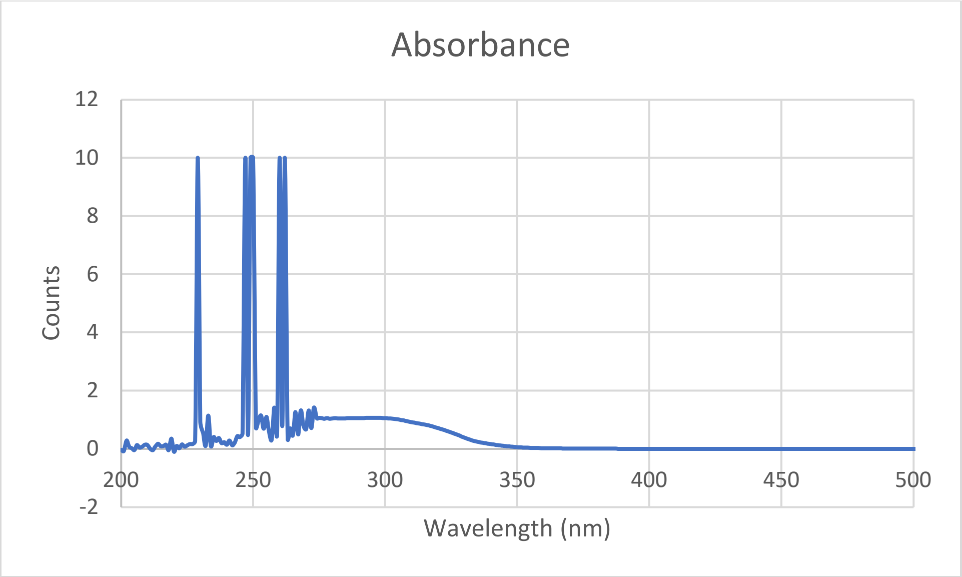
Absorbance
Counts
12
10
8
6
4
2
0
-2
200
250
300
350
400
450
500
Wavelength (nm)

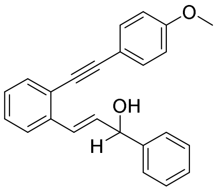

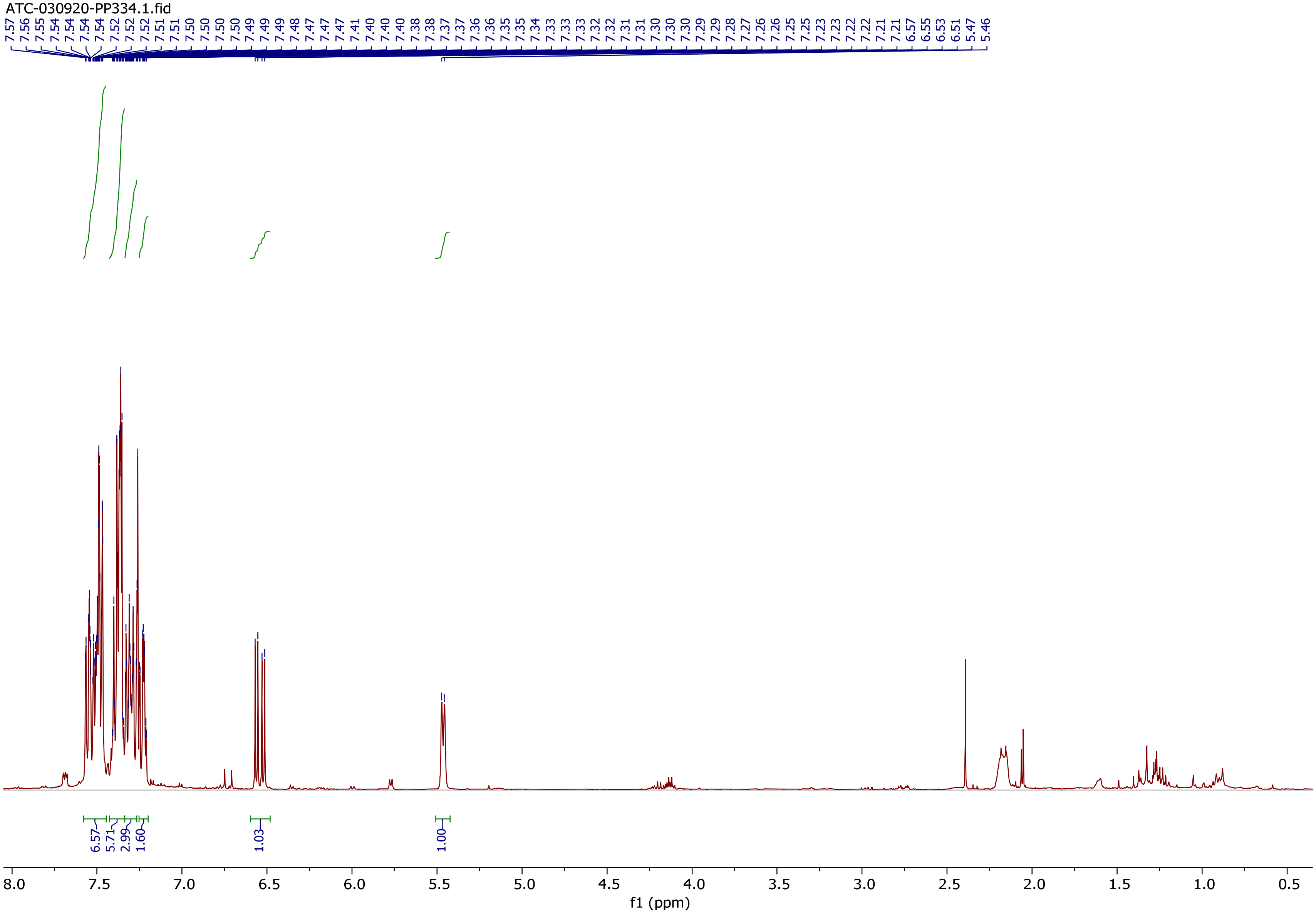

^{1}H broad Peak at 2.2 = 1H from OH. Singlet at 2.36 from Toluene. 1.56= Water.

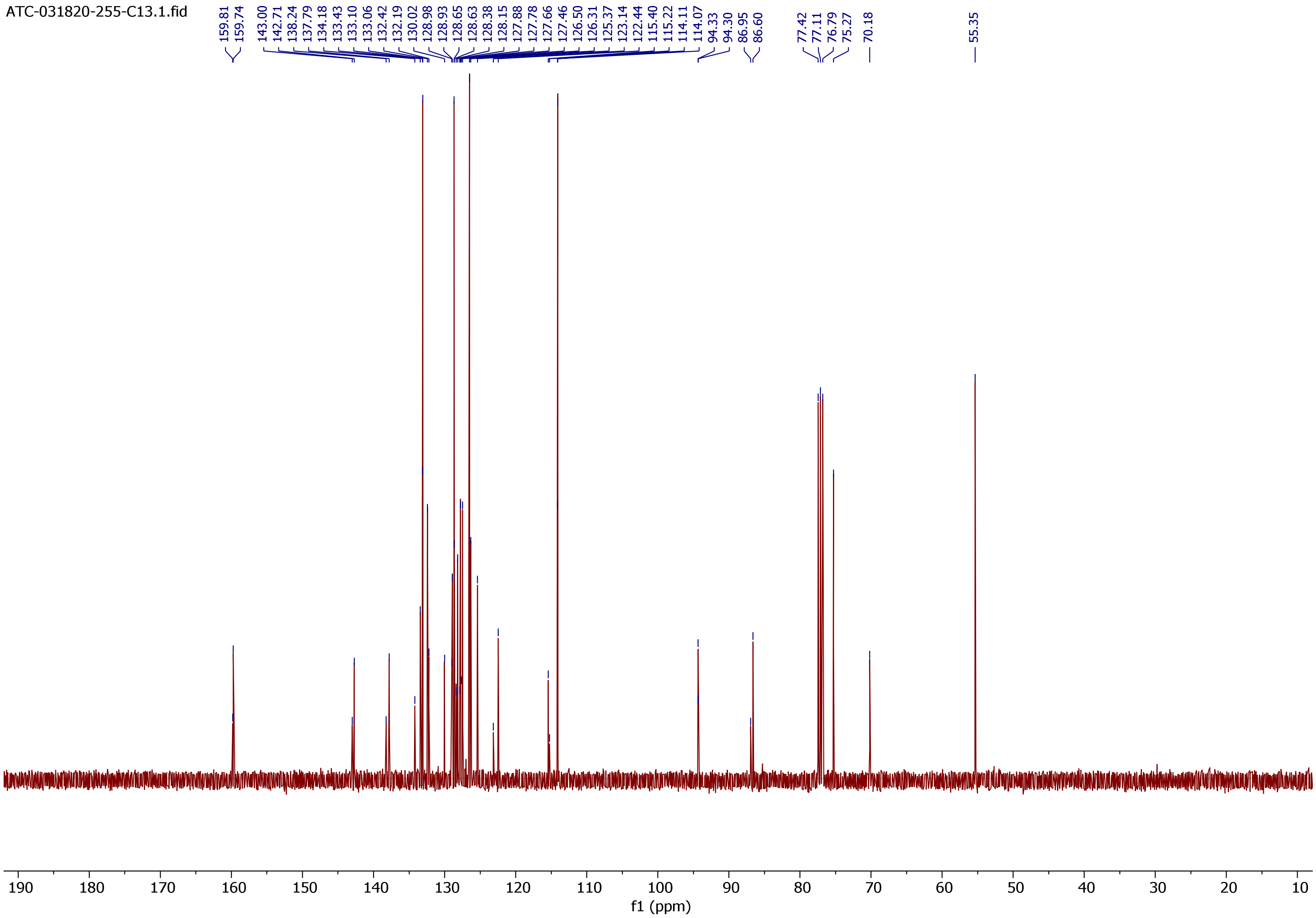
ATC-031820-255-C13.1.fid
159.81
159.74
143.00
142.71
138.24
137.79
134.18
133.43
133.10
133.06
132.42
132.19
130.02
128.98
128.93
128.65
128.63
128.38
128.15
127.88
127.78
127.66
127.46
126.50
126.31
125.37
123.14
122.44
115.40
115.22
114.11
114.07
94.33
94.30
86.95
86.60
77.42
77.11
76.79
75.27
70.18
55.35
190
180
170
160
150
140
130
120
110
100
90
80
70
60
50
40
30
20
10
f1 (ppm)

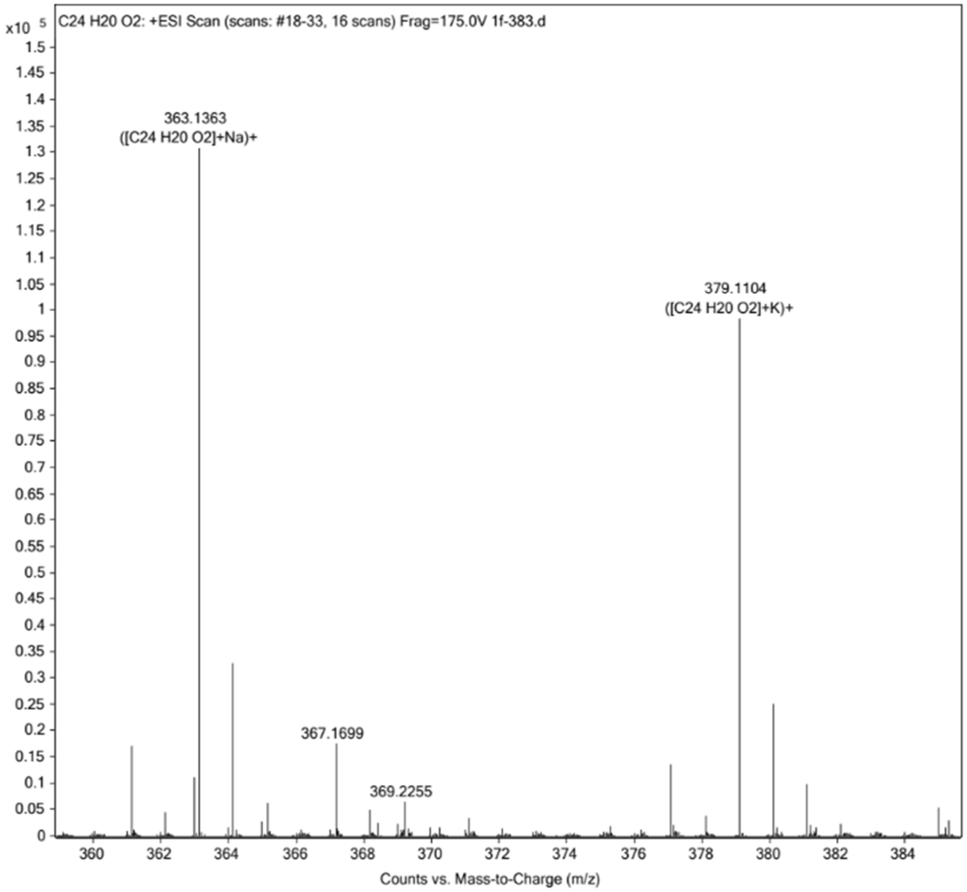

x10 5
C24 H20 O2: +ESI Scan (scans: #18-33, 16 scans) Frag=175.0V 1f-383.d
363.1363
([C24 H20 O2]+Na)+
379.1104
([C24 H20 O2]+K)+
367.1699
369.2255
1.5
1.45
1.4
1.35
1.3
1.25
1.2
1.15
1.1
1.05
1
0.95
0.9
0.85
0.8
0.75
0.7
0.65
0.6
0.55
0.5
0.45
0.4
0.35
0.3
0.25
0.2
0.15
0.1
0.05
0
360
362
364
366
368
370
372
374
376
378
380
382
384
Counts vs. Mass-to-Charge (m/z)

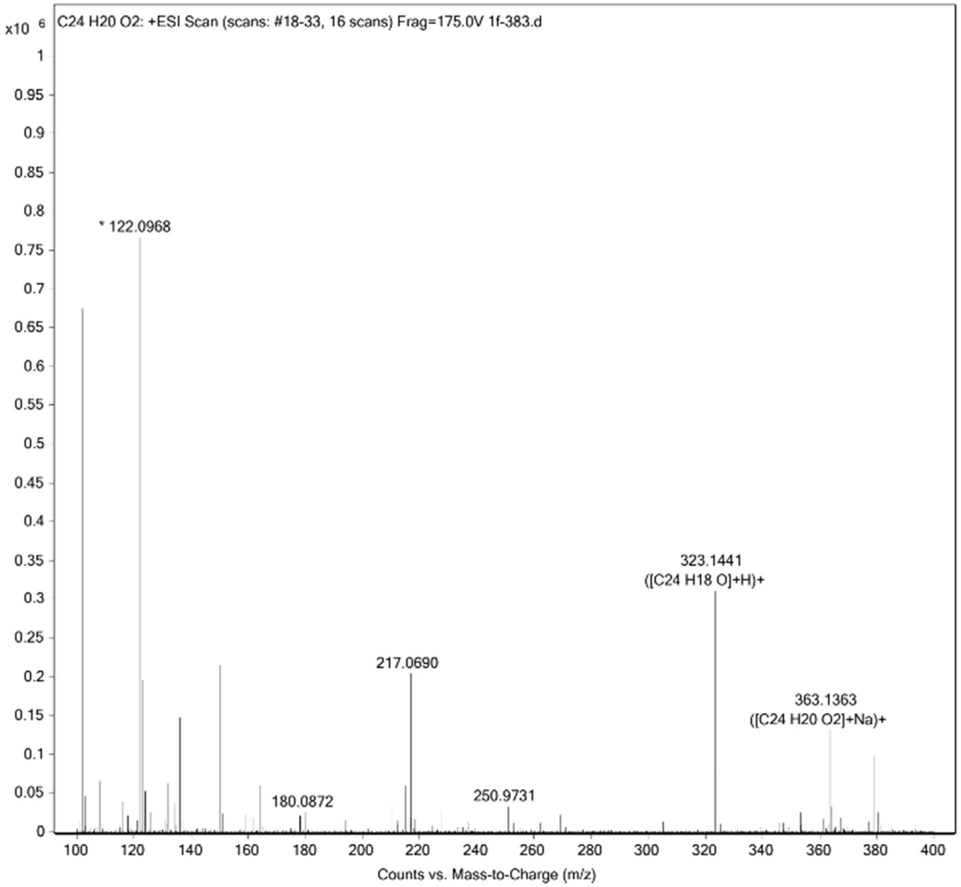

C24 H20 O2: +ESI Scan (scans: #18-33, 16 scans) Frag=175.0V 1f-383.d
x10 6
* 122.0968
217.0690
180.0872
250.9731
323.1441
([C24 H18 O]+H)+
363.1363
([C24 H20 O2]+Na)+
Counts vs. Mass-to-Charge (m/z)

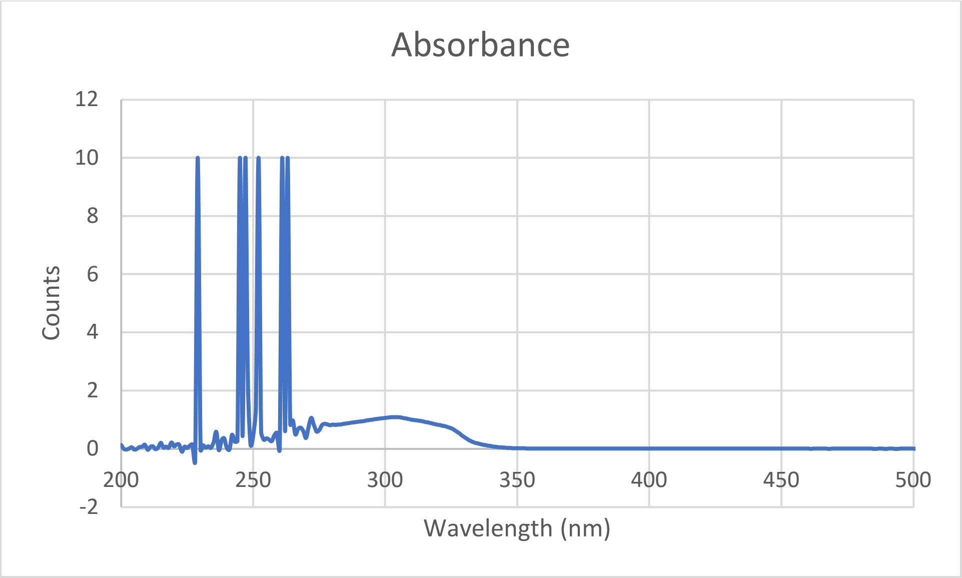
Absorbance
12
10
8
6
4
2
0
-2
Counts
200
250
300
350
400
450
500
Wavelength (nm)

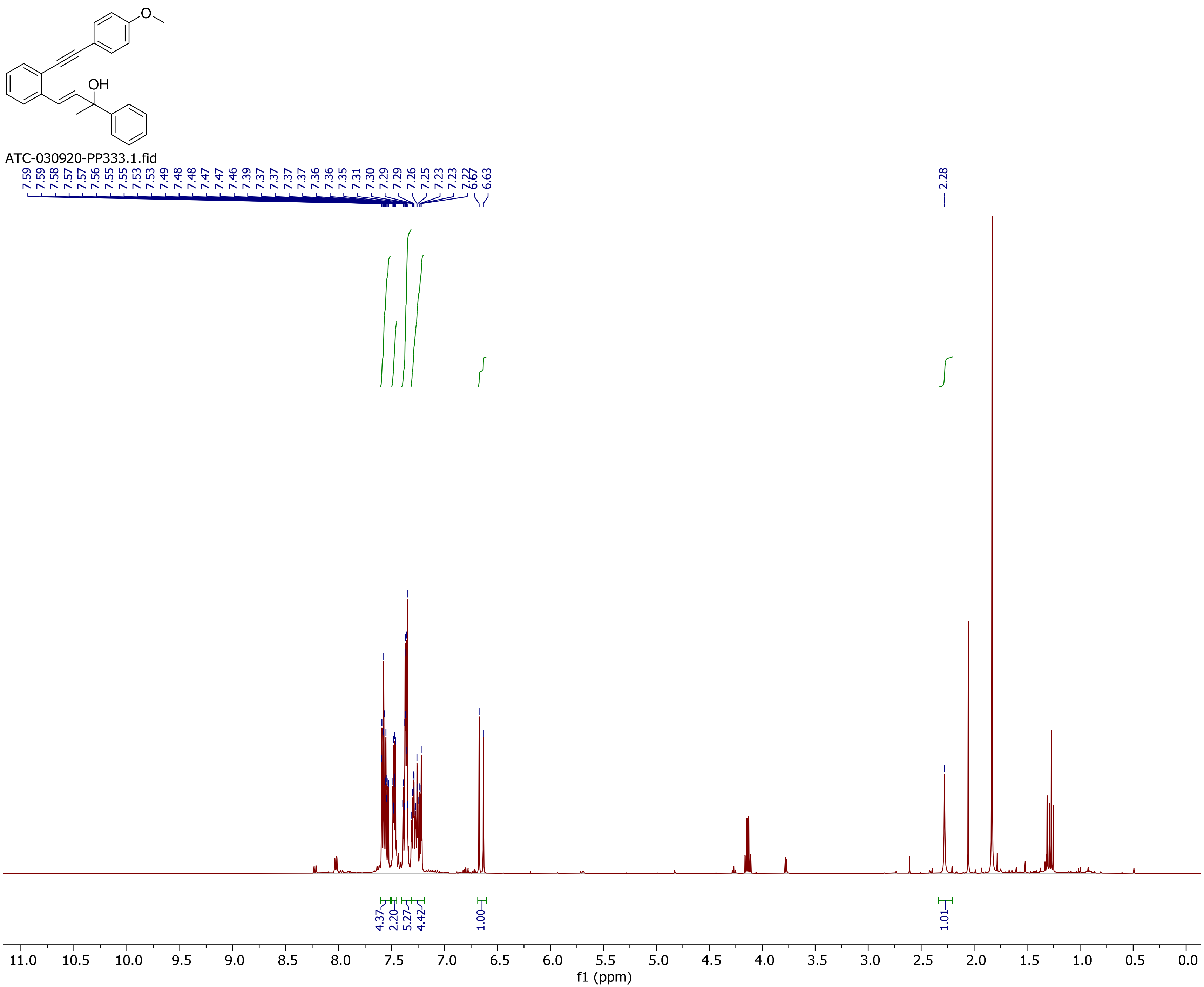

OH
O
ATC-030920-PP333.1.fid
7.59
7.59
7.58
7.57
7.57
7.56
7.55
7.55
7.53
7.53
7.49
7.48
7.47
7.47
7.46
7.39
7.37
7.37
7.37
7.37
7.36
7.35
7.31
7.30
7.29
7.29
7.26
7.25
7.23
7.23
7.22
6.67
6.63
2.28
4.37
2.20
5.27
4.42
1.00
1.01
11.0
10.5
10.0
9.5
9.0
8.5
8.0
7.5
7.0
6.5
6.0
5.5
5.0
4.5
4.0
3.5
3.0
2.5
2.0
1.5
1.0
0.5
0.0
f1 (ppm)

ATC-052820-CP333-C13.1.fid

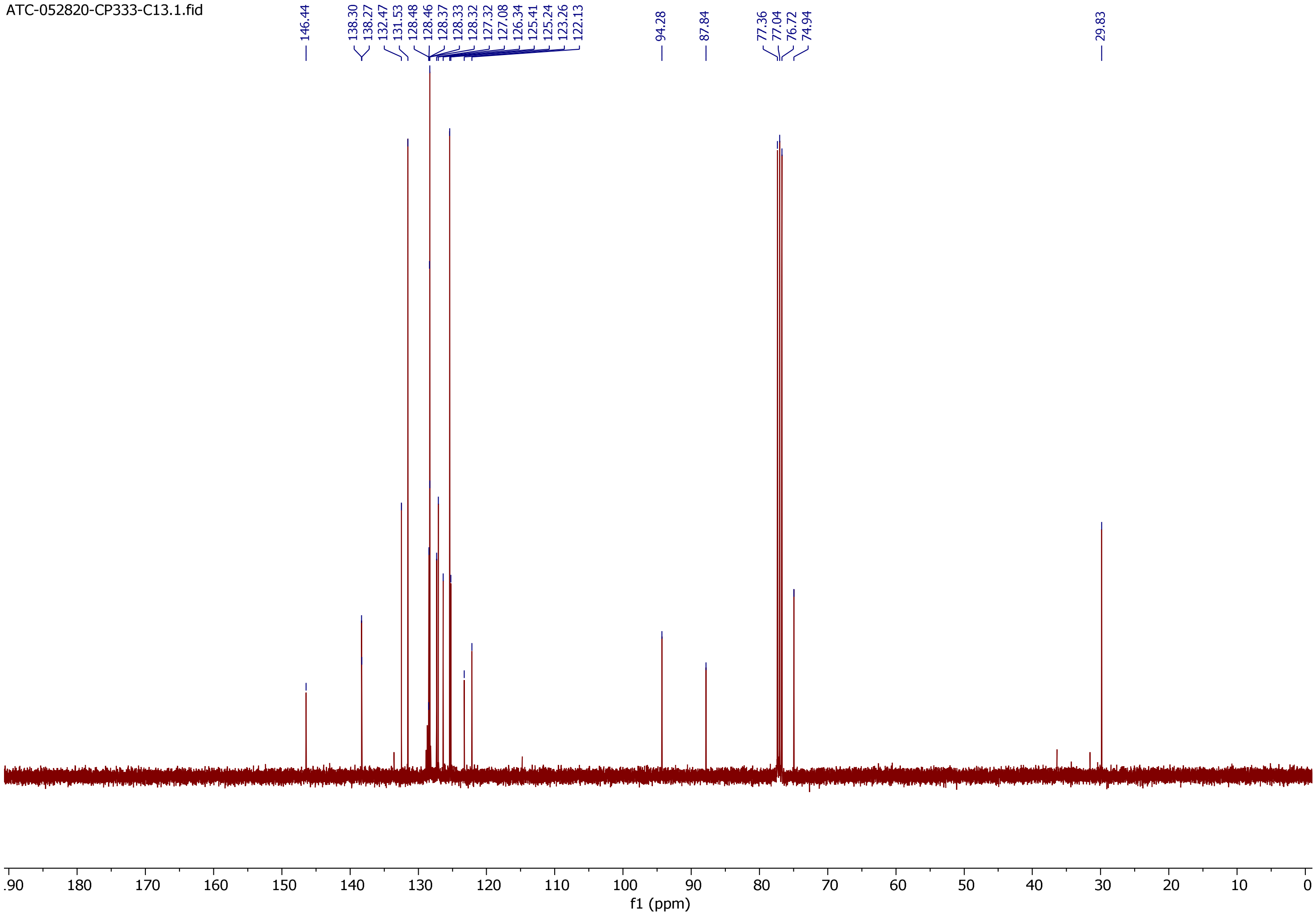

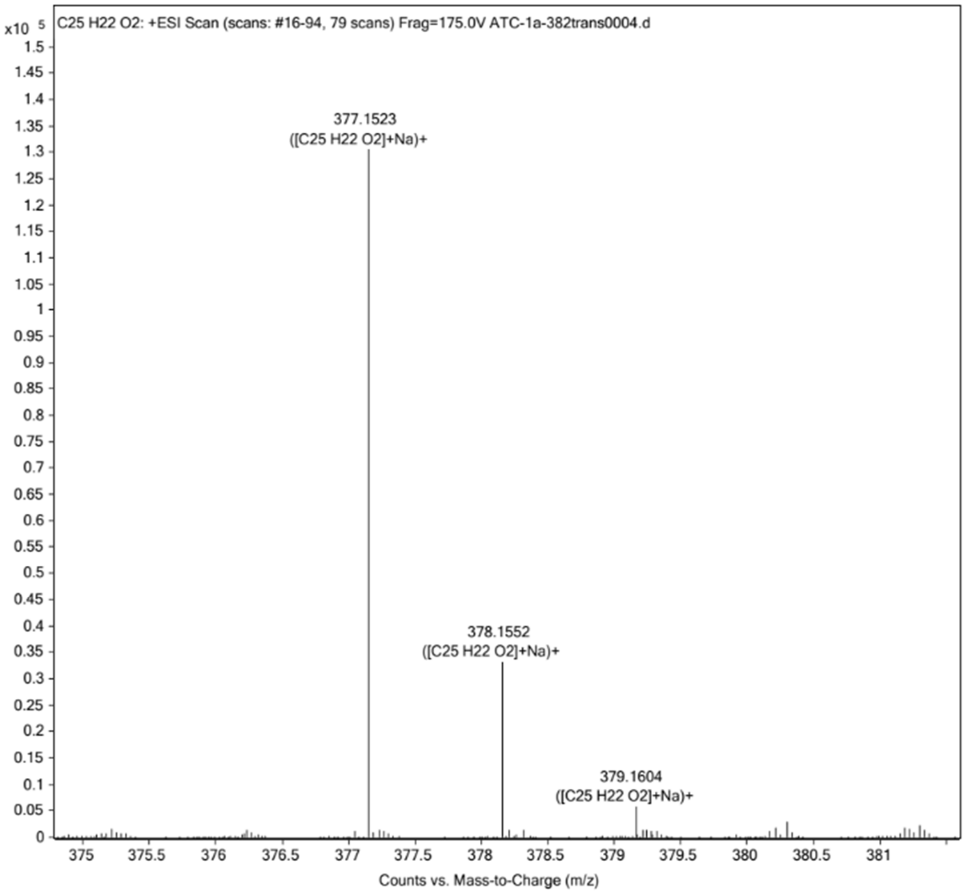

x10 5
C25 H22 O2: +ESI Scan (scans: #16-94, 79 scans) Frag=175.0V ATC-1a-382trans0004.d
377.1523
([C25 H22 O2]+Na)+
378.1552
([C25 H22 O2]+Na)+
379.1604
([C25 H22 O2]+Na)+
Counts vs. Mass-to-Charge (m/z)

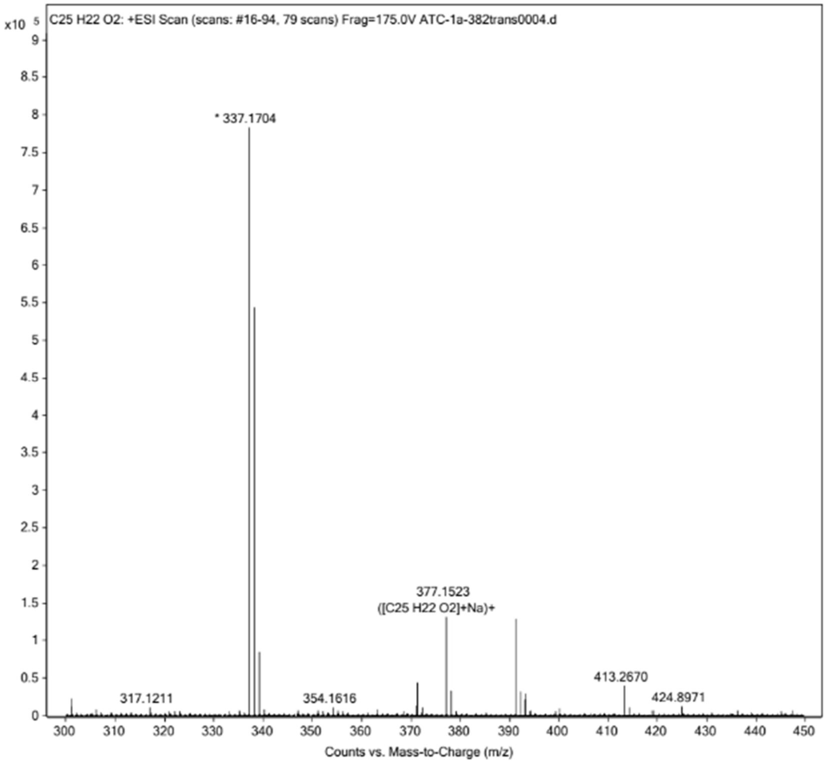

x10 5
C25 H22 O2: +ESI Scan (scans: #16-94, 79 scans) Frag=175.0V ATC-1a-382trans0004.d
* 337.1704
377.1523
([C25 H22 O2]+Na)+
317.1211
354.1616
413.2670
424.8971
Counts vs. Mass-to-Charge (m/z)

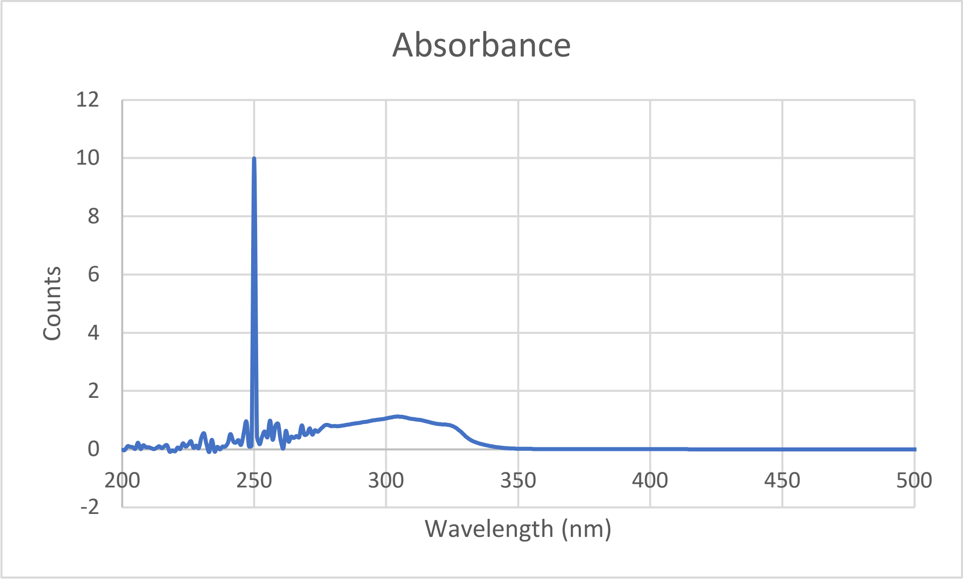

Absorbance
Counts
Wavelength (nm)

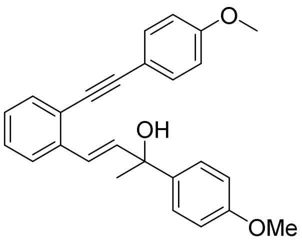

ATC-030920-PP331.1.fid

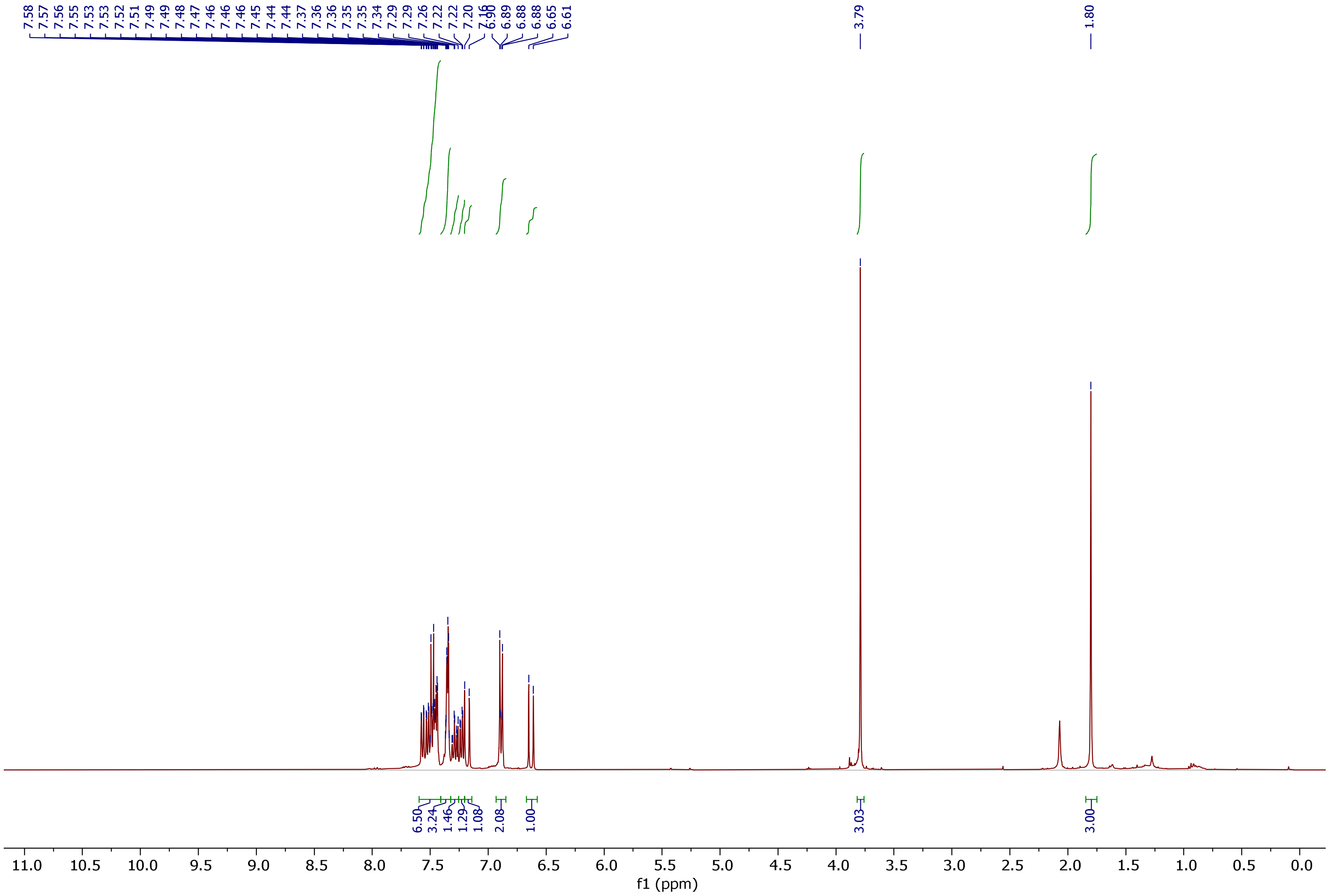

[1]H broad Peak at 2.1 = 1H from OH

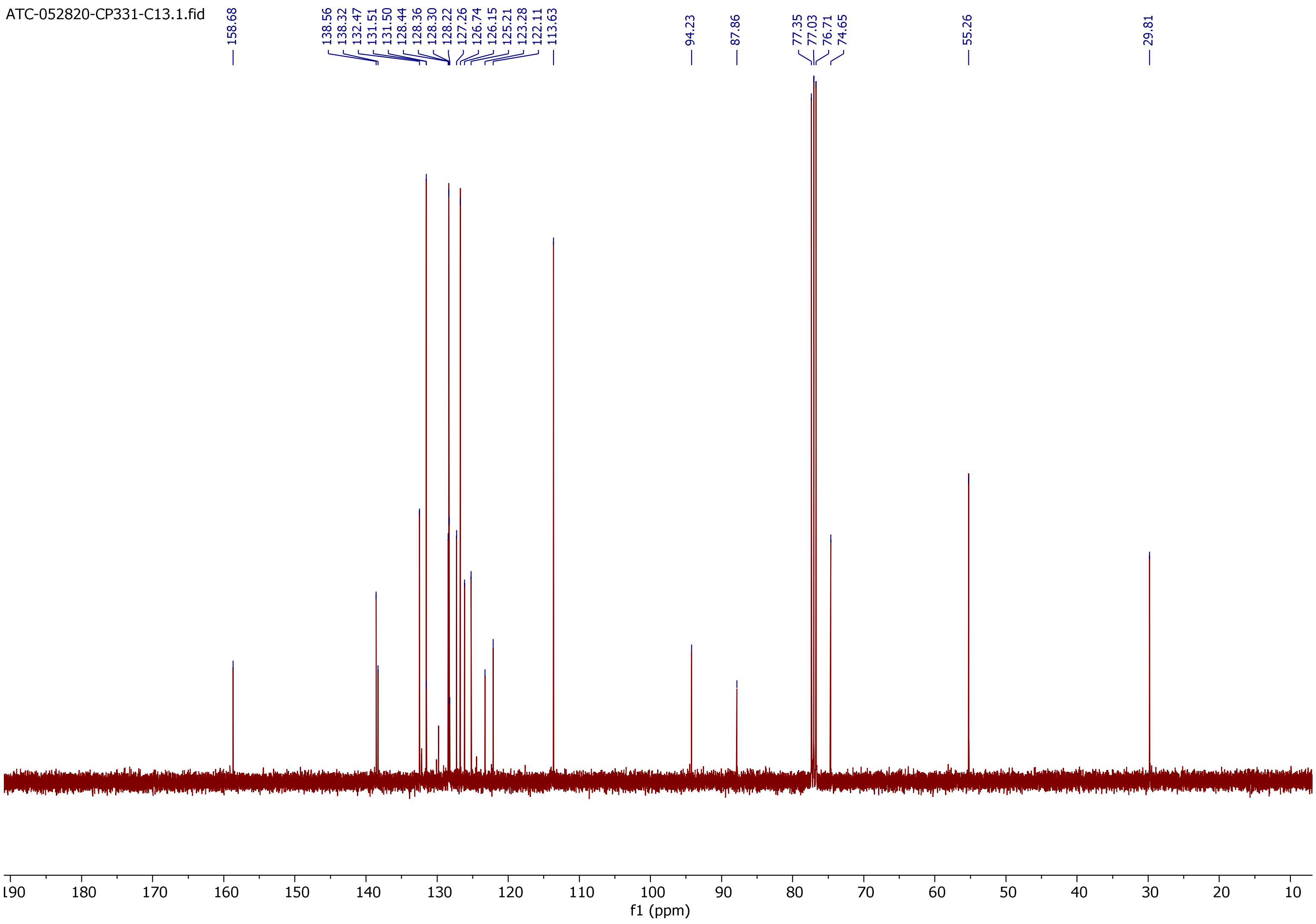

ATC-052820-CP331-C13.1.fid
158.68
138.56
138.32
132.47
131.51
131.50
128.44
128.36
128.30
128.22
127.26
126.74
126.15
125.21
123.28
122.11
113.63
94.23
87.86
77.35
77.03
76.71
74.65
55.26
29.81
190
180
170
160
150
140
130
120
110
100
90
80
70
60
50
40
30
20
10
f1 (ppm)

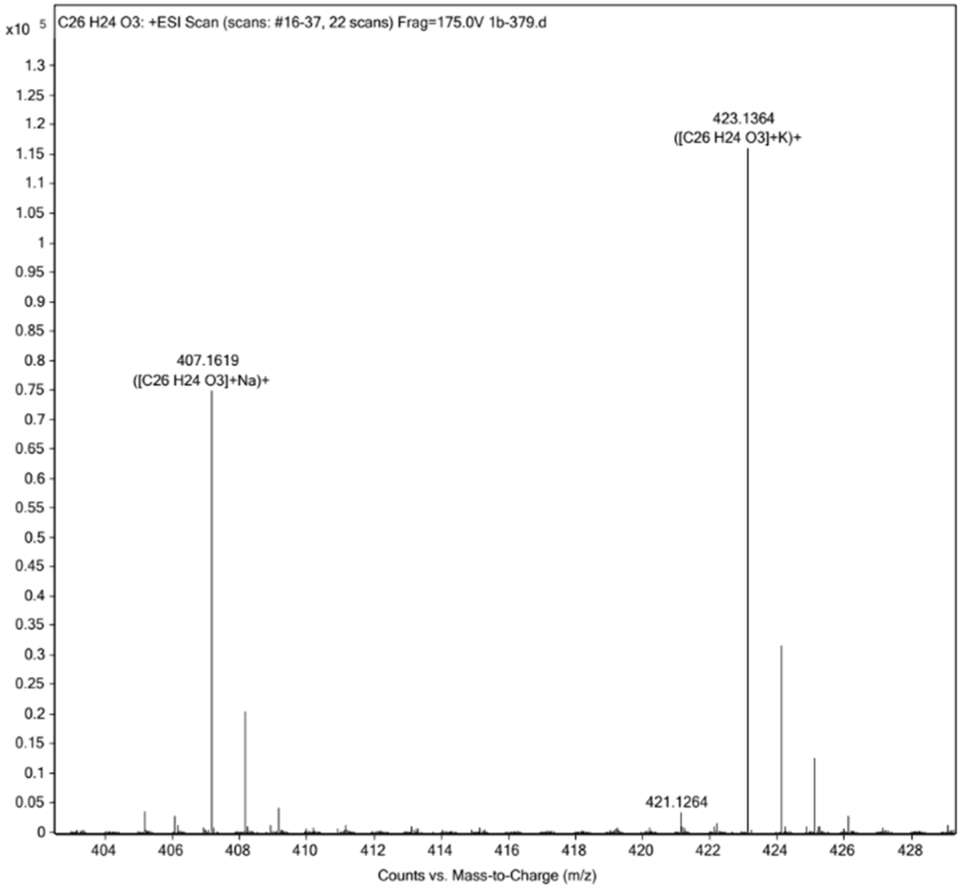

x10 5
C26 H24 O3: +ESI Scan (scans: #16-37, 22 scans) Frag=175.0V 1b-379.d
423.1364
([C26 H24 O3]+K)+
407.1619
([C26 H24 O3]+Na)+
421.1264
Counts vs. Mass-to-Charge (m/z)

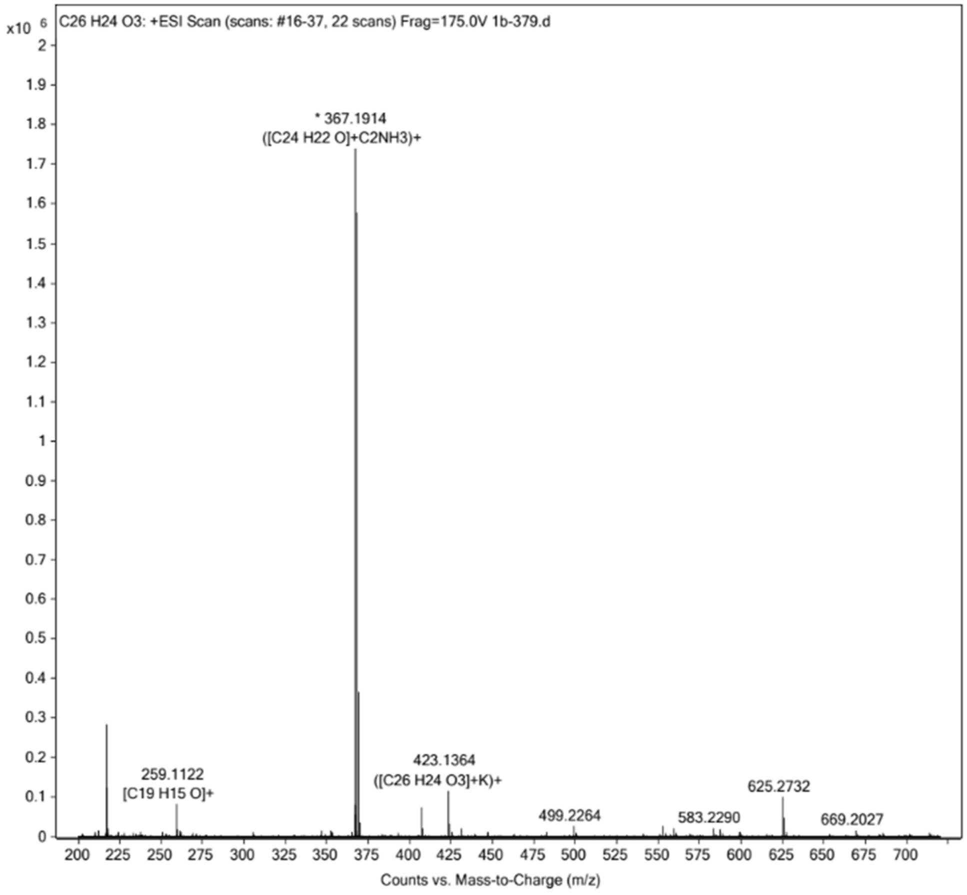

C26 H24 O3: +ESI Scan (scans: #16-37, 22 scans) Frag=175.0V 1b-379.d
x10 6
* 367.1914
([C24 H22 O]+C2NH3)+
259.1122
[C19 H15 O]+
423.1364
([C26 H24 O3]+K)+
499.2264
583.2290
625.2732
669.2027
Counts vs. Mass-to-Charge (m/z)

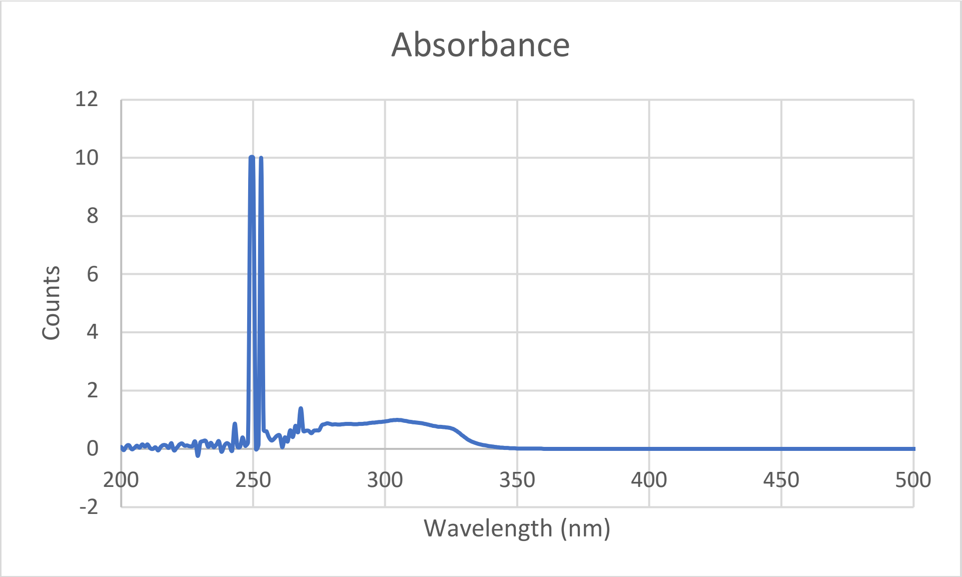
Absorbance
Counts
Wavelength (nm)
12
10
8
6
4
2
0
-2
200
250
300
350
400
450
500

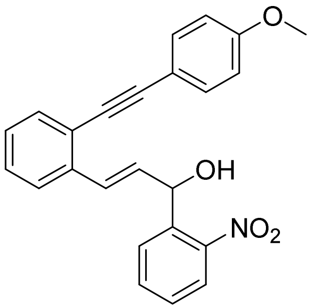
O
OH
NO2

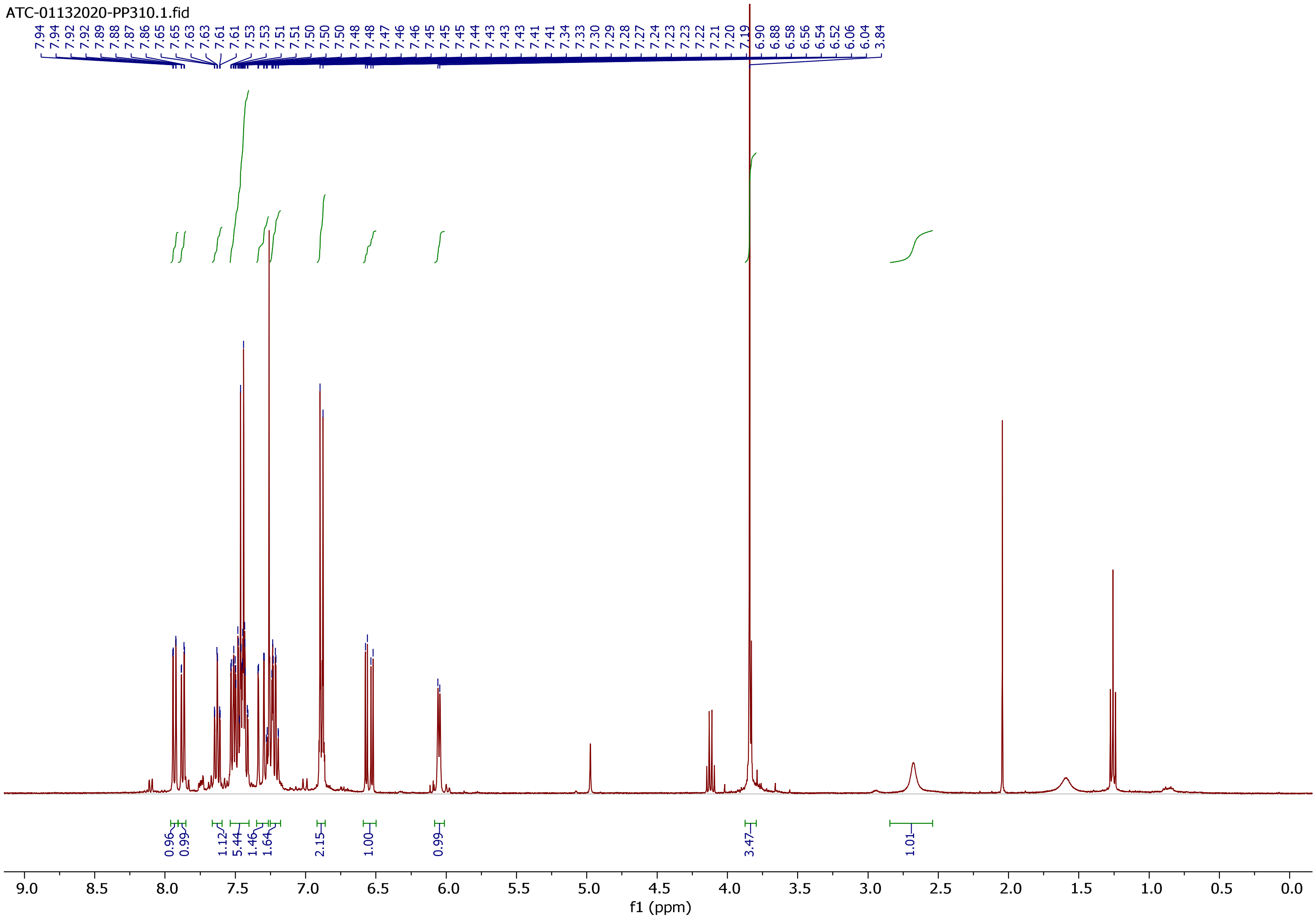
ATC-01132020-PP310.1.fid
7.94 7.94 7.92 7.92 7.89 7.88 7.87 7.86 7.65 7.65 7.63 7.63 7.61 7.61 7.53 7.53 7.51 7.51 7.50 7.50 7.50 7.48 7.48 7.47 7.46 7.46 7.45 7.45 7.45 7.44 7.43 7.43 7.43 7.41 7.41 7.34 7.33 7.30 7.29 7.28 7.27 7.24 7.23 7.23 7.22 7.21 7.20 7.19 6.90 6.88 6.58 6.56 6.54 6.52 6.06 6.04 3.84
0.96 0.99 1.12 5.44 1.46 1.64 2.15 1.00 0.99 3.47 1.01
9.0 8.5 8.0 7.5 7.0 6.5 6.0 5.5 5.0 4.5 4.0 3.5 3.0 2.5 2.0 1.5 1.0 0.5 0.0
f1 (ppm)

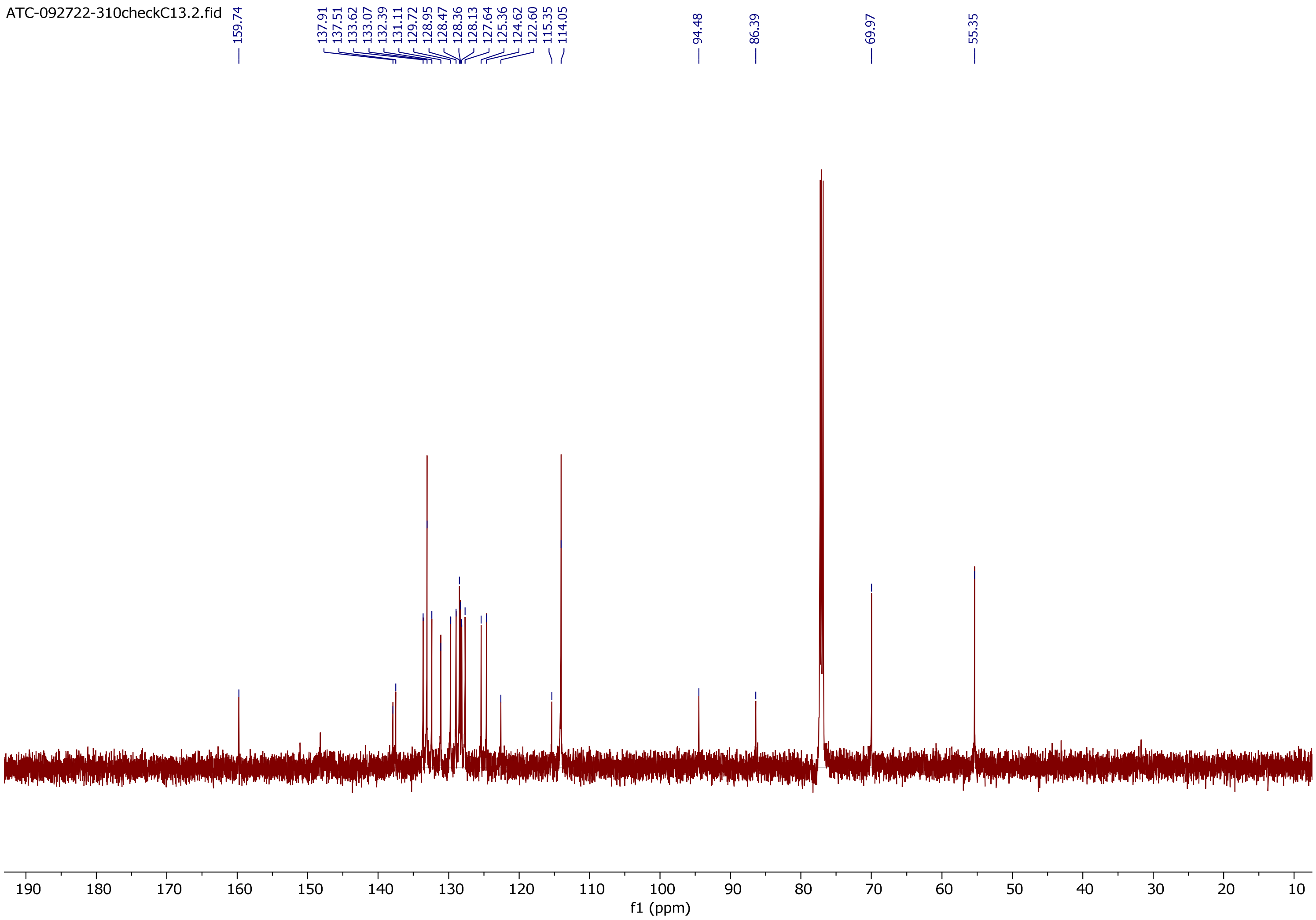
ATC-092722-310checkC13.2.fid
159.74
137.91
137.51
133.62
133.07
132.39
131.11
129.72
128.95
128.47
128.36
128.13
127.64
125.36
124.62
122.60
115.35
114.05
94.48
86.39
69.97
55.35
190 180 170 160 150 140 130 120 110 100 90 80 70 60 50 40 30 20 10
f1 (ppm)

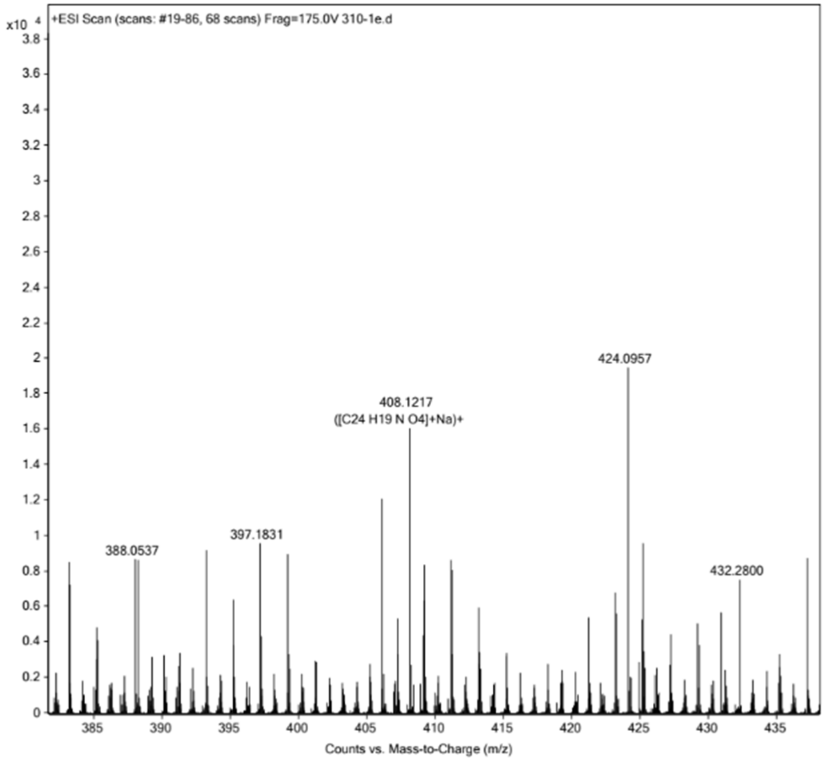

+ESI Scan (scans: #19-86, 68 scans) Frag=175.0V 310-1e.d
x10 4
3.8
3.6
3.4
3.2
3
2.8
2.6
2.4
2.2
2
1.8
1.6
1.4
1.2
1
0.8
0.6
0.4
0.2
0
424.0957
408.1217
([C24 H19 N O4]+Na)+
388.0537
397.1831
432.2800
385
390
395
400
405
410
415
420
425
430
435
Counts vs. Mass-to-Charge (m/z)

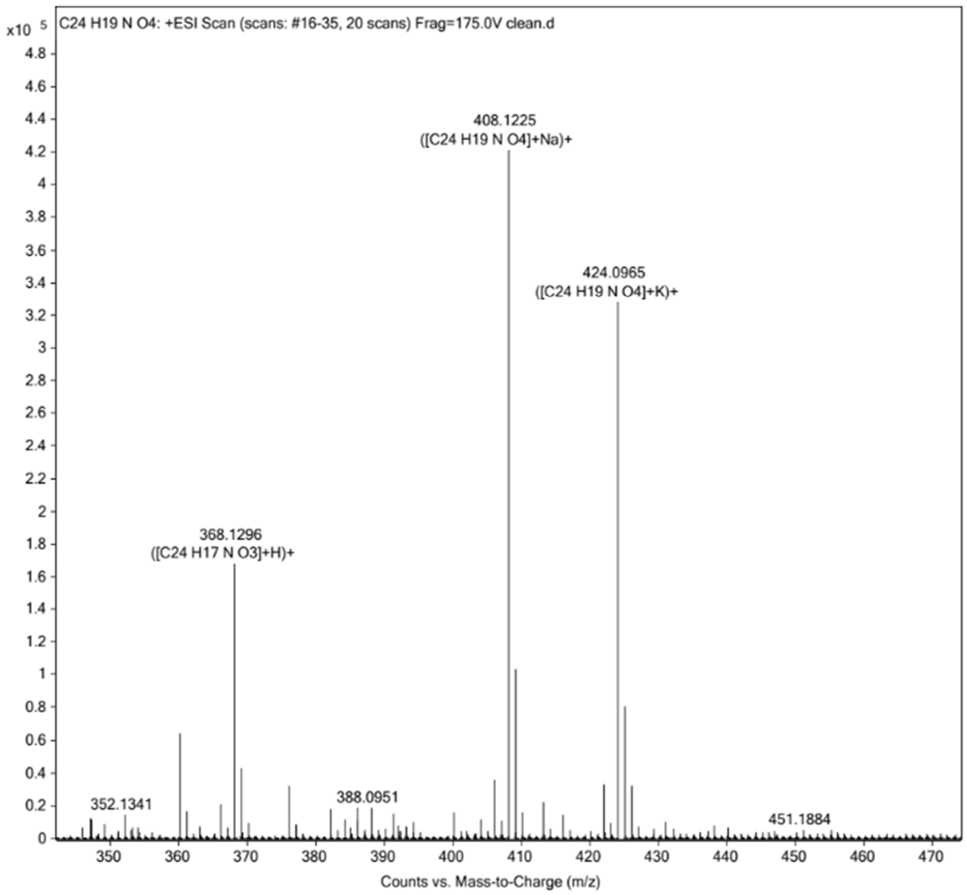

IV. References

(1) Mohamed, R. K.; Mondal, S.; Jorner, K.; Delgado, T. F.; Lobodin, V. V.; Ottosson, H.; Alabugin, I. V. The Missing C1–C5 Cycloaromatization Reaction: Triplet State Antiaromaticity Relief and Self-Terminating Photorelease of Formaldehyde for Synthesis of Fulvenes from Enynes. *J. Am. Chem. Soc.* **2015**, *137* (49), 15441–15450. https://doi.org/10.1021/jacs.5b07448.

(2) Sheehan, J. C.; Umezawa, K. Phenacyl Photosensitive Blocking Groups. *J. Org. Chem.* **1973**, *38* (21), 3771–3774. https://doi.org/10.1021/jo00961a027.

(3) Literák, J.; Dostálová, A.; Klán, P. Chain Mechanism in the Photocleavage of Phenacyl and Pyridacyl Esters in the Presence of Hydrogen Donors. *J. Org. Chem.* **2006**, *71* (2), 713–723. https://doi.org/10.1021/jo0521551.

(4) Patchornik, A.; Amit, B.; Woodward, R. B. Photosensitive Protecting Groups. *J. Am. Chem. Soc.* **1970**, *92* (21), 6333–6335. https://doi.org/10.1021/ja00724a041.

(5) Walker, J. W.; Reid, G. P.; McCray, J. A.; Trentham, D. R. Photolabile 1-(2-Nitrophenyl)Ethyl Phosphate Esters of Adenine Nucleotide Analogs. Synthesis and Mechanism of Photolysis. *J. Am. Chem. Soc.* **1988**, *110* (21), 7170–7177. https://doi.org/10.1021/ja00229a036.

(6) Givens, R. S.; Matuszewski, B. Photochemistry of Phosphate Esters: An Efficient Method for the Generation of Electrophiles. *J. Am. Chem. Soc.* **1984**, *106* (22), 6860–6861. https://doi.org/10.1021/ja00334a075.
(7) Lin, W.; Lawrence, D. S. A Strategy for the Construction of Caged Diols Using a Photolabile Protecting Group. *J. Org. Chem.* **2002**, *67* (8), 2723–2726. https://doi.org/10.1021/jo0163851.
(8) Barltrop, J. A.; Schofield, P. Photosensitive Protecting Groups. *Tetrahedron Lett.* **1962**, *3* (16), 697–699. https://doi.org/10.1016/S0040-4039(00)70935-X.
(9) Wang, P.; Wang, Y.; Hu, H.; Spencer, C.; Liang, X.; Pan, L. Sequential Removal of Photolabile Protecting Groups for Carbonyls with Controlled Wavelength. *J. Org. Chem.* **2008**, *73* (16), 6152–6157. https://doi.org/10.1021/jo8008275.
(10) Karas, L. J.; Campbell, A. T.; Alabugin, I. V.; Wu, J. I. Antiaromaticity Gain Activates Tropone and Nonbenzenoid Aromatics as Normal-Electron-Demand Diels–Alder Dienes. *Org. Lett.* **2020**, *22* (18), 7083–7087. https://doi.org/10.1021/acs.orglett.0c02343.
(11) Wu, C.-H.; Karas, L. J.; Ottosson, H.; Wu, J. I.-C. Excited-State Proton Transfer Relieves Antiaromaticity in Molecules. *Proc. Natl. Acad. Sci.* **2019**, *116* (41), 20303–20308. https://doi.org/10.1073/pnas.1908516116.
(12) Slanina, T.; Ayub, R.; Toldo, J.; Sundell, J.; Rabten, W.; Nicaso, M.; Alabugin, I.; Fdez. Galván, I.; Gupta, A. K.; Lindh, R.; Orthaber, A.; Lewis, R. J.; Grönberg, G.; Bergman, J.; Ottosson, H. Impact of Excited-State Antiaromaticity Relief in a Fundamental Benzene Photoreaction Leading to Substituted Bicyclo[3.1.0]Hexenes. *J. Am. Chem. Soc.* **2020**, *142* (25), 10942–10954. https://doi.org/10.1021/jacs.9b13769.
(13) Fernández, I.; Alabugin, I. V.; Campbell, A. T. Aromaticity - Modern Computational Methods and Applications; Elsevier, 2021.
(14) Lage Robles, J.; Bochet, C. G. Photochemical Release of Aldehydes from α-Acetoxy Nitroveratryl Ethers. *Org. Lett.* **2005**, *7* (16), 3545–3547. https://doi.org/10.1021/ol051280w.
(15) Gravel, D.; Murray, S.; Ladouceur, G. O-Nitrobenzyl Alcohol, a Simple and Efficient Reagent for the Photoreversible Protection of Aldehydes and Ketones. *J. Chem. Soc. Chem. Commun.* **1985**, No. 24, 1828–1829. https://doi.org/10.1039/C39850001828.
(16) Wang, P.; Hu, H.; Wang, Y. Novel Photolabile Protecting Group for Carbonyl Compounds. *Org. Lett.* **2007**, *9* (8), 1533–1535. https://doi.org/10.1021/ol070346f.
(17) McHale, W. A.; Kutateladze, A. G. An Efficient Photo-SET-Induced Cleavage of Dithiane−Carbonyl Adducts and Its Relevance to the Development of Photoremovable Protecting Groups for Ketones and Aldehydes. *J. Org. Chem.* **1998**, *63* (26), 9924–9931. https://doi.org/10.1021/jo981697y.
(18) Alabugin, I. V.; Manoharan, M. Radical-Anionic Cyclizations of Enediynes: Remarkable Effects of Benzannelation and Remote Substituents on Cyclorearomatization Reactions. *J. Am. Chem. Soc.* **2003**, *125* (15), 4495–4509. https://doi.org/10.1021/ja029664u.
(19) Baird, N. C. Quantum Organic Photochemistry. II. Resonance and Aromaticity in the Lowest 3.Pi..Pi.* State of Cyclic Hydrocarbons. *J. Am. Chem. Soc.* **1972**, *94* (14), 4941–4948. https://doi.org/10.1021/ja00769a025.
(20) Augé, J.; Boucard, V.; Gil, R.; Lubin-Germain, N.; Picard, J.; Uziel, J. An Alternative Procedure in the Takai Reaction Using Chromium(III) Chloride Hexahydrate as a Convenient Source of Chromium(II). *Synth. Commun.* **2003**, *33* (21), 3733–3739. https://doi.org/10.1081/SCC-120025182.
(21) Neumann, H.; Seebach, D. Stereospecific Preparation of Terminal Vinyllithium Derivatives by Br/Li-Exchange with t-Butyllithium. *Tetrahedron Lett.* **1976**, *17* (52), 4839–4842. https://doi.org/10.1016/S0040-4039(00)78926-X.
(22) Palei, B. A.; Gavrilenko, V. V.; Zakharkin, L. I. Comparative Reactivity of Alkyl, Vinyl, and Acetylene Derivatives of Aluminum. *Bull. Acad. Sci. USSR Div. Chem. Sci.* **1969**, *18* (12), 2590–2595. https://doi.org/10.1007/BF00912549.
